# Sodium levels and grazing pressure shape natural communities of the intracellular pathogen* Legionella*

**DOI:** 10.1186/s40168-023-01611-0

**Published:** 2023-07-31

**Authors:** Oded Bergman, Yaron Be’eri-Shlevin, Shira Ninio

**Affiliations:** Kinneret Limnological Laboratory (KLL), Israel Oceanographic and Limnological Research (IOLR), P.O. Box 447, 49500 Migdal, Israel

**Keywords:** *Legionella*, Sodium, Natural springs, Microbiome, Saline environment, Dot/Icm, Extreme environment, Intracellular pathogen, Amoeba-resistant bacteria

## Abstract

**Background:**

*Legionella* are parasites of freshwater protozoa, responsible for Legionellosis. *Legionella* can be found in a variety of aquatic environments, including rivers, lakes, and springs, as well as in engineered water systems where they can potentially lead to human disease outbreaks. *Legionella* are considered to be predominantly freshwater organisms with a limited ability to proliferate in saline environments. Exposure of *Legionella* to high sodium concentrations inhibits growth and virulence of laboratory strains, particularly under elevated temperatures. Nonetheless, *Legionella* have been identified in some saline environments where they likely interact with various protozoan hosts. In this work, we examine how these selection pressures, sodium and grazing, help shape *Legionella* ecology within natural environments. Utilizing *Legionella*-specific primers targeting a variable region of the *Legionella* 16S rRNA gene, we characterized *Legionella* abundance, diversity, and community composition in natural spring clusters of varying sodium concentrations, focusing on high sodium concentrations and elevated temperatures.

**Results:**

We observed the highest abundance of *Legionella* in spring clusters of high salinity, particularly in combination with elevated temperatures. *Legionella* abundance was strongly related to sodium concentrations. The *Legionella* community structure in saline environments was characterized by relatively low diversity, compared to spring clusters of lower salinity. The community composition in high salinity was characterized by few dominant *Legionella* genotypes, not related to previously described species. Protozoan microbial community structure and composition patterns resembled those of *Legionella*, suggesting a common response to similar selection pressures. We examined *Legionella* co-occurrence with potential protozoan hosts and found associations with *Ciliophora* and *Amoebozoa* representatives.

**Conclusions:**

Our results indicate that selection forces in saline environments favor a small yet dominant group of *Legionella* species that are not closely related to known species. These novel environmental genotypes interact with various protozoan hosts, under environmental conditions of high salinity. Our findings suggest that alternative survival mechanisms are utilized by these species, representing mechanisms distinct from those of well-studied laboratory strains. Our study demonstrate how salinity can shape communities of opportunistic pathogens and their hosts, in natural environments, shedding light on evolutionary forces acting within these complex environments.

Video Abstract

**Supplementary Information:**

The online version contains supplementary material available at 10.1186/s40168-023-01611-0.

## Background

The genus *Legionella* are Gram-negative aerobic bacteria, responsible for Legionellosis [1], a sporadically occurring respiratory illness, contracted predominantly through inhalation of water aerosols [2]. To date, about 70 species have been identified [1, 3], many of which are known human pathogens [4]. Not surprisingly, the vast majority of studies focus mainly on clinical isolates and occurrence in man-made facilities [5–7]. In the environment, many *Legionella* species multiply intracellularly, using various protists as hosts, including amoeba [8, 9], ciliates [9, 10], and slime molds [11]. Intracellular survival of *Legionella* requires the function of a specialized secretion system called Dot/Icm, which delivers a large set of effector proteins into the host cell [12]. The delivered effectors act to subvert hot cellular processes and promote survival and proliferation. The Dot/Icm system is essential for *Legionella* survival in amoebae as well as in human macrophages [13].

*Legionella* occur in a variety of aquatic environments, including rivers, lakes, thermal and saline springs, and groundwater [14–22]. Such ecosystems may act as reservoirs for the pathogen, presenting potential risks for leakage into human-related structures and Legionellosis outbreaks [23, 24]. Nevertheless, comprehensive investigations related to *Legionella* natural occurrence, diversity, related environmental conditions, and co-occurrence with potential hosts are scarce.

Few investigations compared *Legionella* spp. community composition and diversity, between natural environments and anthropogenic settings. These indicated significant differences between the ecosystems [20, 25, 26]. Importantly, reported diversity in natural aquatic environments is high, with many of the identified *Legionella* sequence variants distinct from previously described species. These findings indicate that *Legionella* natural diversity is underestimated. Furthermore, differences between laboratory strains and *Legionella* strains isolated from natural environments is not limited to genetic variations and is also evident by different resistance to potential environmental stressors (e.g., temperature and oxidative stress) [27]. *Legionella* can persist in a wide range of environmental conditions, including pH, temperature, and NaCl concentrations [28, 29] and in nature they frequently occupy oligotrophic environments [30]. Paradoxically, isolation requires highly permissive media, which includes various supplements and an optimized temperature, suggesting a fastidious nature [31]. This discrepancy can be explained by the ability of *Legionella* to persist in biofilms [28, 32, 33], their life cycle as intracellular pathogens [6] and by the inability of some of the *Legionella* species to grow, under standard culture conditions [34].

Only a limited number of studies focused on *Legionella* occurrence in saline environments [14]. The reported presence of *Legionella* in seawater has traditionally been attributed to sewage spillage and other anthropogenic influences [35, 36]. Thus, *Legionella* has been associated with freshwater environments, as it was believed it cannot proliferate in seawater [34]. However, several studies reported *L. pneumophila* is able to persist in seawater [29, 35, 37, 38]. In addition, the presence of diverse *Legionella* communities has been reported in amoeba cultures isolated from saline environments [35]. Although isolation from saline environments has proven to be a particularly challenging endeavor, two successful attempts have been reported, of *L. pneumophila* [39] and of a novel species *L. tunisiensis* following co-culture with a laboratory strain of amoeba [22]. Together, these findings suggest that *Legionella* spp. can persist in saline, sodium-rich, environments and interact with protozoan hosts within these environments. Nonetheless, it has been well established that the expression of *Legionella* virulence traits is required for successful host infection, yet renders the bacteria sodium-sensitive under lab conditions, both in solid media [40], as well as in broth [37]. It has been postulated that sodium sensitivity is a result of leakage of sodium ions through the Dot/Icm secretion system [37, 40, 41]. It is therefore likely that *Legionella* species that have adapted to survive in sodium-rich environments, in the presence of sodium-resistant protozoa, may be utilizing novel survival mechanisms distinct from those described for freshwater *Legionella*.

In this work, we sought to delineate how selection pressure excreted by high sodium in combination with grazing—help shape *Legionella* ecology within natural environments. We hypothesized that *Legionella* spp. abundance, community composition, and diversity will differ between environments of varying sodium concentrations. To this end, we sampled natural aquatic environments belonging to four spring clusters representing a spectrum of sodium concentrations, over a period of 2 years. We performed in-depth analysis of various environmental factors and assessed *Legionella* abundance and community composition using qPCR and next-generation amplicon-based sequencing. Co-occurrence network analysis indicated potential *Legionella* interactions with various *Ciliophora* and *Amoebozoa* representatives. Our results show that sodium concentrations are a major driver of *Legionella* abundance and community composition. Employing a random forest machine learning algorithm, we found novel and dominant *Legionella* genotypes characteristic of sodium-rich environments. Future work utilizing sodium-resistant genotypes could reveal new molecular mechanisms and proliferation strategies mediating intracellular survival of bacterial pathogens.

## Methods

### Study sites

**Fig. 1:**
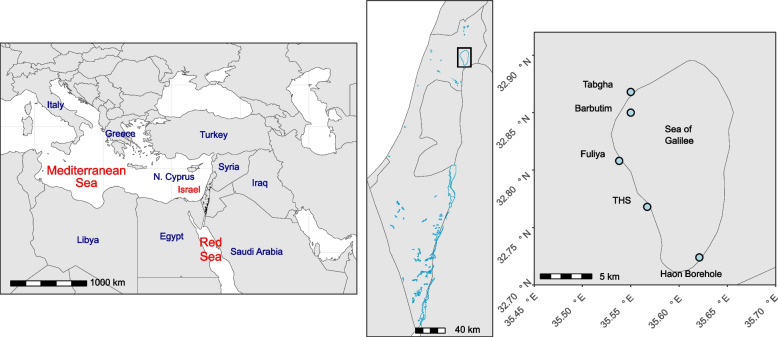
Map of study sites. The sea of Galilee (right panel), located in northern Israel (middle panel), the Middle East (left panel). The study sites included saline springs within and around the Sea of Galilee (right panel) and are divided into four spring clusters: Tiberias Hot Springs (THS), Tabgha springs, Fuliya springs, and Haon borehole. The Barbutim spring is located within the sea of galilee and is related to the Tabgha cluster. Haon borehole is comprised of a single sampling station. Detailed maps of Tabgha, Fuliya, and THS individual sampling stations are presented in Supplementary Fig. 1

Lake Kinneret (The Sea of Galilee, 32° 42′–32° 55′ N; 35° 31′–35°39′ E, Fig. 1) is located in the northern part of the Afro-Syrian Rift and is surrounded by numerous shoreline springs and boreholes. Comprised of a mixture of upward flows of concentrated brine-water and ground-level freshwater aquifers [42], these brines are classified, among others, according to location and geochemical characteristics into distinct clusters. The western Kinneret brines are grouped into three main clusters presenting distinct chemical characteristics (e.g., temperature and salinity) [42–44]. From each cluster, several representative springs were sampled: Tiberias Hot Springs (THS, *n* = 3), Tabgha (*n* = 5), and Fuliya (*n* = 5) (Fig. 1 and Supplementary Fig. 1), with the offshore Barbutim station grouped into the Tabgha cluster. Haon borehole on the south-eastern shore of the lake represents a separate cluster, related to THS [42].

### Sample collection and chemical analysis

Each station was sampled on September 2017, January 2018, June 2018, October 2018, and January 2019. Surface water (triplicates in 1-L sterile plastic bottles) and biofilm (scraped from submerged rocks using a sterile scalpel in 15-ml sterile falcon-tubes) were transferred to the laboratory within 2–3 h. After mixing, 1 L of water was used for chemical analysis and the remaining water was filtered on 47-mm pore size diameter 0.22 μm (Supor Polycarbonate Membrane Disc Filters; Pall, MI, USA). Filters were immediately stored at − 80 °C until further use, together with the biofilm samples. Station Fuliya2 became submerged due to changes in lake water levels, precluding the planned September 2019 sampling. Biofilm sampling was not possible for Nur (excluding September 2017), Haon borehole, Barbutim, and Tiberias Pump stations. Temperature measurements were performed in situ using a mercury-in-glass thermometer. The physico-chemical measurements of pH, Cl, turbidity, conductivity, total and dissolved phosphorus (TP and TDP), total and dissolved nitrogen (TN and TDN), NO_3_^−^, SO4^−2^, and total Fe were performed following standard methods (APHA 2005) and are further described by Nishri et al. [45]. Sodium concentrations were calculated from Cl levels, based on published Na ⁄ Cl Molar ratios, characteristic of each spring cluster [42, 44].

### DNA extraction

DNA was extracted using the Power-Water and Power-Soil DNA Isolation Kits (QIAGEN, Hilden, Germany; prior MoBio, CA, USA), according to the manufacturer’s instructions. 0.25 g of biofilm per sample was used for the Power-Soil kit. The specified alternative lysis method was performed, with 10 min incubation at 65 °C. To increased DNA recovery, elution buffer was pre-warmed to 70 °C. To obtain higher-quality DNA, elutes were precipitated with sodium acetate (3 M, pH 5.2) and rinsed in 70% pure ethanol (Merck) and stored at − 80 °C.

### Quantitative-PCR (qPCR)

Quantification of *Legionella* spp. abundance was performed in water (*n* = 64) and biofilm (*n* = 50) samples as previously described [46], using *Legionella-*specific primers designed for the 16S rRNA gene: 5′-TAGTGGAATTTCCGGTGTA-3′ (LegF1c, forward) and 5′-CCAACAGCTAGTTGACATC-3′ (LegR1c, reverse), with the ([6FAM]-CGGCTACCTGGCCTAATACTGA-[BHQ1]) probe. qPCR was performed using TaqMan™ Fast Advanced Master Mix (Applied Biosystems, CA, USA), in a 20 μl reaction mix, containing 2 μl DNA, 10 μl of × 2 buffer, 2 μl each primer (at a final concentration of 500 nM), 1 μl TaqMan probe, and 3 μl molecular water (Biological Industries, Israel). Reactions were performed using Rotor Gene 6000, software version 1.7 (Corbett Research, UK), with the following conditions: 2 min of incubation at 50 °C, followed by 10 min activation at 95 °C and 40 cycles of denaturation 15 s at 95 °C, with a combined annealing/extension step of 60 s at 50 °C. Standard calibration curves with known concentration of the target gene (derived from *L. pneumophila* serogroup 1, wild-type strain Lp01) vs cycle threshold (CT) were constructed to determine gene concentration.

Heatmap showing K-means clustering of qPCR data was generated in R (version 4.0.3) using the package Pheatmap [47], with samples as rows (clustered, *n* = 22) and collection date as column (*n* = 5). Optimal number of clusters was determined and visualized using the package factoextra [48] (Supplementary Fig. 4 and Supplementary Table 8).

### Characterization of spring cluster physico-chemical properties and linear mixed model effect (LMM) with *Legionella* spp. abundance

Water physico-chemical characterization of the four spring clusters was performed by separate principal component analysis (PCA) of water (*n* = 55) and biofilm (*n* = 39) samples, using the R function prcomp [49]. Only samples between January 2018 and January 2019 were included in the analysis, as several chemical parameters were not available for samples taken during September 2017. The centered-scaled log + 1 values of all measured chemical parameters were initially used: temperature, turbidity, pH, conductivity, TP, TDP, TN, TDN, Na, Cl, SO_4_^−2^, NO_3_^−^, and Fe. Conductivity and Cl were omitted from the final PCA analysis, as they showed a very high correlation with sodium (Supplementary Table 5, Spearman, > 0.99). Based on the rotation scores (of principal component (PC) 1 and PC2), we also excluded the following variables: TDP, TN, TDN (Supplementary Fig. 2 and Supplementary Table 4). Transformed (log + 1) *Legionella* spp. abundances at each station were included in the PCA plots.

We initially attempted to perform a multiple regression analysis and LMM analyses with *Legionella* as the dependent variable and all tested environmental parameters as the explanatory variables. However, due to multi-collinearity, this was not possible. Thus, we utilized a different approach to minimize the number of selected environmental parameters to include in the analysis. Based on the loading scores obtained from the final PCA analysis and considering collinearity (Supplementary Tables 6 and 7), we constructed a list of main chemical parameters, contributing to the main differences observed between the spring clusters (i.e., temperature, sodium, Fe, and pH).

To test their possible link to *Legionella* spp. abundance in water and biofilm samples, we fitted LMM models for both environments. We combined fixed and random effects, based on a selection procedure described by Zuur et al. (2009) and others [50–52]. For a full description of the LMM model selection and fitting, see Supplementary information (Supplementary methods section).

The final fitted model for water (*n* = 69) included sodium and temperature as fixed effects, and sampling station as a random effect with random slope only, altering by sodium. The model corresponds to the following final fitted equation:

$${Y}_{si} = {\beta }_{0}+({\beta }_{1}+{S}_{1s}){X}_{1i}+{\beta }_{2}{X}_{2i}+{e}_{si}$$

Where *Y*_*si*_ is *Legionella* spp. abundance., *s* = 1,…,S, indicates station, *i* = 1,…,I_s_ indicates measurement. *X*_*1*_ and *X*_*2*_ are the explanatory variables sodium and temperature, respectively (log center-scale values). *β* coefficients are the fixed effects and *S*_*1s*_ is the random effect (random slope) for sampling station. *e*_*si*_ indicates errors are normally distributed with mean 0 and variance *σ*2 [~ *N*(0, *σ*2)].

The final fitted model for biofilm (*n* = 49) included sodium as a fixed effect, with sampling station and collection date as crossed random effects (random intercepts only), corresponding to the following final fitted equation:

$${Y}_{si}={\beta }_{0}+{S}_{0s}+{D}_{0i}+{\beta }_{1}{X}_{i}+{e}_{si}$$

Where all are as for water, with the following distinctions: The single fixed factor *X* is the explanatory variable Na.* S*_*0s*_ and* D*_*0i*_ are the random effect sampling station and collection date, respectively.

*t* statistics, *p-*values, and conditional R^2^ are presented in the text.

### Next-generation sequencing (NGS)

To explore *Legionella* spp. microbial community, we utilized NGS amplicon sequencing, performed on water (*n* = 40) and biofilm (*n* = 25) samples, representative of each spring cluster: THS (*n* = 20), Haon borehole (*n* = 4), Tabgha (*n* = 33), and Fuliya (*n* = 8). Selected stations from the following dates were sent for NGS sequencing: September 2017, January 2018, June 2018, October 2018 and January 2019. 16S rRNA gene amplicon sequencing of the V3-V4 variable regions was done using specific *Legionella* spp. primers: 5′- GGCCTACCAAGGCGACGATCG-3′ (Lgsp17F, forward) and 5′- CACCGGAAATTCCACTACCCTCTC-3′ (Lgsp28R, reverse), as previously described [53], and universal primers 5′-CCTACGGGNGGCWGCAG-3′ (341F forward) and 5′-GACTACHVGGGTATCTAATCC-3′ (805R reverse), both sequenced on an Illumina MiSeq V3 platform, yielding 2 × 300 bp paired-end reads. To examine potential *Legionella* hosts, NGS amplicon sequencing designed for protozoa [54] was performed on the 18S rRNA gene targeting the V9 region utilizing primers: 5′-GTACACACCGCCCGTC-3′ (EUK1391F, forward) and 5′-CCTTCYGCAGGTTCACCTAC-3′ (EUK1510R, reverse), on an Illumina MiniSeq platform, yielding 2 × 153 paired-end reads following the manufacturer’s guidelines. Library preparation and sequencing of the September 2017 16S DNA extracts were performed at the MR DNA sequencing center (www.mrdnalab.com, Shallowater, TX, USA). Sequencing was done with barcodes on the forward primer, in a 30-cycle PCR reaction using the HotStarTaq Plus Master Mix Kit (Qiagen, USA). PCR conditions for the *Legionella*-specific primers were 94 °C for 3 min, followed by 30 cycles of 94 °C for 30 s, 68 °C for 40 s and 72 °C for 1 min and a final elongation step at 72 °C for 5 min. For the 16S universal primers, the same conditions were used, with annealing temperature of 53 °C. PCR products were visualized on a 2% agarose gel to determine bands relative intensity. Pooled samples were purified using calibrated Ampure XP beads. Then the pooled and purified PCR products were used to prepare an Illumina DNA library. Library preparation and sequencing for the remaining DNA extracts (16S and 18S) were done at Research Resources Center (RRC), University of Illinois Chicago (UIC), utilizing a two-stage amplicon sequencing [55]. For both primer sets, a first PCR reaction was performed, using the MyTaqHS 2X master mix in 10 μl reactions, with primers containing a 5′ linker at a final concentration of 500 nM each. PCR conditions for the *Legionella*-specific primers were 95 °C for 5 min, followed by 28 cycles of 95 °C for 30 s, 68 °C for 45 s, 68 °C for 30 s, and final elongation at 68 °C for 7 min. For the 16S universal primers and 18S rRNA primers, the same conditions were used, with annealing temperatures of 53 and 57 °C, respectively. All obtained templates were purified, quantified, and used in a second PCR reaction, to incorporate barcodes and sequencing adapters, with a final primer concentration of 400 nM. Cycling conditions were 95 °C for 5 min, followed by 8 cycles of 95 °C for 30 s, 60 °C for 30 s, 68 °C for 30 s, and final elongation at 68 °C for 7 min. Final PCR amplicons were pooled in equal volume and purified using solid phase reversible immobilization (SPRI) cleanup, using AMPure XP beads. Raw sequence data were analyzed as previously described [56]. First, MacQIIME 1.9.1 [57] was used to achieve proper read orientation of sequence reads obtained from MR DNA sequencing center, using qiime-extract.barcodes.py. The raw sequences were sorted based on orientation and barcodes were extracted. Raw sequence data from both centers were then processed using QIIME2 2020.11 [58]. Data was demultiplexed and quality filtered using the q2-demux plugin. Denoising was performed with DADA2 [59], using the q2-dada2 plugin. We examined amplicon sequence variants (ASVs) and not operational taxonomic units (OTUs), as it enables distinguishing single-nucleotide variations [60]. To account for length variations, ASVs were defined by clustering at 100% similarity [61]. For each primer set, all independent runs were merged post-denoising, ASVs occurring under a total frequency of 20 reads in less than 2 samples were removed. Taxonomy analysis was done with the 138-SILVA database QIIME release (at 99% clustering) [62], and the classifier was generated using the q2-feature-classifier plugin [63], using the extract-reads and fit-classifier-naive-bayes methods. The classifier generated was then used for the classification of the ASVs using the classify-sklearn method (ver. 0.23.1) [64]. To study the microbial populations of *Legionella* spp., we filtered the feature-frequency tables to retain 16S ASVs classified to *Legionella* spp. only (Supplementary Data 1). ASVs are denoted by unique MD5 hash codes generated by the DADA2 algorithm. To enable identification of each ASV and for reproducibility purposes, ASVs are present by the last 5 letters of the MD5 hash codes. The full code and ASV sequence are presented in Supplementary Data 1. As SILVA classification was largely successful up to the genus level, *Legionella* ASVs were left unclassified for most analyses. Phylogenetic analysis was performed with reference *Legionella* sequences (see “Phylogenetic analysis” section below). Potential *Legionella* spp. hosts were assessed by filtering the 18S ASVs to retain only those classified as *Amoebozoa*, *Ciliophora* (Ciliates), and *Percolozoa* (Excavata) (Supplementary Data 2). All analyses were performed on these filtered tables. Alpha (Shannon, Faith’s PD, Pielou’s evenness, and observed ASV indices) and beta (Jaccard similarity coefficient and Bray–Curtis dissimilarity indices) diversity analyses were assessed at rarefying depths of 1222 and 986 reads per sample for the 16S and 18S rRNA genes, respectively (using the core-metrics-phylogenetic function of the q2-diversity plugin [65]). Beta diversity was visualized using the q2-emperor plugin. Rarefaction curves were generated at varying depths to ensure ASV identification reached a plateau and to justify the rarifying depth chosen for the diversity analysis (using the alpha-rarefaction function of the QIIME2-diversity plugin, Supplementary Fig. 5). Statistical analysis for all indices was done using the alpha-group-significance (Kruskal–Wallis tests, *p*-values adjusted according to the Benjamini–Hochberg FDR correction) and beta-group-significance (PERMANOVA, PERMDISP and ADONIS with 999 permutations, *p*-values adjusted according to the Benjamini–Hochberg FDR correction). We assessed alpha and beta diversity between the two sequencing centers, to ensure differences were not substantial (Supplementary Tables 11, 12, 17, and 18). Slight differences were noted in the beta indices, yet these were small and may have originated in a small variation between the years (Supplementary Fig. 6). Correlations between the beta metrics of 16S and 18S rRNA genes was performed by Mantel test (*n* = 48) with 999 permutations (using q2-diversity-mantel plugin). Focusing on more abundant ASVs, we further filtered the feature-frequency tables to exclude ASVs occurring under a total frequency of 2000 reads for *Legionella* and 500 reads for potential *Legionella* hosts, in a minimum of 2 samples. Un-rarefied frequency filtered tables of absolute counts, and matching tables converted to relative abundance (using the relative-frequency function of the q2-feature table plugin), were exported in biome format and used for downstream statistical analysis and visualization in R, using the following packages: Phyloseq [66], ggplot2 [67], VennDiagram [68], and Complexheatmap [69]. Separate ASV Heatmaps were constructed for water (*n* = 40) and biofilm (*n* = 25) samples (row) and ordered according to descending sodium concentrations. ASVs (column) were grouped by hierarchical clustering, using hclust with the “average” clustering method.

### Phylogenetic analysis

Phylogenetic trees were constructed for *Legionella* spp. ASVs obtained from the NGS amplicon sequencing, together with previously characterized reference *Legionella* sequences denoted with the corresponding NCBI accession numbers (Supplementary Data 3). Separate phylogenetic trees were created for water (*n* = 38) and biofilm (*n* = 26) samples. Trees presenting all spring clusters, were constructed using data filtered to a minimal total frequency of 0.5% (water = 4714 reads and biofilm = 2500 reads) of all reads, in a minimum of 2 samples. The reference sequences were manually trimmed to match the length of the ASVs. Evolutionary analyses were conducted in MEGA11 version 11.0.9 [70], alignment of all ASVs was carried out using the imbedded muscle algorithm [71]. The evolutionary history for all trees was inferred using the Maximum-Likelihood method and Hasegawa-Kishino-Yano (HKY) [72] model. *Coxiella burnetii* was included as an outgroup. Initial trees for the heuristic search were obtained automatically by applying Neighbor-Join and BioNJ algorithms to a matrix of pairwise distances estimated using the maximum composite likelihood (MCL) approach, and then selecting the topology with superior log likelihood value. Bootstrap values (1000 replicates) above 50% are denoted by a black dot next to the branch. Trees are drawn to scale, with branch lengths indicating the number of substitutions per site. Trees were visualized using the R package ggtree [73]

### Statistical analysis

Statistical analyses were performed using the R (v. 4.0.3) and QIIME2 (v. 2020.11) software. qPCR statistical analysis was performed by Kruskal–Wallis and post hoc via Wilcox tests, using the rstatix package (v. 0.7.0). Box plots show median and interquartile ranges. The LMM model selection is based on the procedure described by Zuur et al. and others [50–52], and is extensively elaborated in the “Characterization of spring cluster physico-chemical properties and linear mixed model effect (LMM) with *Legionella* spp. abundance” section. Statistical analysis for all microbiome diversity indices was done using the alpha-group-significance (Kruskal–Wallis tests) and beta-group-significance (PERMANOVA, PERMDISP, and ADONIS with 999 permutations). *p*-values were adjusted according to the Benjamini–Hochberg FDR correction. All statistical tests were two-sided.

### Random forest analysis

To study which *Legionella* ASVs are distinctive of high sodium concentrations, we employed the random forest machine learning algorithm, to classify ASV relative abundance to spring clusters and regress against sodium levels, using packages caret [74] and randomForest [75]. As Tabgha and Fuliya stations showed similar sodium concentrations, ASVs of the two spring clusters were grouped prior to the analysis. Relative abundance ASV tables of the 16S (minimum 20 reads, in a minimum of 2 samples) gene for water (*n* = 40) and biofilm (*n* = 25) sample were analyzed separately, and first filtered to remove ASVs with relative abundance less than 0.001. The ASV tables were then centered and scaled, to reduce the influence of interindividual variation, prior to model training. The random forest models (*n*_trees_ = 1001) were run with the mtry parameter set to default and error was measured as out-of-bag (OOB) error. ASV importance was measured for the regression procedure, using the decrease in mean squared error (%IncMSE). Permutation tests were used to evaluate model significance and leave-one-out-cross-validation (LOOCV) to estimate accuracy.

### Cross-domain co-occurrence networks

To study the associations between *Legionella* ASVs (characteristic of high sodium concentrations), and their potential protozoan hosts, we investigated the cross-domain co-occurrence patterns, based on the described above 16S and 18S rRNA NGS amplicon sequencing data. Raw data were pre-filtered to retain a minimal total frequency of 20 reads, in a minimum of 2 samples. Different network analysis approaches have been proposed and developed over the years, yet all encounter significant computation challenges. Among the difficulties are the compositional nature of microbiome data sets; sparsity of ASVs, with high proportion of the zero counts; highly complex microbial communities, consisting of a very large number of ASVs and millions of potential interactions; diverse potential patterns of interaction (e.g., linear, exponential, periodic); heterogeneity in distribution between samples and geographic collection sites and ASVs interaction with environmental factors [76–78]. To add to that, analysis of cross-domain interactions mandates by its very nature added complexity with the combination of two different datasets. To address these challenges, we first reduced the number of ASVs, by focusing on sodium-rich environments. We also filtered the 18S data to only contain known potential protozoan hosts (i.e., *Ciliophora*, *Amoebozoa* and *Percolozoa*). We employed a random forest algorithm to focus on *Legionella* ASVs indicative of these ecosystems. To cope with these issues, we included in the analysis ASVs from Haon borehole (*n* = 4) and Tiberias Hot Springs (THS, *n* = 20) stations only. Furthermore, we included in the analysis *Legionella* ASVs present in the top 30 ASVs importance table, obtained from the random forest procedure. Correlation networks were constructed via the SPIEC-EASI [79] R package version 1.1.2, as described by Tipton et al. [80] and https://github.com/zdk123/SpiecEasi. Normalization was performed internally within the spiec.easi function, by first converting the data to relative abundance followed by center-log scaling. We utilized the sparse graphical lasso (glasso), with optimal sparsity parameter based on the Stability Approach to Regularization Selection (StARS), threshold set to 0.1. Networks were analyzed using functions of the R package igraph [81] version 1.2.6. Edges with low weights (> 0.01) and unconnected nodes were removed and only positive associations between ASV nodes are presented. Edge width corresponds to association strength between ASVs.

## Results

### *Legionella* species are more abundant in spring clusters with high sodium concentrations

**Fig. 2:**
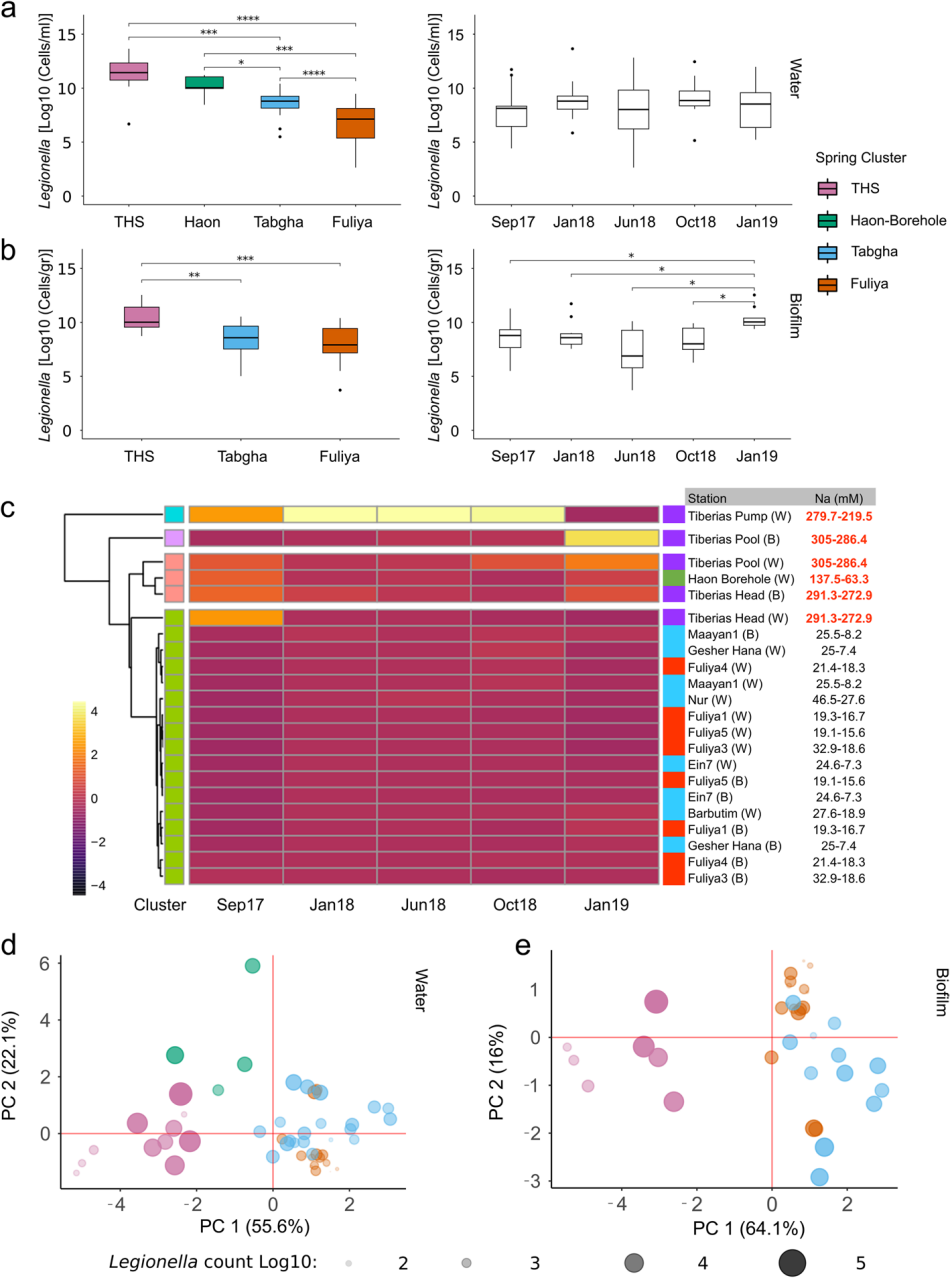
Absolute abundance of *Legionella sp.* follows sodium concentrations. Quantification by qPCR was done using primers and probes specific to *Legionella* spp., targeting the 16S rRNA gene. Abundance (Log10 transformed) was analyzed in **a** water (*n* = 64) and **b** biofilm (*n* = 50) samples, according to spring cluster (left panels) and collection date (right panels). Kruskal–Wallis tests and post hoc via Wilcox with BH corrections are presented in Supplementary Table 9. **c** Heatmap of *Legionella* sp. abundance. Samples (rows, *n* = 22) for each collection date (column, *n* = 5) were grouped by Hierarchical *k*-means clustering (Supplementary Fig. 4 and Supplementary Table 8). Sodium concentrations (ranges, in mM) were subsequently added to each sample (red color denotes high sodium concentrations). The heatmap color scale represents high (yellow to red) and low (purple to black) values. Hierarchical *k*-means clusters are represented by randomly assigned colors to the left of the heatmap. Spring cluster colors are presented to the right of the heatmap. **d + e** Principal component analysis (PCA) of selected physico-chemical parameters measured at each sampling site, representing the four spring clusters. PCA was performed on **d** water (*n* = 55) and **e** biofilm (*n* = 39) samples, obtained between January 2018 and January 2019 and included the following environmental variables: Temperature, Ph, Na, Turbidity, TP, SO_4_, NO_3_^−^, and Fe. Size and transparency represent the log + 1 values of qPCR *Legionella* spp. levels. Loading scores and vectors are presented in Supplementary Table 6 and Supplementary Fig. 3. The environmental variables were chosen following a PCA analysis from a larger subset of environmental factor (see Supplementary Fig. 2 and Supplementary Tables 4 and 5). THS = Tiberias Hot Springs. Significance level; * *p* ≤ 0.05; ** *p* ≤ 0.01; *** *p* ≤ 0.001; **** *p* ≤ 0.0001

*Legionella* absolute abundance was determined using qPCR on DNA extracted from samples taken from springs of varying salinity. The primers used were designed to detect sequences specific to the 16S rRNA gene of members of the genus *Legionella* [46]. Surprisingly, the highest absolute abundance of *Legionella* was found at the most saline environments of THS and Haon borehole springs, in both water (Kruskal–Wallis, H = 34.91, *P* = *1.27e − 07*) and biofilm (Kruskal–Wallis, H = 12.68, *P* = *0.0017*) samples (Fig. 2a,b; left panels). All sampling sites of both water (14 of 14) and biofilm (10 out of 10) were positive for *Legionella*. Measured cell abundances were in the range of 10^1^–10^4^ (Fuliya), 2 × 10^2^–3 × 10^4^ (Tabgha), 7 × 10^2^–8 × 10^5^ (THS) and 4 × 10^3^–7 × 10^4^ (Haon borehole) for water samples (cells/ml), and 3 × 10^2^–3 × 10^4^ (Fuliya), 10^2^–3 × 10^4^ (Tabgha) and 6 × 10^3^–2 × 10^5^ for biofilm samples (cells/g). Only a weak, yet significant, correlation was noted between the *Legionella* abundance found in water vs. biofilm environments (Spearman, *r* = 0.31, *P* = 0.028) (Supplementary Fig. 7). *Legionella* abundance did not seem to vary based on seasons, and the only significant difference in collection dates was observed in biofilm collected in January 2019 with higher *Legionella* abundance compared to all other dates (Kruskal–Wallis, H = 14.68, *P* = *0.005*; Fig. 2a,b, right panels). All Kruskal–Wallis and post hoc via Wilcox tests with BH corrections are presented in Supplementary Table 9. Hierarchical *k*-means clustering reveals that *Legionella* abundance can largely be grouped according to sodium concentrations. As can be seen in the scaled heatmap, higher abundance was characteristic of higher sodium levels (Fig. 2c, Supplementary Fig. 4 and Supplementary Table 8).

### Saline spring cluster according to physico-chemical characteristics and mirror *Legionella* spp. abundance

The observed differences in *Legionella* spp. abundance corresponded to the variations in sodium concentrations, which differed substantially between the spring clusters. As differences were also noted in ranges of other physico-chemical characteristics (Supplementary Tables 1 and 2), we characterized these differences in a PCA plot, together with *Legionella* spp. abundance. Our analysis indicated that water samples of THS and Haon borehole stations each cluster independently, while Tabgha and Fuliya stations cluster together (PC1-PC2: 55.6–22.1%, Fig. 2d). Similarly, water analysis related to biofilm samples showed a similar pattern for samples taken from THS, Tabgha, and Fuliya (PC1-PC2: 64.1–16%, Fig. 2e). These results mirror *Legionella* spp. abundance and may indicate a link between the measured physico-chemical parameters and *Legionella* abundance.

Based on PC1-PC2 loading vectors/scores, the main separation for both water and biofilm samples was mainly driven by sodium, temperature, SO_4_^−2^, NO_3_^−^, pH, and Fe, (Supplementary Fig. 3 and Supplementary Table 6). Ranges of these physico-chemical parameters at the level of spring cluster are summarized in Supplementary Table 3. THS cluster was characterized mainly by very high temperatures, sodium and SO_4_^−2^ levels with slightly acidic pH conditions, and very low NO_3_^−^ levels. Fe levels fluctuated significantly between stations, with very high levels measured at Tiberias-Head station. Tiberias-Head station measured temperatures were particularly high throughout the study, ranging between 56 and 59 °C. Haon borehole was also characterized by relatively high (yet lower) sodium, Fe, and SO_4_^−2^ levels. Temperature and NO_3_^−^ were lower, with neutral to slightly basic pH conditions. The latter three were on the same scale as those measured at Tabgha and Fuliya. The Physico-chemical characteristics of the latter two were similar with lower sodium, Fe, and SO_4_^−2^ and higher NO_3_^−^ levels. Measured NO_3_^−^ levels were significantly lower at the Barbutim station, compared to other Tabgha stations.

### Sodium concentrations are strongly linked to *Legionella* abundance

To study the possible link between these chemical parameters and *Legionella* abundance, we constructed separate LMM models for water and biofilm samples. Allowing sodium to have a different effect on each water station (i.e., random slope), with sampling station as a random effect, the LMM models indicated the Log values of sodium had a strong effect on *Legionella* spp. abundance (LMM, *t* = 3.025, *P* = *0.007*). Temperature had only a weak effect (LMM, *t* =  − 2.062, *P* = *0.048*). In the biofilm samples, the model accounted for inter-variability related to sampling station and collection date (crossed, random intercept). Only sodium was included as a fixed factor in the final model and had a strong effect on *Legionella* spp. abundance (LMM, *t* = 4.039, *P* = *0.003*). The LMM models of both water and biofilm samples explained a relatively high percentage of variability in *Legionella* spp. abundance (R^2^ = 0.49 and R^2^ = 0.6, respectively), considering the final number of variables included.

High abundance of *Legionella* spp. in an environment of extremely high temperature and sodium concentrations, as exemplified in Tiberias-Head station, is surprising and counterintuitive. It has been reported that the combination of high temperatures and NaCl concentrations do not favor *L. pneumophila* growth [29, 82]. Yet, these investigations were conducted on laboratory strains of *L. pneumophila* (serogroup 1 Suzuki clinical isolate and serogroup 3 isolated from hospital tap water) and do not represent natural diversity.

We therefore examined *Legionella* community composition of the different spring clusters to test whether specific *Legionella* species are characteristic of these high salinity environments.

### *Legionella* microbial community

To characterize *Legionella* community composition, representative samples from the four spring clusters (THS; *n* = 20, Haon borehole; *n* = 4, Tangha; *n* = 33 and Fuliya; *n* = 8) were analyzed using NGS amplicon sequencing of the 16S rRNA gene, using *Legionella*-specific primers as well as universal primers. When using the universal bacterial 16S primer set, *Legionella* ASVs were present in only 21 out of 69 samples, with relative abundances ranging between 0.02 and 0.45%. In contrast, the *Legionella*-specific primers yielded 2475 ASVs with a total frequency of 3,402,219, after quality filtering, and exclusion of rare sequences. Of these, 1073 with a total frequency of 1,439,806 reads were specific to *Legionella* spp. (43%, Supplementary Table 10). *Legionella* ASVs were identified in 62 out of 69 samples, in all spring clusters. Read and ASV counts were both higher in water samples (1037, *n* = 40, 943,856 reads) compared to the biofilm samples (493, *n* = 26, 495,950 reads). Overall, the vast majority of ASVs present in biofilm samples (457 Out of 493) were also identified in the water samples (Supplementary Fig. 9). Although alpha diversity did not differ between water and biofilm samples (Supplementary Fig. 8a and Supplementary Table 13), beta diversity differed significantly for both the Jaccard index (PERMANOVA, F = 1.6, *P* = 0.02) and the Bray–Curtis index (PERMANOVA, F = 1.63, *P* = 0.015, Supplementary Fig. 8b and Supplementary Table 12).

### *Legionella* community composition is characterized by few, dominant ASVs in environments with high sodium concentrations

**Fig. 3:**
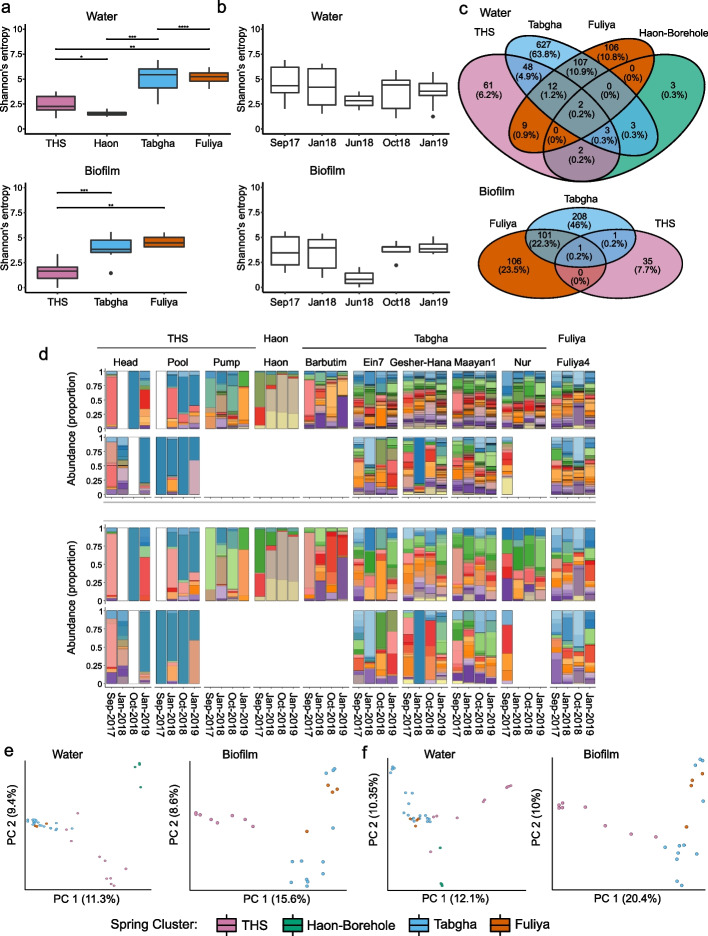
*Legionella* spp. community structure and composition shows an inverse relation to sodium concentration. Next-generation sequencing (NGS) was performed using *Legionella* spp.-specific 16S primers. Water (*n* = 40) and biofilm (*n* = 25) samples were analyzed separately. Spring clusters: Tiberias Hot Springs (*n* = 20), Haon borehole (*n* = 4), Fuliya (*n* = 8), and Tabgha (*n* = 33). **a + b** Alpha diversity of *Legionella* spp. across **a** spring clusters and **b** collection dates. Shannon’s entropy is presented for water and biofilm samples. Faith’s PD, Pielou’s evenness, and observed ASVs are presented in Supplementary Fig. 10a and b. Kruskal–Wallis test results and post hoc via Wilcox tests are presented in Supplementary Table 13. **c** Venn diagram of *Legionella* ASVs (minimum of 20 reads) in water and biofilm samples, according to spring cluster. **d** Relative abundance of *Legionella* ASVs, according to spring cluster and collection dates. *Legionella* ASVs were pre-filtered to a minimum frequency of 20 (top panel) or 2000 (bottom panel) reads. Samples are ordered by date, faceted vertically by spring cluster and horizontally by water and biofilm. As numerous ASVs (1073) were included in the construction of each barplot, colors are repetitive and are not indicative of specific ASVs. The identity of the most abundant ASVs is presented in Fig. 4a,b and Supplementary Data 4. White barplots indicate *Legionella* ASVs were not detected. Biofilm sampling was not possible for Tiberias Pump, Haon borehole, and Barbutim stations. For Nur station, only biofilm from September 2017 was available. **e + f** Beta diversity of *Legionella spp.* microbial population across spring clusters. Jaccard (**e**) and Bray–Curtis (**f**) are presented for water and biofilm samples. Beta diversity statistical significance was tested via PERMANOVA and PERMDISP analyses (Supplementary Tables 14 and 15). THS = Tiberias Hot Springs. Significance level; * *p* ≤ 0.05; ** *p* ≤ 0.01; *** *p* ≤ 0.001, **** *p* ≤ 0.0001

Contrary to *Legionella* abundance, alpha diversity of water and biofilm samples was significantly higher for Tabgha and Fuliya, as compared to THS and Haon borehole: Shannon entropy (Kruskal–Wallis, H = 23.12, *P* = 3.81e − 05; and H = 13.8, *P* = 9.6e − 04, respectively), Faith’s PD (Kruskal–Wallis, H = 23.8, *P* = 2.75e − 05; and H = 15.9, *P* = 3.53e-05, respectively) and observed ASVs (Kruskal–Wallis, H = 23.11, *P* = 3.83e − 05; and H = 14.67, *P* = 6.51e − 05, respectively). Pielou’s evenness was also significantly lower for THS biofilm stations (Kruskal–Wallis, H = 6.8, *P* = 0.033, Fig. 3a, Supplementary Fig. 10a and Supplementary Table 13). No significant differences in alpha diversity were observed between collection dates (Fig. 3b and Supplementary Fig. 10b). While THS biofilm stations were characterized by a high proportion of unique *Legionella* ASVs (35 out of 37), Tabgha and Fuliya ASVs were more frequently shared, in both water and biofilm samples (Fig. 3c). Interestingly, THS and Haon borehole presented relatively few dominant ASVs (Fig. 3d and Supplementary Data 4), several of which were especially prevalent (> 50%) (THS: be50d, 49fe1, a42cd, f01d3, 88adc; Haon borehole: 30a35, ea057). Notably, ASVs 849c0, c284e, and 84be2 were identified as *L. pneumophila* (Blast search: 100% identity) and found in all spring clusters (Supplementary Data 1). ASV e5746 (closest BLAST search: 94.93% identity with an uncultured *Legionella*) was extremely prevalent only in Tabgha and Fuliya spring clusters, in approximately 80% of the samples. We did not find ASVs common to most samples, across all spring clusters. Beta diversity analysis showed grouping of the samples according to spring clusters for both Jaccard (PERMANOVA, water: F = 3.39, *P* = 0.001; biofilm: F = 2.81, *P* = 0.001) and Bray–Curtis (PERMANOVA, water: F = 3.26, *P* = 0.001; biofilm: F = 3.3, *P* = 0.001), with Tabgha and Fuliya stations grouped more closely together compared to THS and Haon borehole (Fig. 3e,f and Supplementary Tables 14 and 15).

### Sodium levels are a major driver of *Legionella* diversity and community composition

To examine the effect of environmental conditions on beta diversity, we performed an ADONIS test (Supplementary Table 16) with the variables included in the LMM analysis, as performed on *Legionella* abundance (i.e., temperature, sodium, Fe, and pH). Correspondingly, we found sodium levels were the main factor explaining the variations in both Jaccard and Bray–Curtis indices (9.5% and 10.5% in water; 16.4% and 21.5% in biofilm samples, *P* = 0.001). Temperature and Fe levels were also significant in water samples but contributed less to the explained variance. In biofilm samples, only sodium levels were significant.

**Fig. 4:**
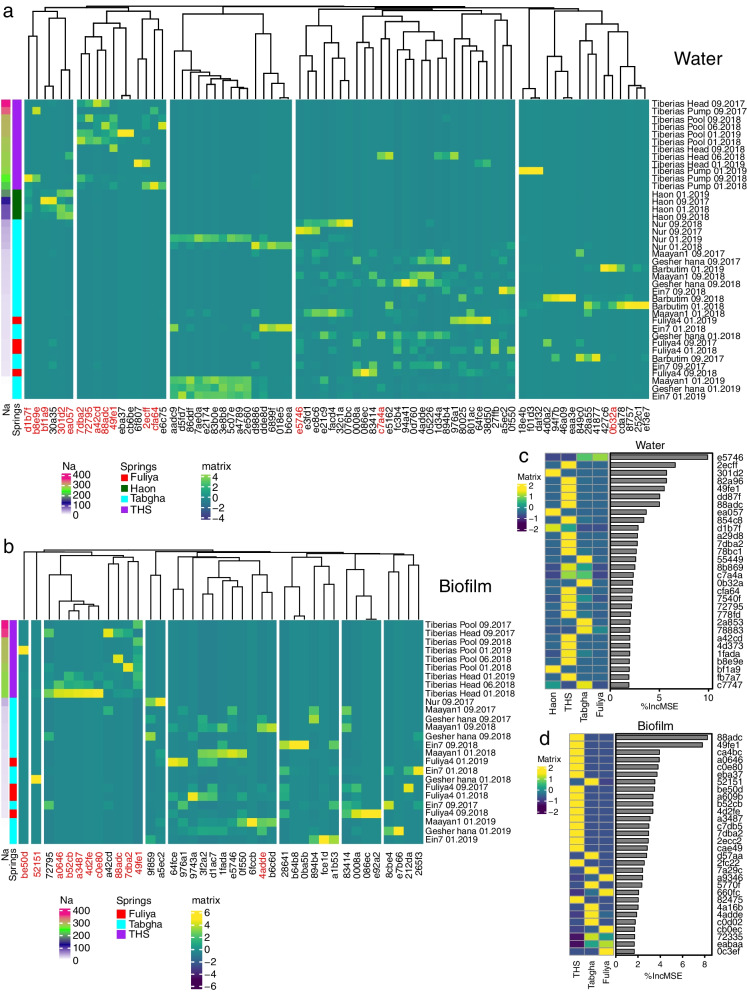
*Legionella* ASVs group according to sodium concentrations. Next-generation sequencing (NGS) was performed using *Legionella*-specific 16S primers. Raw data were pre-filtered to retain *Legionella* amplicon sequence variants (ASVs), present at a minimum of 20 or 2000 reads across all samples, in a minimum of 2 samples. Water (*n* = 40) and biofilm (*n* = 25) samples were analyzed separately. Spring clusters: Tiberias Hot Springs (THS, *n* = 20), Haon borehole (*n* = 4), Fuliya (*n* = 8) and Tabgha (*n* = 33). ASVs are represented by the last 5 letter-number code (for full-length codes and corresponding representative sequence, see Supplementary Data 1. Heatmap presenting abundant *Legionella* ASVs (2000 reads), in **a** water and **b** biofilm samples. ASVs were grouped according to Hierarchical clustering and ordered according to sodium concentrations. The heatmap color scale represents high (green to yellow) and low (blue to purple) centered-scaled values of *Legionella* ASVs relative abundance. Sodium concentrations and spring clusters are shown to the left of the heatmap. Additionally, ASVs associated with high sodium concentrations were also predicted by a random forest procedure (see “Random forest analysis” section). ASVs that were identified in the heatmap as well as the random forest procedure are colored red. *Legionella* ASVs associated with high sodium concentrations in the random forest are presented for **c** water and **d** biofilm samples. ASV importance was predicted by the machine learning algorithm. Scaled-centered relative abundances (20 reads) were used to evaluate ASV importance, based on the decrease in mean accuracy (%IncMSE)

To further explore the effect of high sodium concentrations on *Legionella* spp. community composition, we focused on more abundant ASVs (minimum 2000 reads), and constructed water and biofilm heatmaps, with samples (rows) ordered in descending sodium concentrations and ASVs (columns) grouped by hierarchical clustering. In both water and biofilm samples, the ASVs were distinctly clustered according to sodium concentrations, with THS and Haon borehole spring clusters grouped independently, while Tabgha and Fuliya grouped together (Fig. 4a,b).

To identify ASVs indicative of higher sodium concentrations, we utilized a random forest machine learning algorithm on all ASVs (quality filter minimum 20 reads), with Tabgha and Fuliya ASVs grouped together. ASVs (relative abundances) were classified according to spring cluster and regressed against sodium concentrations. The random forest model of *Legionella* microbiome in water and biofilm samples (Supplementary Data 5), enabled spring cluster prediction with accuracy of 82.5% (kapa = 67%, OOB = 17.5%, *P* = 0.001) and 92.3% (kapa = 83%, OOB = 7.7%, *P* = 0.001), respectively. Microbiome regression allowed the prediction of sodium concentrations of water and biofilm samples with R^2^ = 50%, RMSE = 85.3 and R^2^ = 63.6%, RMSE = 82.2, respectively. Importance tables of the top 30 ASVs contributing to the regression were generated, using the decrease in mean squared error (%IncMSE). Most ASVs predicted by the random forest algorithm were related to THS and Haon borehole clusters (Fig. 4c,d). The majority of ASVs related to these two clusters, were also detected in the abundant ASV heatmap (Fig. 4a,b highlighted in red), for both water (12 out of 17 ASVs) and biofilm (10 out of 12 ASVs) samples. This strategy enabled us to identify abundant ASVs that are indicative of environments with high sodium concentrations, and particularly of high sodium–high temperature environments.

### Extreme aquatic environments featuring high sodium and high temperature host abundant novel *Legionella* genotypes

**Fig. 5:**
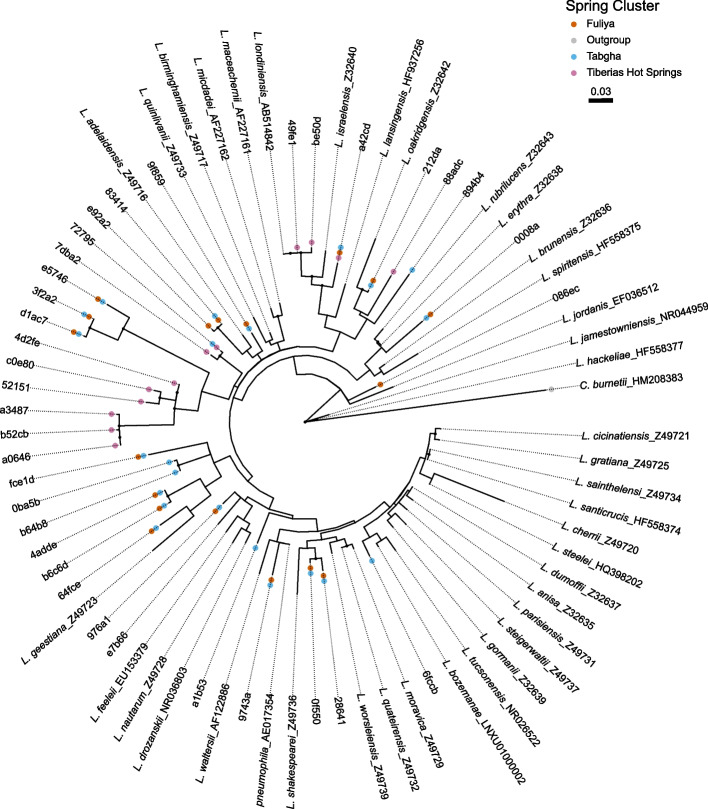
Phylogenetic analysis of *Legionella* ASVs. Phylogenetic tree was constructed alongside reference bacteria with corresponding NCBI accession numbers (Supplementary Data 4). Predominant ASVs (filtered for minimum 2500 reads (0.5%), in 2 samples) are presented for biofilm samples, from Tabgha, Fuliya, and Tiberias Hot Springs (THS) spring clusters. The evolutionary history of the tree was inferred using the Maximum-Likelihood method and Hasegawa-Kishino-Yano (HKY) model. *Coxiella burnetii* was included as an outgroup. The tree with the highest log likelihood is presented (− 3746.05). A discrete Gamma distribution was used to model evolutionary rate differences among sites (5 categories (+ G, parameter = 0.1868)). The rate variation model allowed for some sites to be evolutionarily invariable ([+ I], 34.30% sites). The tree is drawn to scale, with branch lengths measured in the number of substitutions per site. Branches with bootstrap values above 50% are demoted by a black dot. The analysis involved 75 nucleotide sequences. There were a total of 379 positions in the final dataset. Evolutionary analyses were conducted in MEGA11

Focusing only on the abundant ASVs, we performed a phylogenetic analysis, alongside reference *Legionella* species, across spring clusters presenting high and low sodium concentrations (minimum 0.5% reads, Fig. 5, Supplementary Fig. 12, and Supplementary Data 3). The Maximum-Likelihood trees generated for both water and biofilm samples were diverse, portraying unique ASVs, distinct from known *Legionella* species. Only a small number of the ASVs were found to be closely related to known *Legionella* species, e.g., *L. spiritensis* (ASV 301d2), *L. sainthelensi*, *L. santicrucis* (ASV ea057), and *L. londiniensis* (ASVs 49fe1, 2ecff, e6c75, and be50d).

Interestingly, 50% of the abundant *Legionella* ASVs associated with THS biofilm samples—representing high-sodium, high-temperature environments—grouped in distinct phylogenetic clades of the tree. *Legionella* ASVs related to Tabgha and Fuliya, which portray low sodium concentrations, grouped together in separate clades (Fig. 5). A similar phenomenon, yet less profound, was also observed in the water samples (Supplementary Fig. 12). These findings point to unique-abundant *Legionella* species that are able to flourish in high-sodium, high-temperature environments.

We attempted to isolate such *Legionella* species from THS, using the methods outlined in “Procedures for the Recovery of *Legionella* from the Environment” [83], yet all attempts were unsuccessful. We also attempted isolation by inoculation with the amoebae host *Acanthamoeba castellanii* as described by Campocasso et al. [22], again to no avail. Our inability to isolate *Legionella* strains from sodium-rich environments could stem from various factors, among them, a difference in the lifestyle of *Legionella* species found in these environments and their potential dependence on interaction with specific protozoan hosts.

### *Amoebozoa* and *Ciliophora* community composition shows trends similar to those seen in *Legionella* spp. community composition

**Fig. 6:**
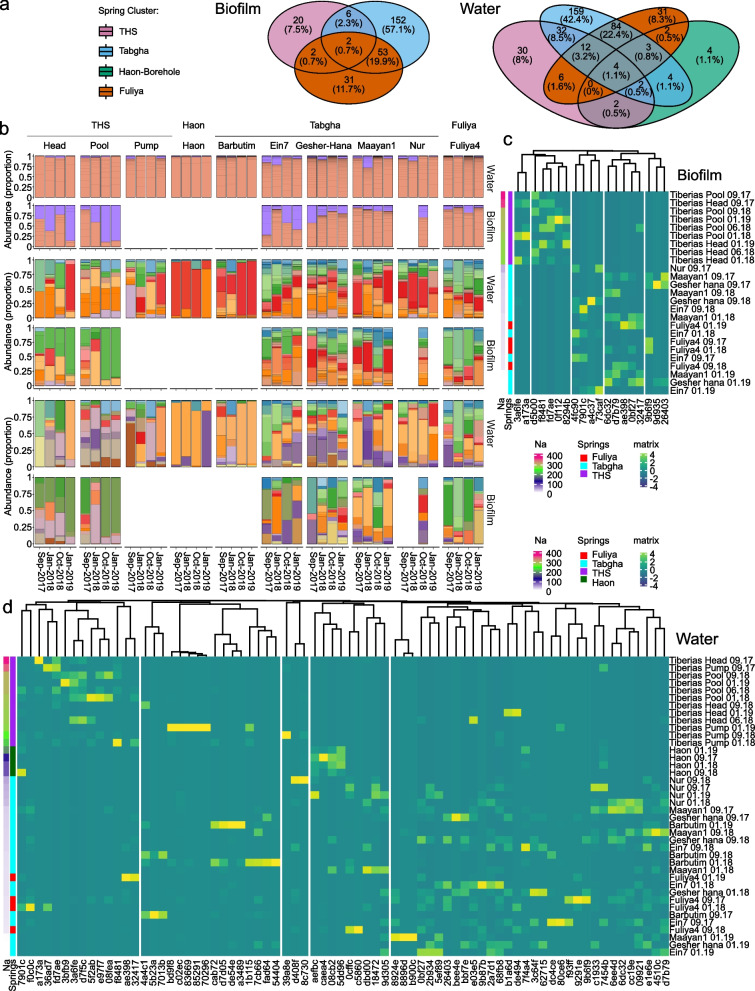
Community structure and composition of potential *Legionella* hosts resembles that of *Legionella* spp. Next-generation sequencing (NGS) was performed using universal protists primers, designed for the V9 region of the 18S rRNA gene. Raw data were pre-filtered to retain *Amoebozoa*, *Ciliophora* (Ciliates), and *Percolozoa* (Excavata) amplicon sequence variants (ASVs), present at a minimal frequency of 20 or 500 reads across all samples, in a minimum of 2 samples. Water (*n* = 40) and biofilm (*n* = 25) were analyzed separately. Spring clusters: Tiberias Hot Springs (*n* = 20), Haon borehole (*n* = 4), Fuliya (*n* = 8), and Tabgha (*n* = 33). ASVs are represented by the last 5 letters of a letter-number code. For the full-length code and corresponding sequence, see Supplementary Data 2. **a** Venn diagram presenting ASVs of potential *Legionella* hosts (minimum 20 reads) in water (right panel) and biofilm (left panel) samples, separated according to spring cluster. **b** Relative abundance of potential *Legionella* hosts ASVs, according to spring cluster and collection dates. ASVs at the phylum level (top panel, min 20 reads) and at the ASV level (middle and bottom panels, minimum 20 and 500 reads, respectfully) are presented. Samples are ordered by date, faceted vertically by spring cluster and horizontally by water and biofilm. As numerous ASVs (400) were included in the construction of each barplot, colors are repetitive and are not indicative of specific ASVs. The identity of the most abundant ASVs is presented in the heatmaps (Fig. 6c,d). For Nur station, only biofilm from Sep 2017 was available. **c + d** Heatmap presenting abundant ASVs of potential *Legionella* hosts (minimum 500 reads), in **c** biofilm and **d** water samples. ASVs were grouped according to Hierarchical clustering and ordered according to sodium concentrations. The heatmap color scale represents high (green to yellow) and low (blue to purple) values. Sodium concentrations and spring clusters are presented to the left of the heatmap

In laboratory conditions, *L. pneumophila* growth is inhibited by the combination of elevated sodium concentrations and temperatures [29], with mutants more resistant to high sodium concentrations tending to be less virulent [40]. The ability of *Legionella* spp. to enter and multiply in various hosts (i.e., *Amoebozoa*, *Ciliophora*, and *Percolozoa*) has been suggested to aid survival and persistence in saline environments [35, 84]. To study the occurrence of *Legionella* spp. with potential protozoan hosts, we utilized 18S rRNA gene amplicon sequencing with universal primers designed for protozoa. After quality filtering, trimming using DADA2 and the removal of rare sequences (minimum 20 reads, in 2 samples), a total of 6917 ASVs with a total frequency of 5,650,504 ASVs were obtained. Of these, 400 with a total frequency of 474,320 were of potential protozoan hosts (i.e., *Amoebozoa*, *Ciliophora*, and *Percolozoa*; Supplementary Data 2). In both water and biofilm samples, most ASVs were classified by the SILVA SSU 138-database, as *Ciliophora* (Fig. 6b; top panel). In biofilm samples of THS and Ein7-Tabgha stations, *Amoebozoa* were more abundant.

**Fig. 7:**
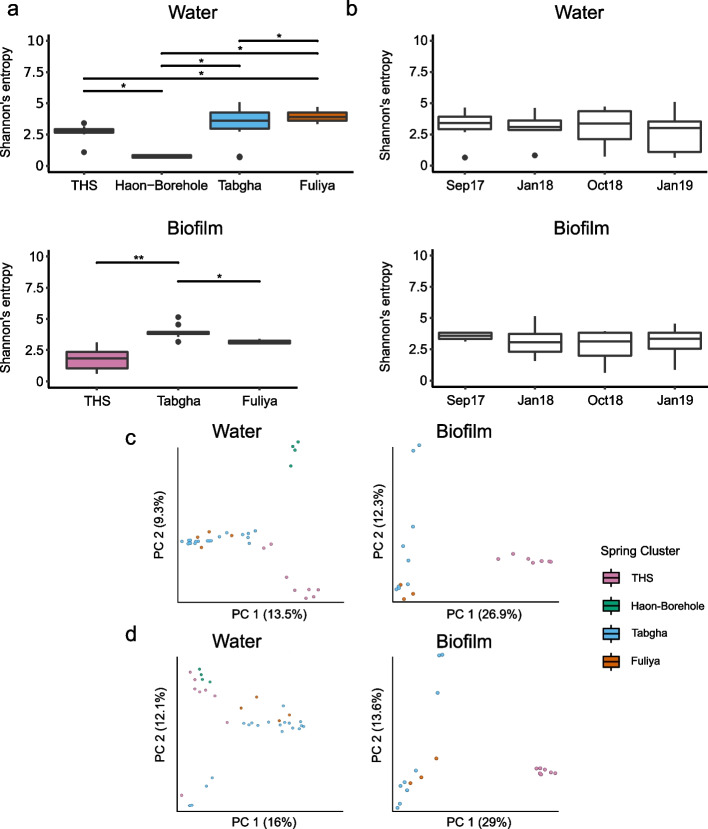
Alpha and beta diversity of potential *Legionella* hosts. Next-generation sequencing (NGS) was performed using universal primers, designed for the V9 region of the 18S rRNA gene. Raw data were pre-filtered to retain *Amoebozoa*, *Ciliophora* (Ciliates), and *Percolozoa* (Excavata) amplicon sequence variants (ASVs), present at a minimal frequency of 20 reads across all samples, in a minimum of 2 samples. Water (*n* = 40) and biofilm (*n* = 25) samples were analyzed separately. Alpha and beta diversity analyses were performed at a rarefaction depth of 986 reads. **a + b** Alpha diversity of potential *Legionella* hosts across (**a**) spring clusters and (**b**) collection dates. Shannon’s entropy is presented for water (left panels) and biofilm (right panels) samples. Faith’s PD, Pielou’s evenness, and observed ASVs are presented in Supplementary Fig. 11a and b. Kruskal–Wallis test results and post hoc via Wilcox tests are presented in Supplementary Table 19. **c + d** Beta diversity of potential *Legionella* hosts across spring clusters. **c** Jaccard and **d** Bray–Curtis are presented for water (left panels) and biofilm (right panels) samples. Beta diversity statistical significance was tested via PERMANOVA and PERMDISP analyses (Supplementary Tables 20 and 21)

The overall trends in the *Amoebozoa* and *Ciliophora* community structure and composition were reminiscent of those of the *Legionella* community structure, with higher alpha diversity in Tabgha and Fuliya stations (Fig. 6a, Supplementary Fig. 11a and Supplementary Table 19). No significant differences in diversity were noted based on the sample collection dates (Fig. 7b and Supplementary Fig. 11b). As seen in the *Legionella* ASVs, THS and Haon borehole-related ASVs were relatively few and dominant (Fig. 6a, b; middle and bottom panels). Beta diversity showed similar patterns (Fig. 7c,d and Supplementary Tables 20 and 21), with THS and Haon borehole stations grouped independently, and Tabgha-Fuliya stations grouped together: Jaccard (PERMANOVA, water: F = 3.28, *P* = 0.001; biofilm: F = 3.99, *P* = 0.001) and Bray–Curtis (PERMANOVA, water: F = 3.4, *P* = 0.001; biofilm: F = 4.18, *P* = 0.001). A two-sided mantel test was performed to measure the correlation between the beta matrices of *Legionella* spp. and potential *Legionella* hosts. Jaccard similarity coefficient and Bray–Curtis dissimilarity both exhibited a strong correlation (PERMANOVA, *r* = 0.76, *P* = 0.001; and *r* = 0.73, *P* = 0.001, respectively). Water and biofilm heatmaps generated for *Amoebozoa* and *Ciliophora* ASVs (minimum 500 reads, in 2 samples) were also grouped according to spring cluster sodium concentrations, as did *Legionella* spp. ASVs (Fig. 6c,d). These results suggest strong selection pressure related to high sodium concentrations, with similar effects on both related microbial communities.

### Co-occurrence of *Legionella* spp. with potential hosts

Our results indicate high *Legionella* abundance, with relatively few dominant ASVs in environments of elevated sodium concentration (THS and Haon borehole clusters) and elevated temperatures in the THS stations. These findings may be related to co-occurrence with potential *Legionella* hosts. To test this, we investigated the co-occurrence of *Legionella* spp. with potential hosts only in the saline environments. We constructed a cross-domain co-occurrence network, based on the results obtained from the 16S and 18S rRNA NGS amplicon sequencing, utilizing the SPIEC-EASI package for the THS and Haon borehole stations.

**Fig. 8:**
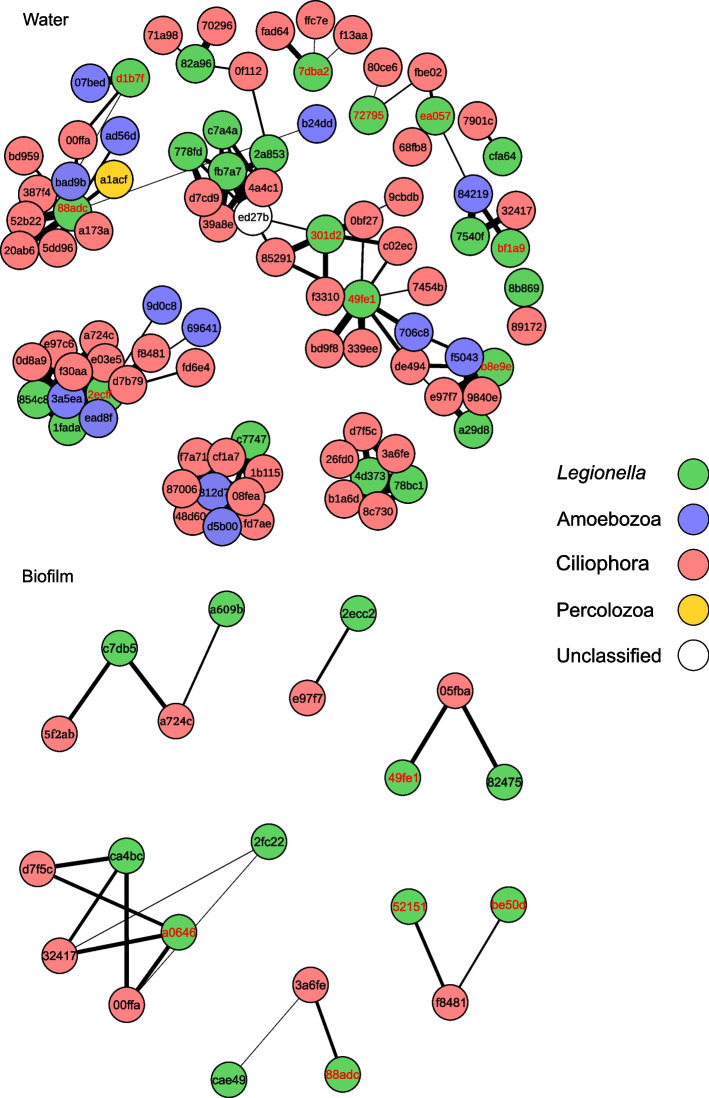
Co-occurrence of *Legionella* ASVs with protozoans *Ciliophora*, *Amoebozoa*, and *Percolozoa*. SPIEC-EASI cross-domain co-occurrence networks of *Legionella* ASVs and potential protozoan hosts, associated with high sodium concentrations, are presented for water and biofilm samples. Networks are based on next-generation sequencing (NGS) performed using *Legionella* spp.-specific 16S primers. Raw data were pre-filtered to retain *Legionella* amplicon sequence variants (ASVs). NGS amplicon sequencing targeting protozoa was performed using 18S rRNA primers directed to the V9 region. Raw data were pre-filtered to retain *Amoebozoa*, *Ciliophora*, and *Percolozoa* ASVs. We included ASVs present at a minimal frequency of 20 reads across all samples, in a minimum of 2 samples. To focus on *Legionella* spp. associated with high sodium concentrations, the analysis includes ASVs from Tiberias Hot Springs (THS, *n* = 20) and Haon borehole (*n* = 4) stations. Moreover, we only included *Legionella* ASVs present in the top 30 ASV importance table, of the random forest procedure (Fig. 4c,d). ASVs are represented by the last 5 letters of a letter-number code. For the full-length code and corresponding sequence, see Supplementary Data 1 and 2. Edges with low weights (> 0.01) and unconnected nodes were removed. Only positive associations between ASV nodes are presented. Edge width corresponds to association strength between ASVs

Our analysis indicated that the co-occurrence network in water samples was substantially more complex as compared to the biofilm samples (Fig. 8). The former included associations with *Ciliophora* (orders *Armophorea*, *Spirotrichea*, *Conthreep*, *Spirotrichea*, *Litostomatea*, and *Heterotrichea*), *Amoebozoa* (genera *Acanthamoeba, Dactylopodida, Discosea, Echinamoebida, Vannella, and Flabellula*), and one *Percolozoa* (*Pharyngomonas* sp.). While the protozoan community in water samples consisted primarily of *Ciliophora* (Fig. 6b; top panel), interactions with *Amoebozoa* were common. About 25% of the identified associations (20 out of 79) were of the Ciliates class *Oligohymenophorea* and 35% were of the *Amoebozoa* family *Vannellida* (4 out of 14). Interestingly, in the water samples, most *Legionella* ASVs identified by the random forest algorithm and the abundant ASV heatmap co-occurred with both *Ciliophora* and *Amoebozoa* (e.g., 49fe1, ea057, and 88adc). ASV 88adc was also associated with the Percolozoan *Pharyngomonas* sp. Surprisingly, although *Amoebozoa* were prevalent in the biofilm samples, associations were noted only with *Ciliophora* members (orders *Spirotrichea*, *Conthreep*, and *Spirotrichea*). Co-occurrence networks in Tabgha and Fuliya spring clusters were more complex as compared to THS and Haon borehole (Supplementary Fig. 13). As the number of 18S ASVs was much higher in these clusters, this result is expected. Similarly, THS biofilm samples showed a very low number of *Ciliophora* and in particular of Amoeba ASVs (Fig. 6b).

## Discussion

*Legionella* are considered to be predominantly freshwater organisms [34] that associate with and replicate within a wide range of protozoan hosts in the environment [8, 9]. Several reports suggest that *Legionella* can persist in seawater and other saline environments [20, 35, 38, 39, 85, 86]. Yet, studies related to *Legionella* occurrence and diversity in natural saline environments are scarce and our understanding related to the effect of salinity and grazing as co-occurring selection pressures is very limited. Our findings demonstrate that *Legionella* can flourish in sodium-rich ecosystems, with a small but dominant group of unique *Legionella* species, specific to these environments. Interestingly, we observed this phenomenon in combined conditions of high sodium and high temperature, which are considered unfavorable for *Legionella* growth in laboratory experiments [29, 39, 82, 87].

The study design consisted of spring clusters of varying sodium concentrations, grouped according to various characteristic physico-chemical parameters [42–44]. We performed PCA analysis and evaluated their ability to explain the variations in *Legionella* abundance, by constructing LMM models. Our results indicate sodium was the main explaining factor in both water and biofilm samples. Surprisingly, we found the highest *Legionella* abundance in sodium-rich environments. These included the THS cluster, which exhibited high temperatures, particularly at the Tiberias-Head station (56–59 °C, 273–291 mM). Reports on *Legionella* abundance in saline environments indicate that *L. pneumophila* are able to survive high sodium concentrations (supplemented with up to 3.5% NaCl) at temperatures ranging between 4 and 29.5 °C [29, 35, 37, 38]. While *Legionella* are frequently reported in environments with high temperatures, up to and above 60 °C [15, 19, 88–92], the combined effect of elevated temperatures and high sodium concentrations do not favor their growth in laboratory experiments. Most studies were conducted on *L. pneumophila* and included incubation in NaCl solutions or sterile seawater for several days at 37 °C. Under these conditions, growth was significantly inhibited [29, 87], and cells may be viable but nonculturable [82]. Even when isolated from seawater (serogroups 1, Allentown 82 strain), incubation at elevated temperatures (33 ± 2 °C) does not seem to favor growth [39]. Since the majority of studies examining *Legionella* response to temperature and salinity focus on *L. pneumophila*, the true diversity of the genus has been underestimated in this respect.

To gain a broader understanding of *Legionella* diversity, we utilized a next-generation amplicon-based sequencing approach using *Legionella* genus-specific primers [53]. As *Legionella* relative abundance is often low [93], this approach can help alleviate PCR-related biases [94, 95], which may lead to underestimated diversity. Indeed, employing universal primers, we found low *Legionella* relative abundance which significantly increased with the specific primers.

While environments of low sodium concentrations were characterized by numerous common *Legionella* ASVs, those with high sodium concentrations were characterized by few dominant ASVs. Moreover, sodium-rich environments that exhibit low versus high temperatures presented different ASVs (Figs. 3c and 4a,b), suggesting selection pressures in these two environments favor different groups of *Legionella* species. Several ASVs were shared by both environments, indicating they may have the ability to adapt to the conditions in both environments. We did not observe significant seasonal variations related to *Legionella* diversity. Investigations utilizing the *Legionella* primers used in our study show conflicting results as to the effect of season on *Legionella* microbial community composition and diversity [96, 97]. These results indicate that the seasonal variations may be affected by additional environmental conditions.

Most *Legionella* ASVs, from all environments, were not related to known *Legionella* species. Few exceptions were noted, with ASVs closely related to *L. londiniensis, L. spiritensis*, *L. santicrucis, L. sainthelensi,* and *L. pneumophila*. Similar findings have been previously reported from both natural and man-mad aquatic environments [20, 22, 96, 98], further exemplifying the assertion that natural *Legionella* diversity is underestimated [6]. This is particularly true for underexplored environments, such as saline ecosystems. Moreover, our data presents novel *Legionella* genotypes that are able to survive and flourish in sodium-rich, high-temperature environments. One limitation of the study is that the data gathered is DNA based, and by its nature cannot differentiate between live and dead cells. None the less, the results provide a valid representation of the *Legionella* communities found at the different sites, and any dead cells that may be present likely originated from cells that have proliferated at the site sampled.

Variations in *Legionella* community structure in a saline environment have been previously reported by Shimada et al. (2021), for a sole sample taken from a saline Antarctic Lake, as compared to nearby freshwater lakes. Although relatively few ASVs were noted in the saline lake, the identity of predominant ASVs was shared with the freshwater lakes [20]. As temperatures and sodium concentrations measured in the spring clusters of our study were significantly higher, compared to those of the Arctic saline lake—it is likely that these *Legionella* genotypes are more adapted to such conditions.

We performed several attempts to isolate *Legionella* from the high temperature-saline environment of THS cluster, using both direct plating and inoculation in the presence of the amoeba *A. castellanii*. However, our efforts were unsuccessful. Indeed, *Legionella* are not readily isolated from saline environments with only two successful isolations reported [22, 39]. It is possible that isolation conditions were not optimal. However, the difficulty in culturing *Legionella* from saline high-temperature environments supports the idea that some *Legionella* species depend on their hosts for survival, potentially to the point of obligate endosymbiosis and therefore cannot be readily isolated by traditional methods [6, 99].

Co-occurrence analysis revealed that associations were present between *Legionella* and both *Ciliophora* and *Amoebozoa* in the water samples and *Ciliophora* only in the biofilm samples. These results were unexpected, as water samples presented predominantly *Ciliophora* ASVs and biofilm samples were more heterogeneous. Interestingly, the most common associations discovered in our analysis, were previously reported for both *Ciliophora* (*Oligohymenophorea*) and *Amoebozoa* (*Vannellidae*) [35, 100] Our co-occurrence results implicate certain species of *Ciliophora* and *Amoebozoa* as possible reservoirs for *Legionella* species under the high-salinity–high-temperature conditions prevailing in these environments. Host-specific Dot/Icm effector requirements have been demonstrated for *Legionella* in lab experiments [101, 102], and likely translate to environmental settings. Therefore, the range of host cells in the extreme environments studied here likely selects for *Legionella* genotypes that maintain the appropriate arsenal of Dot/Icm secreted effector proteins required for survival in these host cells. Our results demonstrate that the population of potential host cells found in saline environments is significantly less diverse than in freshwater environments. This, in turn, can dictate limited host-cycling, which is thought to explain the large number of effector proteins maintained in the *Legionella* genome [12]. Therefore, based on our data, we predict that in genotypes unique to saline environments you may find genetic adaptations resulting in the maintenance of only a small set of effector proteins, unlike in freshwater genotypes. Isolation of *Legionella* species unique to saline environments may reveal any such adaptations in the Dot/Icm system and in its effectors. It is worth noting that the novel genotypes identified in this study have not yet been demonstrated to survive predation, though this is likely to be the case, as all of the *Legionella* species studied to date were shown to replicate inside eukaryotic cells [103, 104].

It has been previously shown that in vitro selection of salt-resistant *L. pneumophila* mutants favors the growth of avirulent forms [37, 105, 106]. The ability of *Legionella* to survive and multiply within its protozoan hosts is mediated by the Dot/Icm secretion system [106, 107], which is also implicated in the phenotype of sodium sensitivity observed in *L. pneumophila* [40, 105]. *Legionella* strains from saline environments are continuously exposed to high sodium concentrations on the one hand, and grazing protists on the other. The combination of these selection pressures may favor *Legionella* forms presenting modified or alternative survival mechanisms. Several possible mechanisms can be considered: (a) an extracellular lifestyle where *Legionella* replicate independently of host cells, while evading protozoa and acquiring nutrients from the environment. (b) An obligate intracellular lifestyle where *Legionella* remain associated with host cells as endosymbionts, avoiding lytic release, and ensuring segregation between daughter cells during host cell division. (c) Nonlytic release into the environment [108], with *Legionella* remaining within an intact vacuole after infection without direct contact with the extracellular environment. (d) A modified Dot/Icm secretion system that includes structural components preventing the leakage of sodium through the system. (e) Interaction with alternative host cells in a manner not dependent on the Dot/Icm secretion system for survival in early stages of the infection, allowing for upregulation of the system only after entry into the host.

## Conclusion

Our study revealed an abundant community of novel *Legionella* genotypes that are not related to known species and are specific to ecosystems of high salinity and elevated temperature. Most *Legionella* studies have traditionally been conducted in vitro and on man-made environments. In laboratory strains, the combined effect of the two aforementioned conditions inhibits growth and selects for avirulent forms. In contrast, our study was conducted on natural springs and sought to uncover *Legionella* underexplored diversity in vivo, focusing on sodium-rich environments of high temperature. Our results reveal a unique subset of *Legionella* genotypes that can flourish in these extreme conditions. Concurrently, these genotypes are exposed to grazing pressure from protozoa that reside in those environments. Together with the interactions demonstrated with *Amoebozoa* and *Ciliophora* members, the data suggests that some *Legionella* species of saline environments may have evolved survival strategies distinct from those previously reported for *Legionella*. Isolation of such genotypes from saline environments in future work can uncover novel molecular mechanisms utilized by intracellular pathogens during the infection of host cells.

### Supplementary Information

**Additional file 1: Supplementary Methods. Supplementary Figure 1.** Map of sampling stations. **Supplementary Figure 2.** Principal-component analysis (PCA) of the various physiochemical characteristics measured at each sampling site. **Supplementary Figure 3.** Loading scores of Prinicipal-Component Analysis (PCA) of selected physiochemical characteristics measured at each sampling site, representing the four spring clusters. **Supplementary Figure 4.** Hierarchical K-means clustering of *Legionella* spp. abundance between different samples. **Supplementary Figure 5.** Rarefaction curves for Next generation Sequencing (NGS). **Supplementary Figure 6.** Analysis of batch effect between the Next Generation Sequencing (NGS) centers. **Supplementary Figure 7.** *Legionella* spp. levels do not correlate between water and biofilm samples. **Supplementary Figure 8.** Alpha and beta diversity of *Legionella* spp. microbial population in water vs. biofilm samples. **Supplementary Figure 9.** Venn diagram of *Legionella* spp. in water and biofilm samples. **Supplementary Figure 10.** Alpha diversity of *Legionella* spp. across spring clusters. **Supplementary Figure 11.** Alpha diversity of potential *Legionella* hosts. **Supplementary Figure 12.** Phylogenetic analysis of *Legionella* amplicon sequence variants (ASVs) in water samples. **Supplementary Figure 13.** Cooccurrence of Legionella ASVs with protozoan-hosts in Tabgha and Fuliya spring clusters. **Supplementary Table 1.** Physiochemical characteristics of selected springs stations surrounding lake Kinneret. **Supplementary Table 2.** Ranges of physicochemical characteristics for the studied springs sites surrounding lake Kinneret. **Supplementary Table 3.** Ranges of physicochemical characteristics representing springs clusters surrounding lake Kinneret. **Supplementary Table 4.** Loading scores of environmental variables included in the initial PCA analysis. **Supplementary Table 5.** Correlations between the physicochemical parameters comprising the initial PCA. **Supplementary Table 6.** Loading scores of environmental variables included in the final PCA analysis. **Supplementary Table 7.** Correlations between the selected physicochemical parameters included in the final PCA. **Supplementary Table 8.** K-means clustering summery table for qPCR heatmap (Fig. 2C). **Supplementary Table 9.** Kruskal-Wallis and post hoc via Wilcox test for *Legionella* spp. qPCR levels, between the different spring clusters and collection dates. **Supplementary Table 10.** Feature and sequence counts for all primer sets used in the study. **Supplementary Table 11.** Kruskal-Wallis test results of 16S NGS alpha diversity between the two sequencing centers. **Supplementary Table 12.** PERMANOVA, PERMDISP and pairwise post hoc results of 16S NGS beta between the two sequencing centers, collection years and water vs. biofilm. **Supplementary Table 13.** Kruskal-Wallis and post hoc via Wilcox tests of 16S NGS alpha diversity between the different spring clusters and collection dates. **Supplementary Table 14.** PERMANOVA and PERMDISP results of 16S NGS beta diversity between the different spring clusters. **Supplementary Table 15.** PERMANOVA and PERMDISP post hoc pairwise comparison results of 16S NGS beta diversity between the different spring clusters. **Supplementary Table 16.** ADONIS test results of 16S NGS beta diversity with selected environmental variables. **Supplementary Table 17.** Kruskal-Wallis test results of 18S NGS alpha diversity between the two sequencing centers. **Supplementary Table 18.** PERMANOVA and PERMDISP results of 18S NGS beta diversity between the two sequencing centers and collection years. **Supplementary Table 19.** Kruskal-Wallis and post hoc via Wilcox tests of 18S NGS alpha diversity between the different spring clusters and collection dates. **Supplementary Table 20.** PERMANOVA and PERMDISP results of 18S NGS beta diversity between the different spring clusters. **Supplementary Table 21.** PERMANOVA and PERMDISP post hoc pairwise comparison results of 18S NGS beta diversity between the different spring clusters.

**Additional file 2: Supplementary Data 1.** 16S *Legionella* ASV table. For each ASV, the full MD5 code is presented, including the last five characters identifier. Additional information provided is occurrence per sample, QIIME2 classification and representative sequence. For each sample identifier we included description of sampling station, spring cluster, collection date sampling environment (water/biofilm) and experiment Sample ID.

**Additional file 3: Supplementary Data 2.** 18S potential *Legionella*-hosts ASV table. Included ASVs are representatives of Amoebozoa, Ciliophora and Percolozoa. For each ASV, the full MD5 code is presented, including the last five characters identifier. Additional information provided is occurrence per sample, QIIME2 classification and representative sequence. For each sample identifier we included description of sampling station, spring cluster, collection date sampling environment (water/biofilm) and experiment Sample ID.

**Additional file 4: Supplementary Data 3.** *Legionella* reference sequences. Reference bacteria were used during the generation of all phylogenetic trees and include Accession Number. *Coxiella burnetii* was included as the outgroup.

**Additional file 5: Supplementary Data 4.** Dominant *Legionella* 16S ASVs. Only ASVs with relative abundance >0.5 are presented. For each ASV, the last five characters identifier code is presented, alongside Sample ID and spring cluster.

**Additional file 6: Supplementary Data 5.** Random Forest output tables. randomForest r package output tables are presented for Water (Sheet 1) and biofilm (Sheet 2). Water and biofilm importance tables (Sheets 3 and 4), indicating ASVs importance were measured for the regression procedure, using the decrease in mean squared error (%IncMSE).

## Data Availability

Raw metagenomic sequence reads generated in this study have been deposited in the European Nucleotide Archive Database (https://www.ebi.ac.uk/ena/browser/home) as project accession number PRJEB53014. Additional data are available under the Supplementary Information and Supplementary Data sections. The datasets and scripts that were used for the analyses presented in the manuscript have been uploaded to the GitHub repository at: https://github.com/BergmanOded/LegionellaSodiumSpring
