# Correction: Sodium levels and grazing pressure shape natural communities of the intracellular pathogen *Legionella*

**DOI:** 10.1186/s40168-023-01639-2

**Published:** 2023-08-07

**Authors:** Oded Bergman, Yaron Be’eri-Shlevin, Shira Ninio

**Affiliations:** Kinneret Limnological Laboratory (KLL), Israel Oceanographic and Limnological Research (IOLR), P.O. Box 447, 49500 Migdal, Israel

**Correction: Microbiome 11, 167 (2023)**

**https://doi.org/10.1186/s40168-023-01611-0**

Following the publication of the original article [1], the author reported a typographical error in the first sentence of the abstract section. "human disease outbarks" should read "human disease outbreaks"

The original article has been updated.

## References

[1] Bergman O, Be’eri-Shlevin Y, Ninio S (2023). Sodium levels and grazing pressure shape natural communities of the intracellular pathogen Legionella. Microbiome.

