# Supplementary material for: Sodium levels and grazing pressure shape natural communities of the intracellular pathogen Legionella
Source: Microbiome. 2023 Jul 31;11:167. doi: 10.1186/s40168-023-01611-0 (PMC10388490; doi:10.1186/s40168-023-01611-0)
Supplement: Supplementary file 2 — Additional file 1: Supplementary Methods. Supplementary Figure 1. Map of sampling stations. Supplementary Figure 2. Principal-component analysis (PCA) of the various physiochemical characteristics measured at each sampling site. Supplementary Figure 3. Loading scores of Prinicipal-Component Analysis (PCA) of selected physiochemical characteristics measured at each sampling site, representing the four spring clusters. Supplementary Figure 4. Hierarchical K-means clustering of Legionella spp. abundance between different samples. Supplementary Figure 5. Rarefaction curves for Next generation Sequencing (NGS). Supplementary Figure 6. Analysis of batch effect between the Next Generation Sequencing (NGS) centers. Supplementary Figure 7. Legionella spp. levels do not correlate between water and biofilm samples. Supplementary Figure 8. Alpha and beta diversity of Legionella spp. microbial population in water vs. biofilm samples. Supplementary Figure 9. Venn diagram of Legionella spp. in water and biofilm samples. Supplementary Figure 10. Alpha diversity of Legionella spp. across spring clusters. Supplementary Figure 11. Alpha diversity of potential Legionella hosts. Supplementary Figure 12. Phylogenetic analysis of Legionella amplicon sequence variants (ASVs) in water samples. Supplementary Figure 13. Cooccurrence of Legionella ASVs with protozoan-hosts in Tabgha and Fuliya spring clusters. Supplementary Table 1. Physiochemical characteristics of selected springs stations surrounding lake Kinneret. Supplementary Table 2. Ranges of physicochemical characteristics for the studied springs sites surrounding lake Kinneret. Supplementary Table 3. Ranges of physicochemical characteristics representing springs clusters surrounding lake Kinneret. Supplementary Table 4. Loading scores of environmental variables included in the initial PCA analysis. Supplementary Table 5. Correlations between the physicochemical parameters comprising the initial PCA. Supplementary Table 6. Loading sco [file 40168_2023_1611_MOESM1_ESM.pdf]

Supplementary Information

Sodium levels and grazing pressure shape natural communities of the intracellular pathogen *Legionella*

O. Bergman[1], Y. Be'eri-Shlevin[1], S. Ninio[1*]

Kinneret Limnological Laboratory (KLL) Israel Oceanographic and Limnological Research (IOLR), P.O.Box 447, Migdal, 49500, Israel,

Mailing address: shira.ninio@ocean.org.il

* Corresponding Author

The following are included:

Supplementary methods

Supplementary Figs 1-13

Supplementary Tables 1-21

Supplementary References

Supplementary Methods

LMM model selection and fitting

The LMM model selection and fitting procedure has been extensively described by Zuur and others[1–3]. First, considering collinearity between the explanatory variables[4] (Supplementary Table 7), we omitted NO_3^- and SO_4 from the analysis as they showed high correlation with Na and temperature. As temperature, pH and Na have been previously reported as important factors in *Legionella* spp. growth[5,6], all three were kept in the analysis. Thus, the final fixed factor structure for water samples consisted of: Na, temperature, Fe and pH. For biofilm temperature was removed, due to high collinearity with Na. Utilizing a constant fixed variables structure, we first established the optimal random effects structure, using restricted maximum likelihood (REML) estimation, via the lme4[7] and AICcmodavg[8] packages. We included sampling stations and collection dates as crossed random effects and considered random intercepts, random slopes and the combination of both. The scaled-centered log values of all continues variables and log+1 values of *Legionells* spp. qPCR levels were used in the fitting process of all models. We then checked for collinearity between explanatory variables and excluded models presenting high IVF values (>3)[1]. The final random structure was selected using the Second-order Akaike's information criterion (AICc), with the model demonstrating lowest value chosen. If models differed by less than 2 AICc units, the model with less components was selected (the simplest model). In the second stage we fitted the fixed effects structure of the model, keeping the selected random-effects structure constant. All possible fixed-effects structure models were considered. The lowest AICc method was used for final fixed-effects structure selection, using ML estimation. The selected models were finally fitted using the REML estimation.

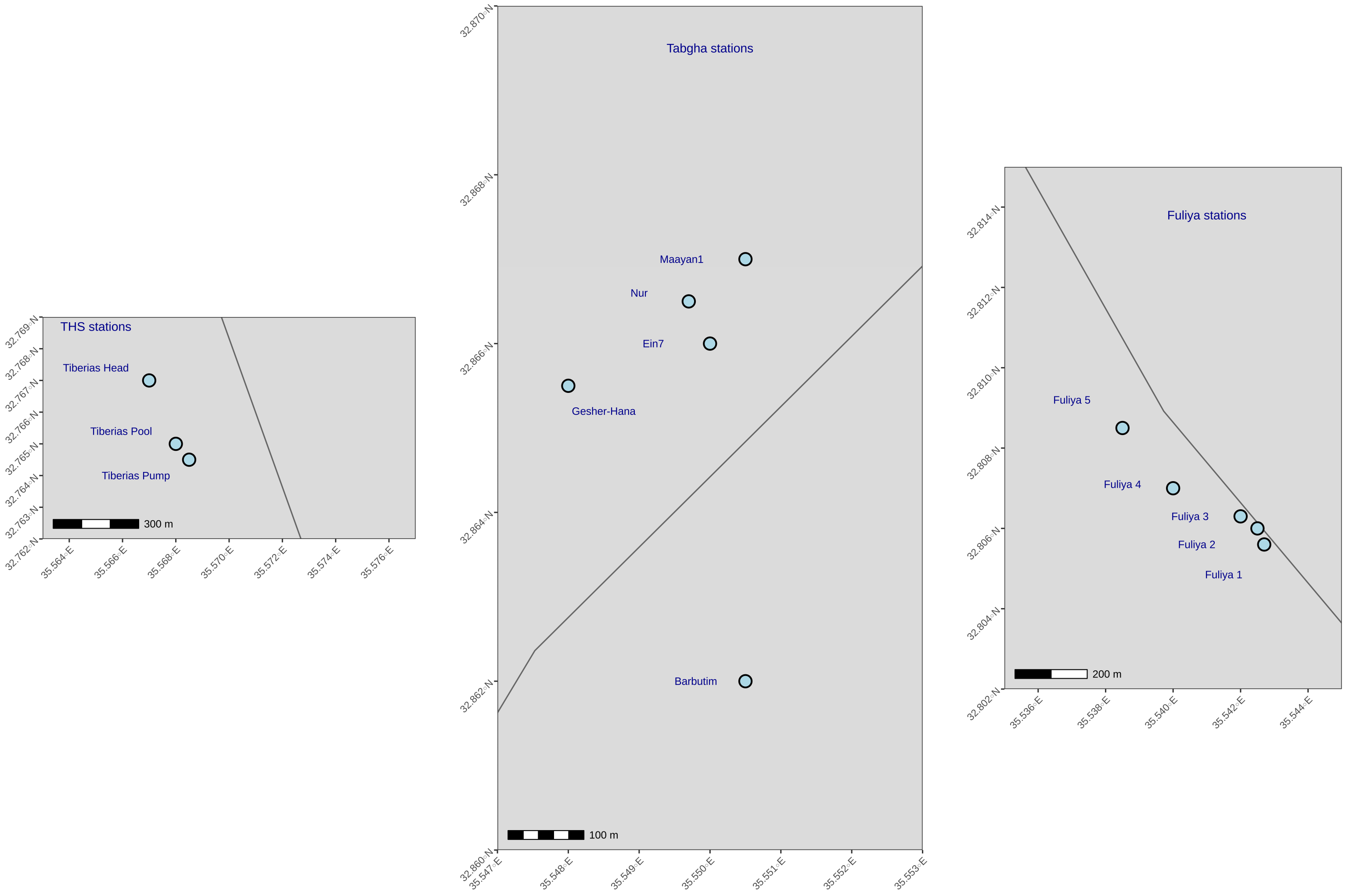

Supplementary Figure 1. Map of sampling stations. Sampling stations included saline springs located within and around the Sea of Galilee, representing four spring clusters: Tiberias Hot Spring (THS) stations (left panel), Tabgha stations (middle panel) and Fuliya stations (right panel). Barbutim spring is located within the sea of galilee and is related to the Tabgha cluster.

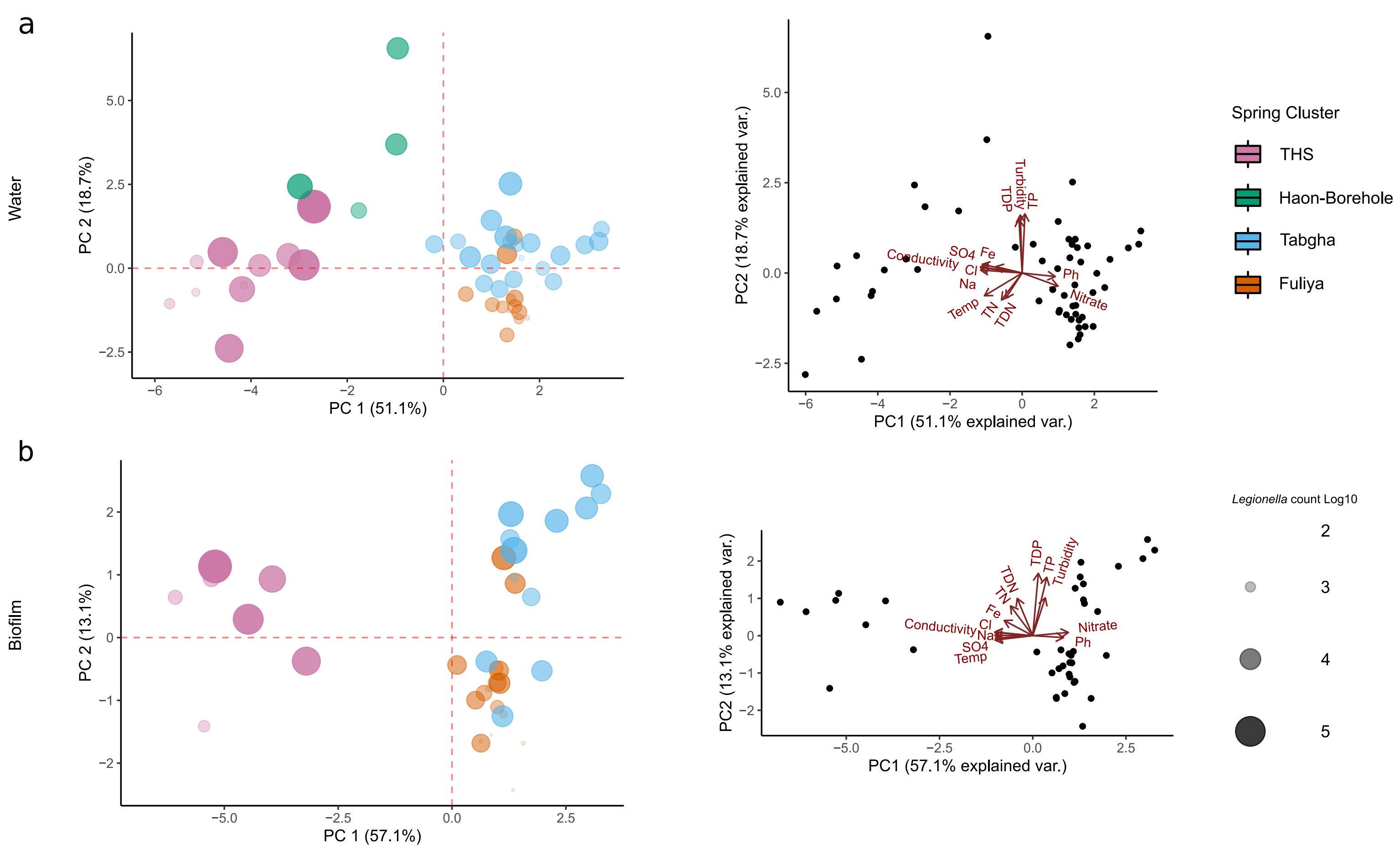

Supplementary Figure 2. Principal-component analysis (PCA) of the various physicochemical characteristics measured at each sampling site. PCA was performed on (a) water (n=55) and (b) biofilm (n=39) samples (left panels) obtained between January 2018 and January 2019 and included the following environmental variables: Temperature, Ph, Turbidity, Na, Cl, Conductivity, total and dissolved phosphorus (TP and TDP), total and dissolved nitrogen (TN and TDN), NO3-, SO4-2 and Fe. Size and transparency represent the log+1 values of qPCR *Legionella* spp. levels, from the various sampling sites. Quantification (by qPCR) was done using primers and probes specific to *Legionella* spp., targeting the 16S rRNA gene. Loading vectors, indicating the importance of tested environmental variables related to PC 1 and PC 2 (right panels), are based on the PCA's loading scores (Supplementary Table 3). Spring cluster colours: Tiberias Hot Springs (purple), Haon Borehole (green), Fuliya (red) and Tabgha (cyan). THS = Tiberias Hot Springs.

a

b

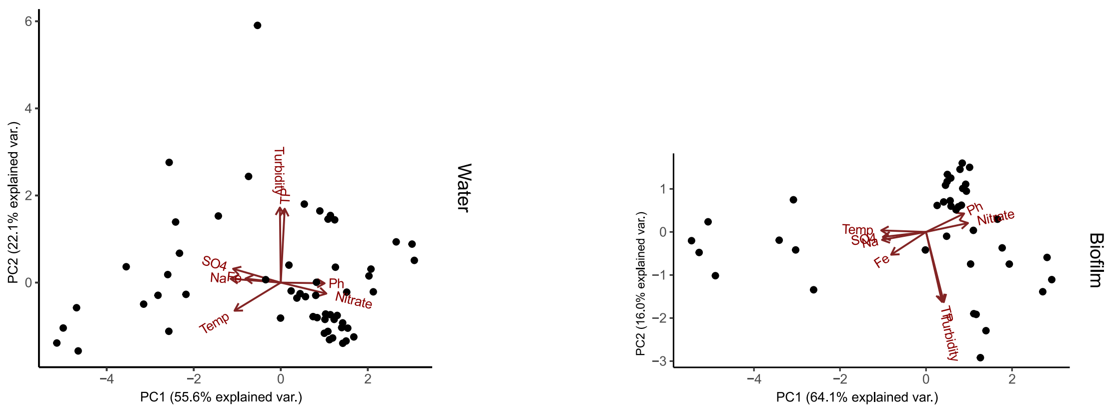

Supplementary Figure 3. Loading scores of Principal-Component Analysis (PCA) of selected physicochemical characteristics measured at each sampling site, representing the four spring clusters. The PCA was performed on water (n=55) and biofilm (n=39) samples, obtained between January 2018 and January 2019 and included the following environmental variables: Temperature, Ph, Na, Turbidity, TP, SO4, NO3- and Fe. Loading vectors of (a) water and (b) biofilm, indicating the importance of tested environmental variables related to PC 1 and PC 2, are based on the PCA's loading scores (Supplementary Table 5). The environmental variables were chosen following a PCA analysis from a larger subset of environmental factors (see Supplementary Fig. 2 and Supplementary Tables 3 and 4).

a

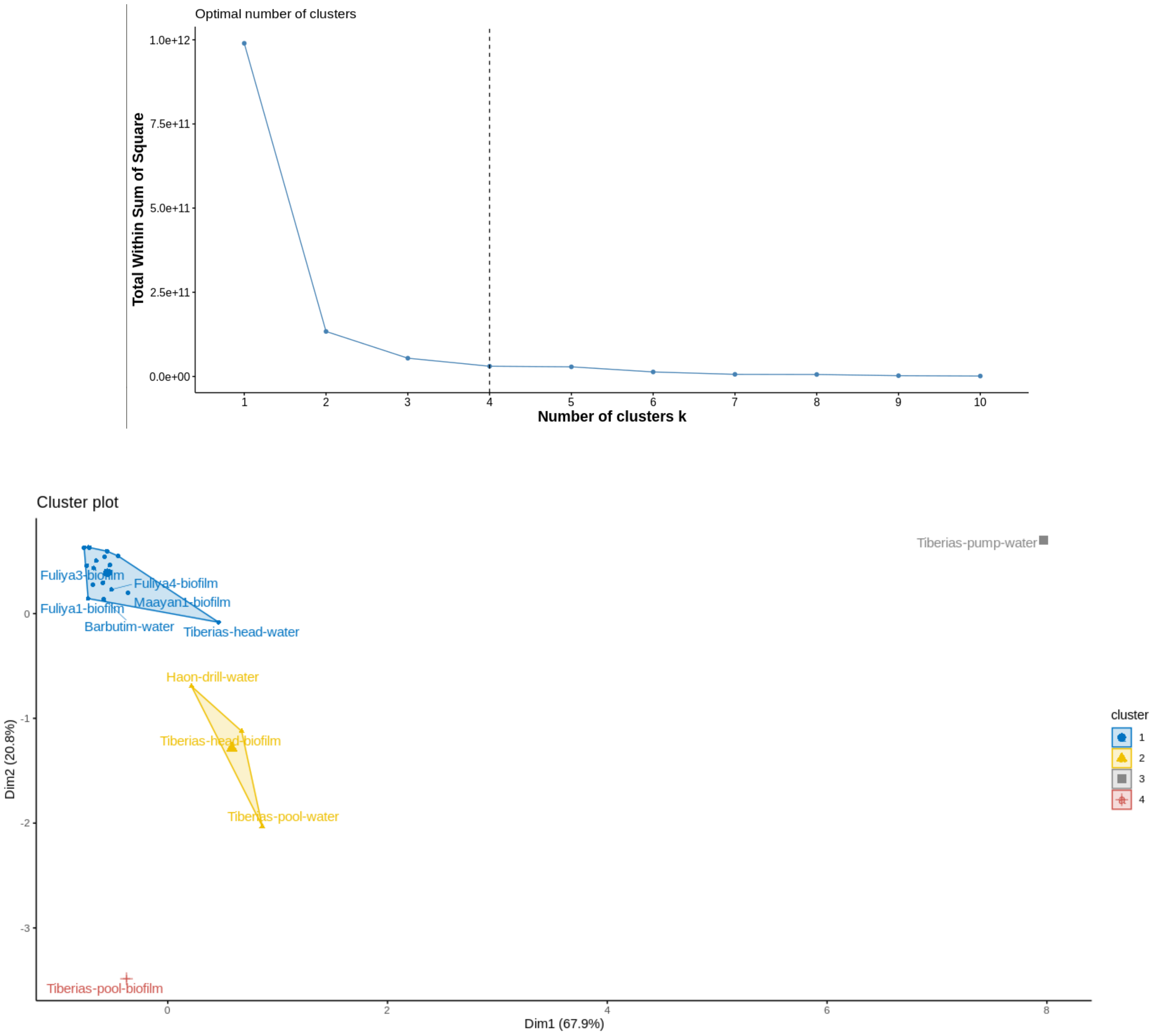

Supplementary Figure 4. Hierarchical K-Means clustering of *Legionella* spp. abundance between the different samples. Quantification (by qPCR) was done using primers and probes specific to *Legionella* spp., targeting the 16S rRNA gene. Abundance was analyzed in water (n=64) and biofilm (n=50) samples. (a) Decline in within sample variation indicated the optimal number of clusters and was determined using the k-means function (stats package). (b) Principal Component Analysis (PCA) visualization of Hierarchical K-Means Clustering represents the different sample's clustering vectors. The analysis was performed in r using the factoextra package, prior to heatmap construction (see also Supplementary Table 8).

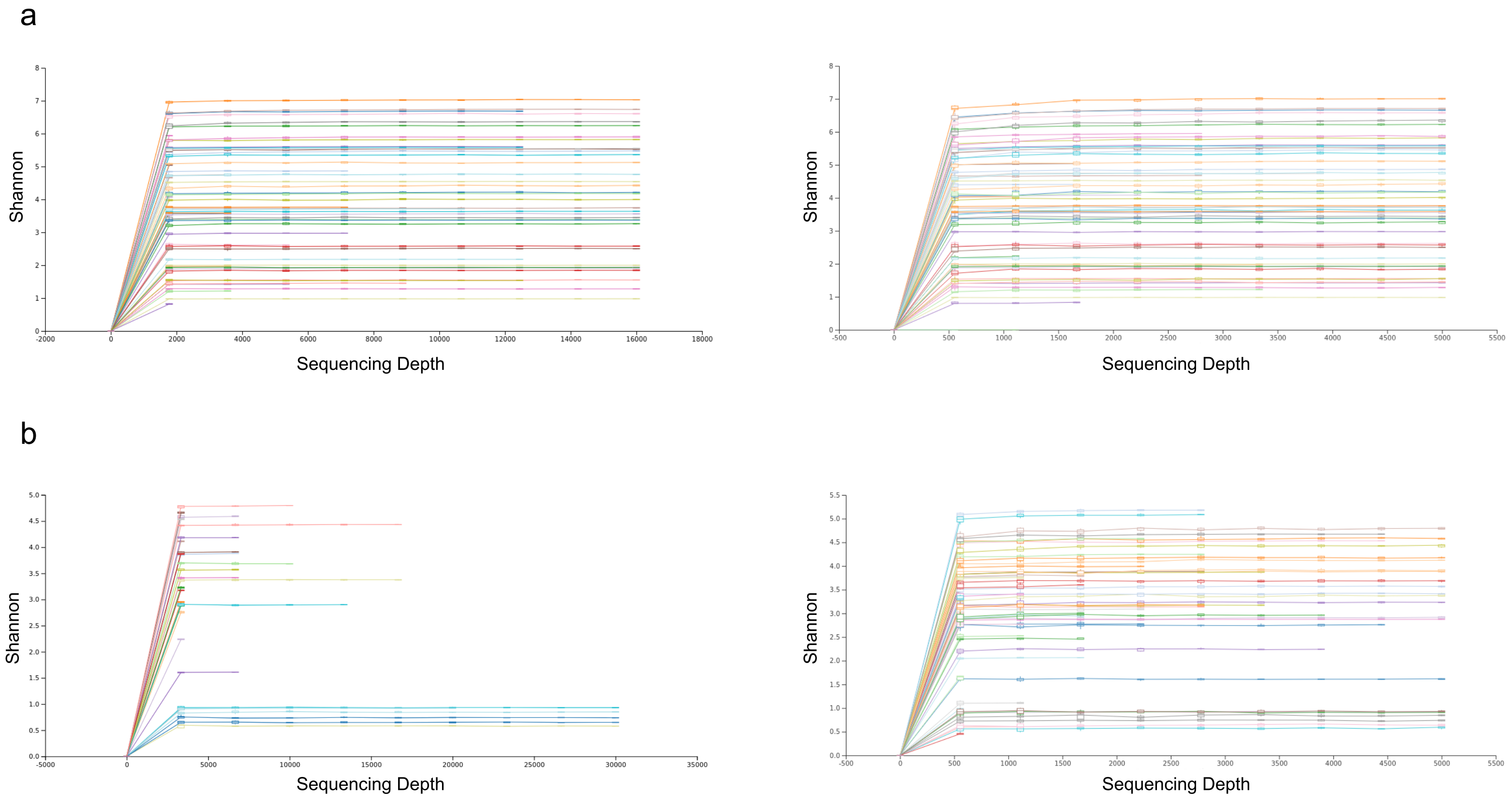

Supplementary Figure 5. Rarefaction curves for Next generation Sequencing (NGS). NGS was performed using *Legionella* spp. specific 16S primers and 18S universal primers targeting protists, designed for the V9 region (Lgsp17F-Lgsp28R and euk1391fF-eukBr primer set, respectively). Raw data were pre-filtered to retain *Legionella* Amplicon Sequence Variants (ASVs), present at a minimal frequency of 20 reads across all samples, in a minimum of 2 samples. (a) rarefaction curves for the 16S *Legionella* primer set at a sequencing depth of the median (16001 reads, left panel) and at a sequencing depth of 5000 reads, to justify the rarifying parameter of 1222 chosen for the diversity analysis (right panel). (b) Rarefaction curves for the 18S primer set at a sequencing depth of the median (30000 reads, left panel) and 5000 reads, to justify the rarifying parameter of 986 chosen for the diversity analysis (right panel). Rarefaction curves were generated using the QIIME2 alpha-rarefaction function of the q2-diversity plugin.

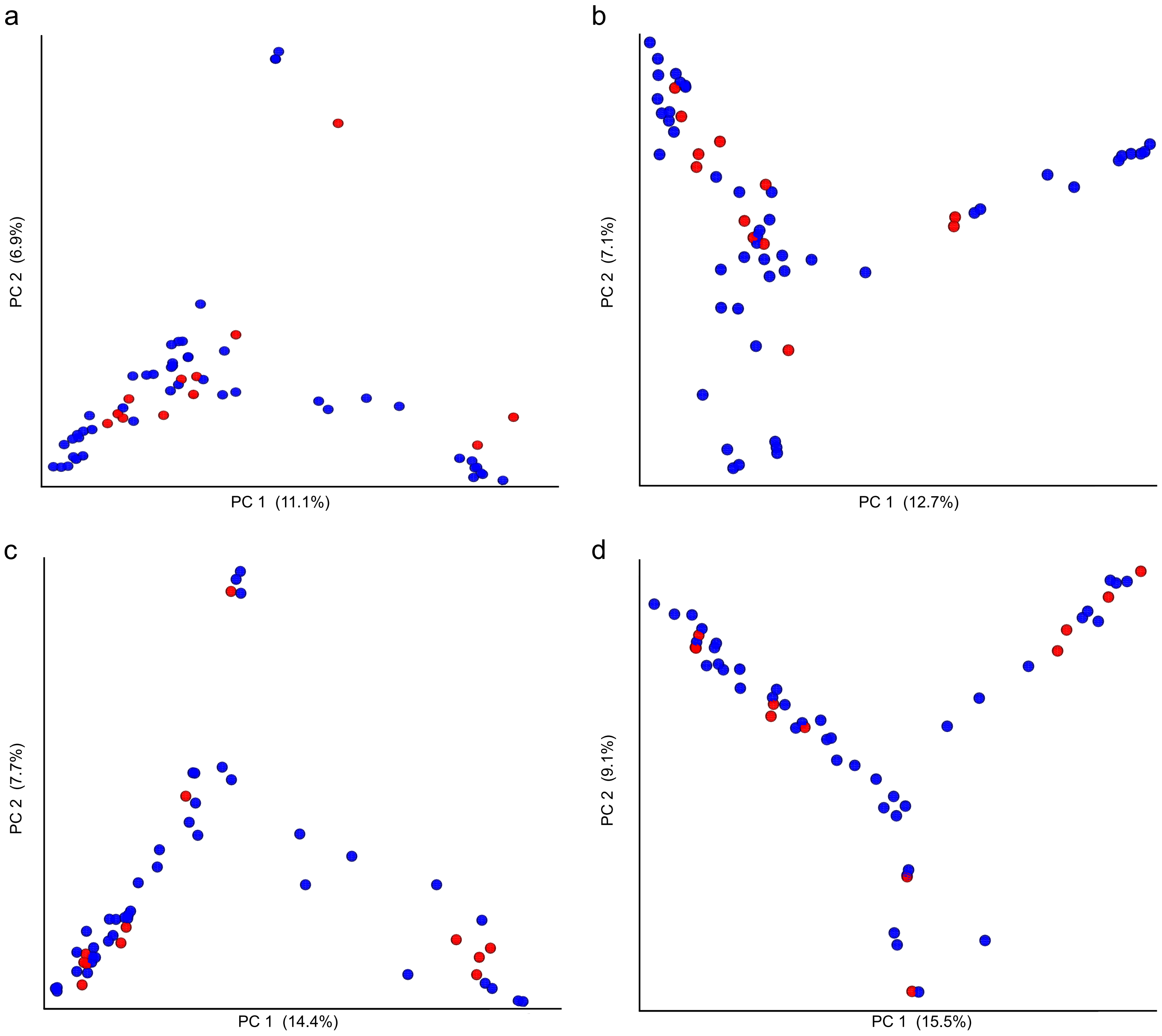

Supplementary Figure 6. Analysis of batch effect between the Next Generation Sequencing (NGS) centers. Library preparation and sequencing of September-2017 samples were performed at MR-DNA sequencing center (n=12), while the remaining 2018-2019 samples (n=32 and n=16, respectively), were sent to the Research Resources Center (RRC) sequencing center, University of Illinois at Chicago (UIC). NGS was performed using *Legionella* spp. specific 16S primers and 18S universal primers targeting protists, designed for the V9 region. Prior to beta diversity analyses, Amplicon Sequence Variants (ASVs) for the 16S dataset were filtered to only retain *Legionella* spp. For the 18S dataset ASVs filtering was performed for Amoebozoa, Cliophora (Ciliates) and Percolozoa (Excavata). Raw data were pre-filtered to retain a minimal frequency of 20 reads across all samples, in a minimum of 2 samples. Batch effect was assessed by Jaccard and Bray–Curtis for the 16S dataset (a) and (b), and for the 18S primer set, (c) and (d), respectively.

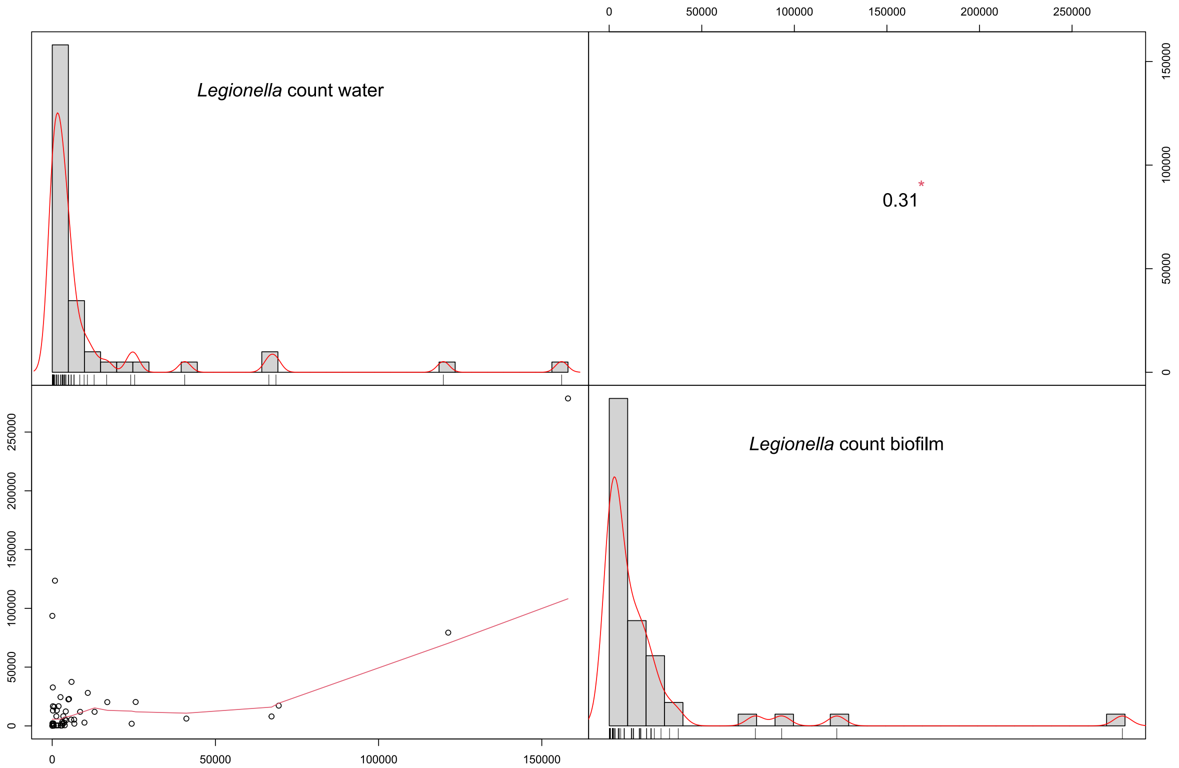

Supplementary Figure 7. *Legionella* spp. levels do not correlate between water and biofilm samples. qPCR levels were measured by qPCR using *Legionella* spp. specific primers targeting the 16S rRNA gene. Spearman coefficient was used to correlate *Legionella* spp. between water and biofilm samples (n=49).

a

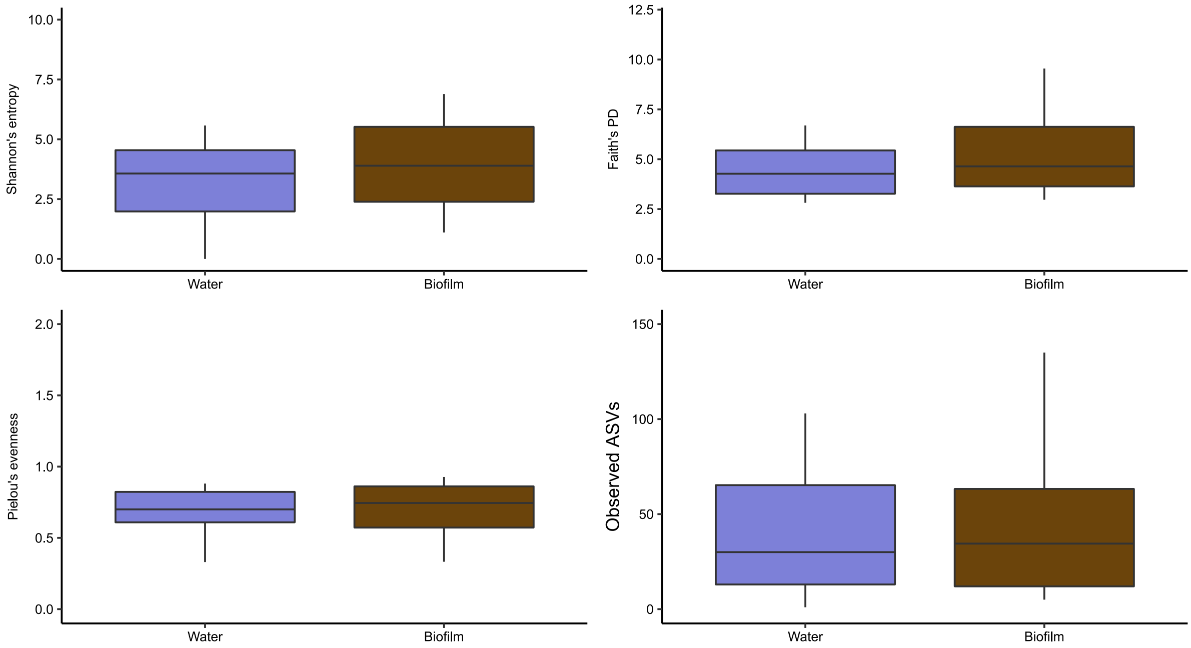

b

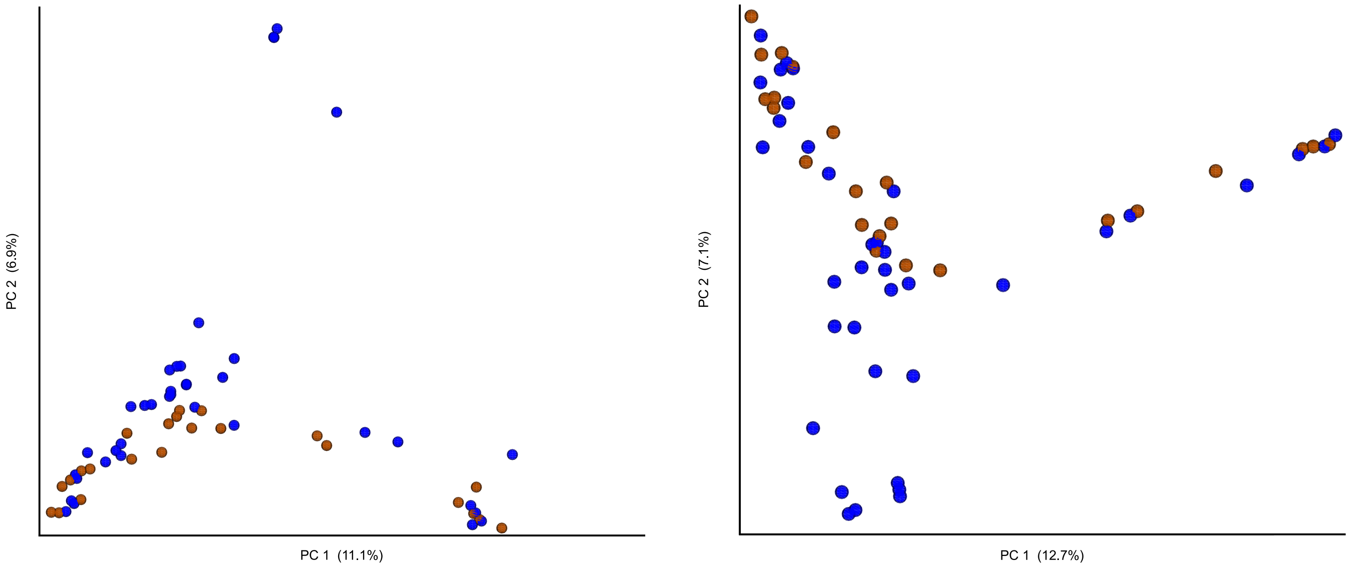

Supplementary Figure 8. Alpha and beta diversity of *Legionella* spp. microbial population in water vs. biofilm samples. Next Generation Sequencing (NGS) was performed using *Legionella* spp. specific 16S primers. Raw data were pre-filtered to retain *Legionella* Amplicon Sequence Variants (ASVs), present at a minimal frequency of 20 reads across all samples, in a minimum of 2 samples. (a) Shannon's entropy, Faith's PD, Pielou's evenness and Observed OTUs were included, for water (n=37) and biofilm (n=23) samples. Kruskal-Wallis test results and post hoc via Wilcox tests are presented in Supplementary Table 11. (b) Beta diversity of *Legionella* spp. was assessed via Jaccard (left panel) and Bray–Curtis (right panel) and presented for water (left panel) and biofilm (right panel) samples. Statistical significance was tested via PERMANOVA and PERMDISP analyses (Supplementary Table 10).

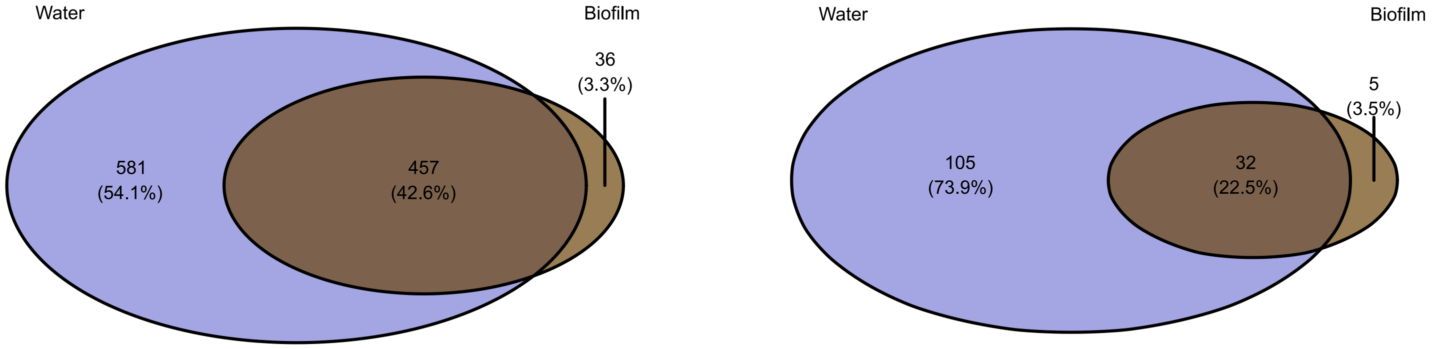

Supplementary Figure 9. Venn diagram of *Legionella* spp. in water and biofilm samples. Next Generation Sequencing (NGS) performed using *Legionella* spp. specific 16S primers. Raw data were pre-filtered to retain *Legionella* Amplicon Sequence Variants (ASVs), present at a minimal frequency of 20 reads across all samples, in a minimum of 2 samples. Water (blue, n=40) and biofilm (brown, n=25) samples of all spring-clusters are presented in the left panel. Water (blue, n=12) and biofilm (brown, n=8) samples of Tiberias Hot Springs (THS) cluster are presented in the right panel.

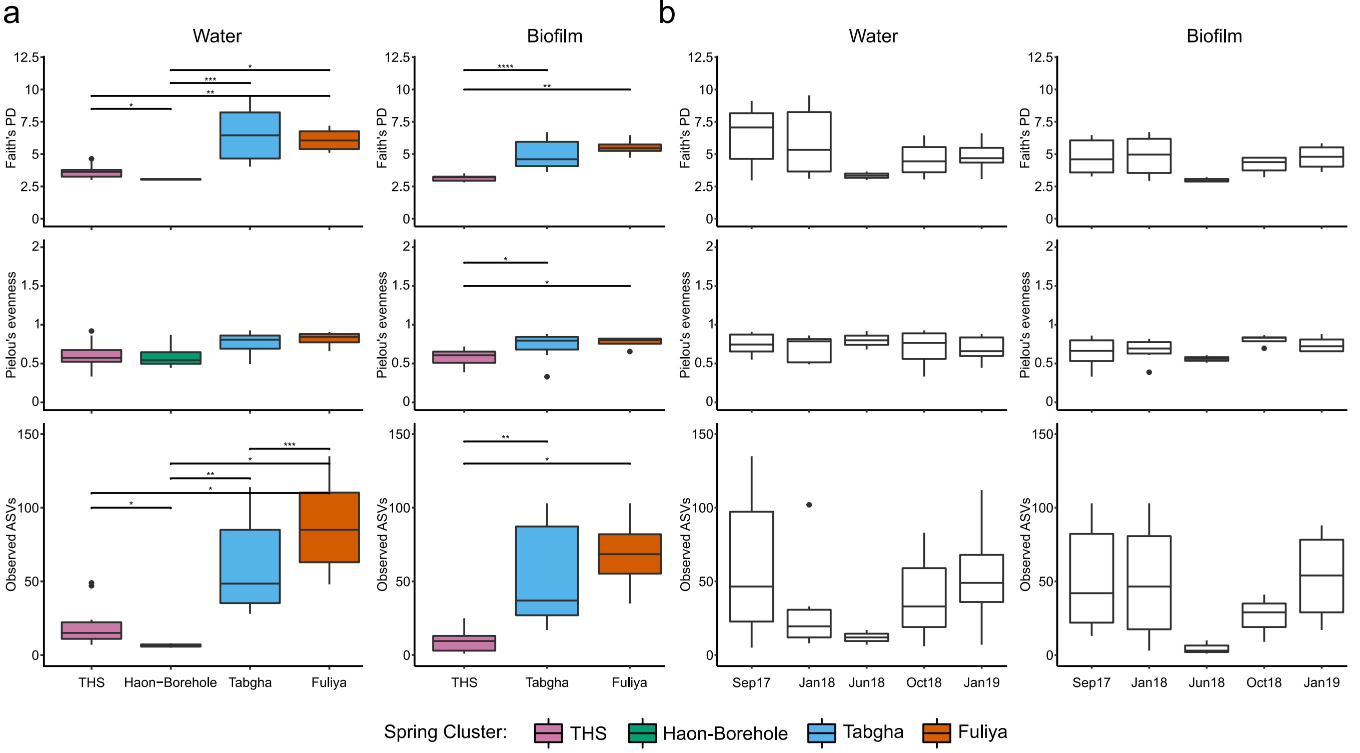

Supplementary Figure 10. Alpha diversity of *Legionella* spp. across spring clusters. *Legionella* diversity was examined following Next Generation Sequencing (NGS) performed using *Legionella* spp. specific 16S primers. Raw data were pre-filtered to retain *Legionella* Amplicon Sequence Variants (ASVs), present at a minimal frequency of 20 reads across all samples, in a minimum of 2 samples. Alpha diversity analyses were performed at a rarefaction depth of 1222 reads. Water (n=40) and biofilm (n=25) samples were analyzed separately. Spring cluster colours: Tiberias Hot Springs (n=20, purple), Haon Borehole (n=4, green), Fuliya (n=8, red) and Tabgha (n=33, cyan). Alpha diversity of *Legionella* spp. is presented across spring clusters (a) and collection dates (b). Faith’s PD, Pielou’s evenness and Observed ASVs are presented for water (left panels) and biofilm (right panels) samples. Kruskal-Wallis test results and post hoc via Wilcox tests are presented in Supplementary Table 12. THS = Tiberias Hot Springs. Significance level; * p ≤ 0.05; ** p ≤ 0.01; *** p ≤ 0.001, **** p ≤ 0.0001.

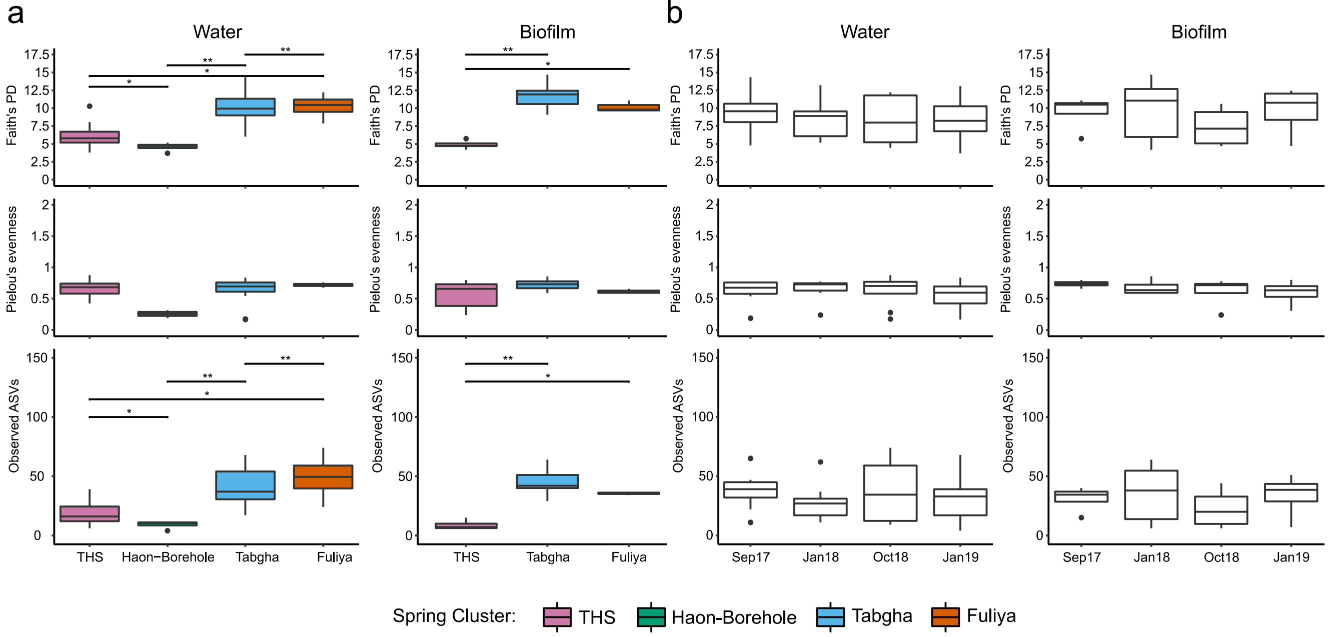

Supplementary Figure 11. Alpha diversity of potential *Legionella* hosts. Next Generation Sequencing (NGS) was performed using universal primers, designed for the V9 region of the 18S rRNA gene. Raw data were pre-filtered to retain Amoebozoa, Cliophora (Ciliates) and Percolozoa (Excavata) Amplicon Sequence Variants (ASVs), present at a minimal frequency of 20 reads across all samples, in a minimum of 2 samples. Water (n=40) and biofilm (n=25) samples were analyzed separately. Alpha diversity analyses were performed at a rarefaction depth of 986 reads. Alpha diversity of Potential *Legionella* hosts is presented across spring clusters (a) and collection dates (b). Faith's PD, Pielou's evenness and Observed ASVs are presented for water (left panel) and biofilm (right panel) samples. Kruskal-Wallis test results and post hoc via Wilcox tests are presented in Supplementary Table 18. Spring cluster colours: Tiberias Hot Springs (purple), Haon Borhole (green), Fuliya (red) and Tabgha (cyan). THS = Tiberias Hot Springs.

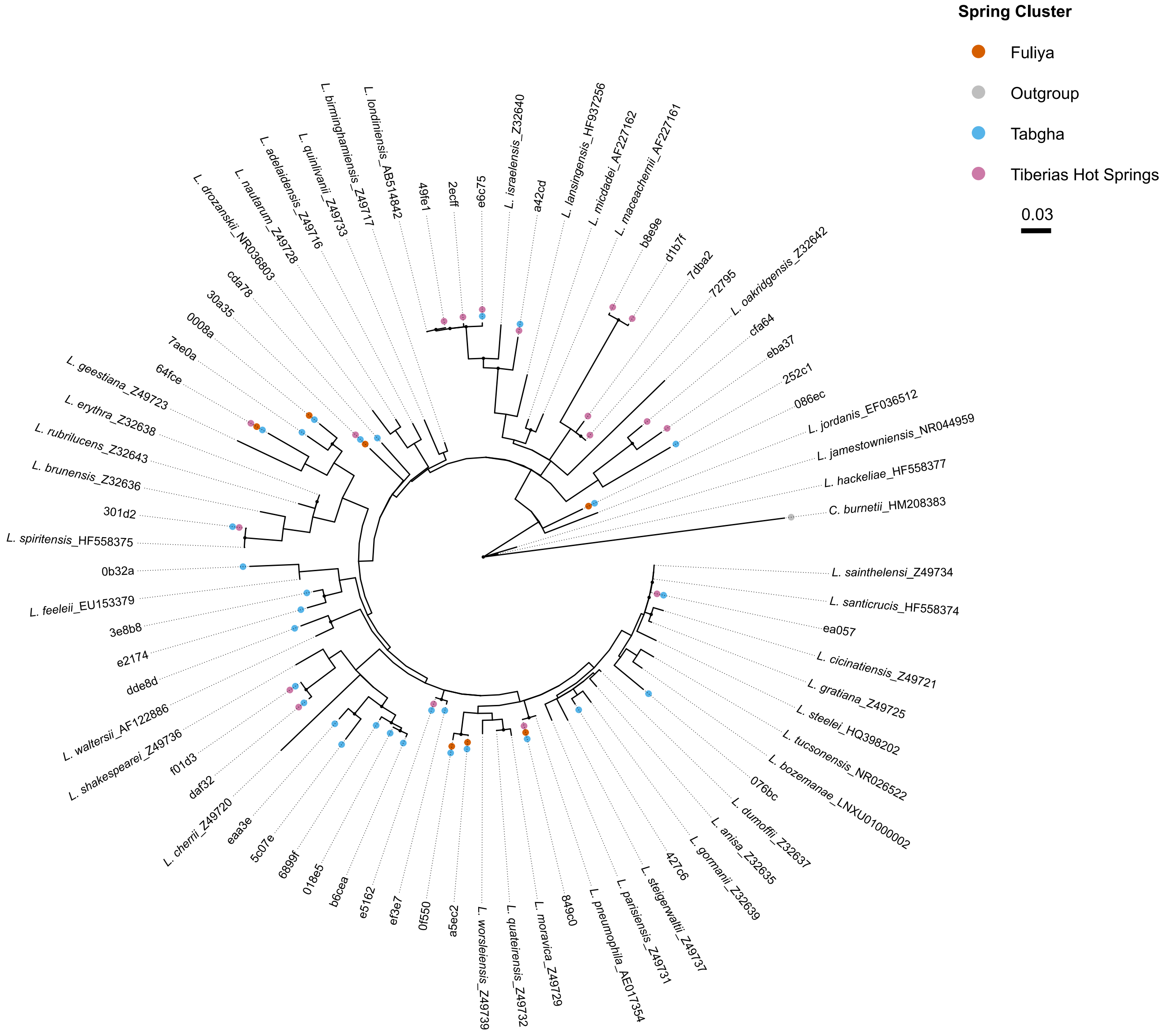

Supplementary Figure 12. Phylogenetic analysis of *Legionella* amplicon sequence variants (ASVs) in water samples. Phylogenetic trees were constructed alongside reference bacteria with corresponding NCBI accession-numbers (Supplementary Data 3). Predominant ASVs (filtered for minimum of 4,714 reads (0.5%), in 2 samples) are presented for water samples, from all Tabgha, Fuliya and Tiberias Hot Springs (THS) spring clusters. The evolutionary history of the tree was inferred using the Maximum-Likelihood method and Hasegawa-Kishino-Yano (HKY) model. *Coxiella burnetii* was included as an outgroup. The tree with the highest log likelihood is presented (-3143.71). A discrete Gamma distribution was used to model evolutionary rate differences among sites (5 categories (+G, parameter = 0.1796)). The rate variation model allowed for some sites to be evolutionarily invariable ([+I], 35.05% sites). The tree is drawn to scale, with branch lengths measured in the number of substitutions per site. Branches with bootstrap values above 50% are demoted by a black dot. The analysis involved 77 nucleotide sequences. There were a total of 378 positions in the final dataset. Evolutionary analyses were conducted in MEGA11.

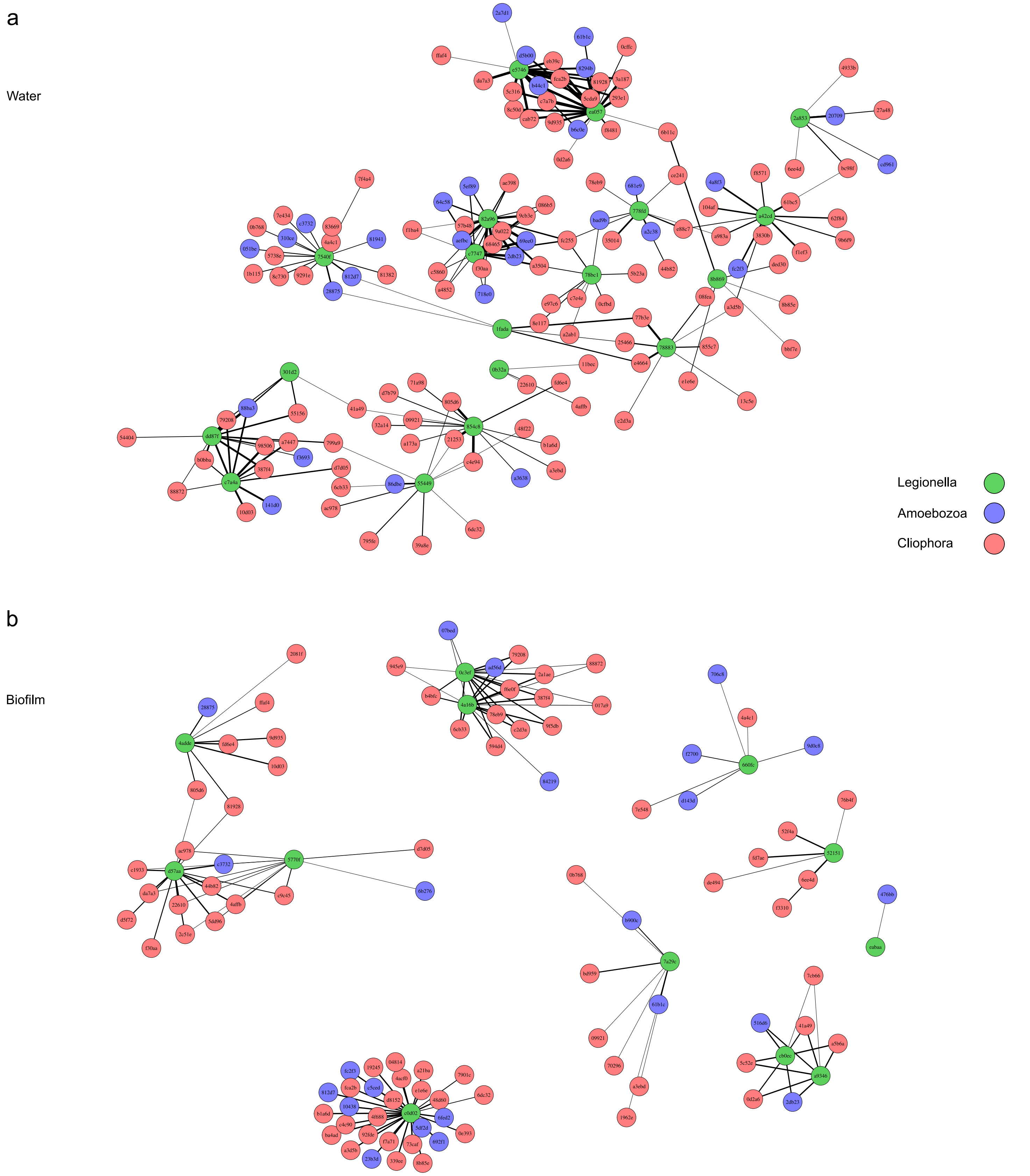

Supplementary Figure 13. Cooccurrence of Legionella ASVs with protozoan-hosts in Tabgha and Fuliya spring clusters. SPIEC-EASI cross-domain cooccurrence networks of *Legionella* ASVs and potential-protozoan hosts, associated with Tabgha (n=24) and Fuliya (n=8) spring-clusters, in (a) water and (b) biofilm samples. Networks are based on next Generation Sequencing (NGS) performed using *Legionella* spp. specific 16S primers. Raw data were pre-filtered to retain *Legionella* Amplicon Sequence Variants (ASVs). NGS-amplicon sequencing targeting protozoa was performed using 18S rRNA primers directed to the V9 region. Raw data were pre-filtered to retain Amoebozoa, Cliophora and Percolozoa ASVs. We included ASVs present at a minimal frequency of 20 reads across all samples, in a minimum of 2 samples. We only included *Legionella* ASVs present in the top 30 ASV-importance table, of the random forest procedure. ASVs are represented by the last 5 letters of a letter-number code. For the full-length code and corresponding sequence, see Supplementary Data files 2 and 3. Edges with low weights (>0.01) and unconnected nodes were removed. Only positive associations between ASV nodes are presented. Edge width corresponds to associations strength between ASVs.

Supplementary Table 1: Physicochemical characteristics of selected springs stations surrounding lake Kinneret.

Station	Date	Spring cluster	Temp (°C)	Cl (mM)	Na (mM)	Ph	Turbidity (NTU)	TDP (mg/L)	TP (mg/L)	TDN (µg/L)	TN (µg/L)	NO_3^- (µg/L)	SO4 (mg/L)	Conductivity (µS/cm)	Fe (µg/L)
**Fuliya5**	Sep17	Fuliya	29	23.8	17.2	7.2	0.4	NA	22.6	NA	4.4	NA	NA	3120	NA
	Jan18		28	23.7	17.1	7.1	1.2	10.7	14	4.3	4.5	3.5	83	3210	4.2
	Jun18		28	24.7	17.8	7.5	0.7	8.4	9.2	5.3	5.1	4.7	104	3400	3.4
	Oct18		27	21.7	15.6	7.4	1.2	7.6	15	5.2	5.9	4	99	2890	5.6
	Jan19		27.5	26.5	19.1	7.5	1.8	10.4	10.4	1.1	4.2	1	50	3380	0.2
**Fuliya4**	Sep17	Fuliya	30	26.5	19.1	7.3	5.2	NA	24.7	NA	3.7	NA	NA	3420	NA
	Jan18		26	26.2	18.9	7.3	1.9	9.1	11.6	4.1	4.1	3.3	95	3490	5.8
	Jun18		28	27.6	19.9	7.6	1.2	6.7	11.4	4.5	5	4.4	110	3720	3.7
	Oct18		27	25.4	18.3	7.4	1.5	6.7	151.7	4.4	6.8	3.5	110	3210	9.9
	Jan19		23	29.7	21.4	7.5	2.9	8.1	93.5	3.1	4.3	2.4	133	3650	8.9
**Fuliya3**	Sep17	Fuliya	31	43	31.0	7.1	0.7	NA	19.7	NA	2.5	NA	NA	5180	NA
	Jan18		27	25.9	18.6	7.6	2.7	8.6	8.5	4.4	4.9	2.9	93	3440	6.7
	Jun18		26	30.3	21.8	7.8	0.9	8.4	8.8	4.3	4.6	4	125	4020	2.6
	Oct18		30	39.3	28.3	7.3	3.1	6.4	16.6	4.2	4.9	3	170	4820	11.3
	Jan19		29	45.7	32.9	7.4	2.2	6.3	10.3	2.9	3.3	2.6	155	5410	0.9
**Fuliya2**	Sep17	Fuliya	30	29.1	20.9	7.2	1.2	NA	20.6	NA	3	NA	NA	3630	NA
	Jan18		29	33.4	24.0	7.5	1.3	7.3	13.5	4.1	4.1	2.3	119	4250	5.8
	Jun18		30	34.1	24.6	7.6	0.8	10.2	12	4.1	4.3	3.4	138	4410	3.3
	Oct18		29	29.6	21.3	7.5	0.9	4.8	9	4.7	5.1	3.6	128	3720	3.8
	Jan19		NA	NA	NA	NA	NA	NA	NA	NA	NA	NA	NA	NA	NA
**Fuliya1**	Sep17	Fuliya	29	23.9	17.2	7	0.8	NA	21.7	NA	3.7	NA	NA	3150	NA
	Jan18		28	24	17.3	7.1	0.6	9.7	12.5	4.6	4.8	3.1	80	3230	3.9
	Jun18		28	26.2	18.8	7.8	0.6	10.7	14.3	5	5.2	4.9	105	3460	7.9
	Oct18		27.5	23.2	16.7	7.4	0.7	5.4	10.5	3.9	5.7	2.9	74	3060	5.8
	Jan19		28	26.8	19.3	7.6	0.7	5.6	9.8	1.8	4	1.7	67	3380	0.7
**Ein7**	Sep17	Tabgha	25	37	24.4	7.4	1.2	NA	19.8	NA	2.8	NA	NA	4480	NA
	Jan18		23	11.3	7.5	7.9	1.9	35.5	54.2	6	6.2	5.7	34	1795	5.4

	Jun18		24	31.6	20.9	7.7	1.6	24.2	21.5	3.8	4.4	4.3	85	4030	18.9
	Oct18		25	37.3	24.6	7.2	0.8	12.6	16	4	4.4	3	100	4500	3.2
	Jan19		23	11.1	7.3	7.2	5.9	20.4	45.5	5.6	6.3	4.9	39	1775	0.2
**Nur**	Sep17	Tabgha	26	58.4	38.5	7.3	0.5	NA	17.4	NA	2	NA	NA	6660	NA
	Jan18		26	41.9	27.6	7.4	1.6	6.3	18.5	2.7	3	1.6	114	5070	12.6
	Jun18		27	56.1	37.1	7.3	1.2	18.7	19.9	2.6	2.9	2.5	157	6660	8.6
	Oct18		27	70.5	46.5	7	2.6	19.9	20	2.6	3.5	1.5	184	7850	8.7
	Jan19		27	60	39.6	7.1	3.6	17.6	25.8	3.2	3.2	1.3	145	6780	1.8
**Maayan1**	Sep17	Tabgha	29	37.6	24.8	7.4	1.2	NA	17	NA	2.2	NA	NA	4460	NA
	Jan18		23	24.9	16.5	7.7	3.8	6.4	20.6	3.6	3.7	3	58	3310	3.4
	Jun18		24	32.2	21.3	7.4	2	25.3	36	4.2	4.3	4.2	86	4170	16.9
	Oct18		25	38.6	25.5	7.2	27.8	15.8	26.9	4.2	4.3	2.8	102	4670	4.5
	Jan19		23.5	12.4	8.2	7.1	2.9	24.1	33.4	5.7	6.2	5.2	45	1927	3.8
**Gesher Hana**	Sep17	Tabgha	26	37.1	24.5	7.9	2.2	NA	18.3	NA	2.4	NA	NA	4430	NA
	Jan18		23	27.4	18.1	7.1	2.6	7.2	13.4	1.9	3.3	1.5	66	3500	9.9
	Jun18		25	31.6	20.9	8.2	1.4	18.2	24.6	4.3	4.3	4.3	82	4100	7.1
	Oct18		25	37.9	25.0	7.6	11.1	10.6	41.5	4	4.6	2.8	95	4530	7.7
	Jan19		22	11.2	7.4	7.8	5	32.2	43.2	3.9	6.3	3.6	36	1780	1.6
**Barbutim**	Sep17	Barbutim	26	29.6	19.5	7.5	1.3	NA	19.7	NA	0.6	NA	NA	3630	NA
	Jan18		24	28.7	18.9	7.4	2.3	10.3	19.5	0.9	0.9	0.6	66	3690	7
	Jun18		28.3	33.7	22.2	7.2	1.2	12.5	23.8	0.8	0.9	0.8	46	4350	5.4
	Oct18		27	34.9	23.0	7.2	0.9	11.4	12.4	1.1	1.2	0.8	88	4280	16
	Jan19		21.9	41.8	27.6	7	4.5	13	85.1	0.5	1	0.2	76	5180	1.5
**Tiberias Head**	Sep17	THS	59	520.2	291.3	6.5	8.8	NA	24.6	NA	9.4	NA	NA	46600	NA
	Jan18		56	487.2	272.9	6.1	0.7	8.7	8.7	2.1	3.6	0.05	584	45900	157
	Jun18		59	505.4	283.0	6.1	0.9	19.9	21.7	3.9	5.8	0.02	537	46600	253.4
	Oct18		58.5	519.8	291.1	6.4	0.8	12	12.6	7.5	9.9	0.02	761	46900	146.3
	Jan19		58	509.9	285.5	6.5	0.8	6.8	7	17.2	33.7	0.05	698	45700	86.2
**Tiberias Pump**	Sep17	THS	51	494.8	277.1	6.9	2.9	NA	27.9	NA	2.5	NA	NA	44900	NA
	Jan18		39	391	219.5	6.7	5.9	11.2	60.7	1.3	2.2	0.7	455	37600	6.6

	Jun18		42	402.8	225.6	7.2	1.7	13.3	20.6	3.8	4	0.3	407	37700	6.1
	Oct18		49	461.4	258.4	6.9	2.9	24.2	30.6	9.1	9.4	0.02	753	43000	25.6
	Jan19		43.5	499.4	279.7	7	7.4	13.4	24.8	14.5	28.5	0.1	636	44400	0.1
	Sep17		46	525.4	294.2	7	1.3	NA	20.6	NA	7.7	NA	NA	47900	NA
	Jan18		40	511.4	286.4	7	5.4	9	16	2	3.8	0.8	621	46800	15.3
**Tiberias Pool**	Jun18	THS	45	523.4	293.1	7	1.3	19	22.7	5.8	6.1	0.1	572	46800	5
	Oct18		44	544.6	305.0	6.9	1.3	11.3	16.3	6	8.3	0.02	813	48400	6
	Jan19		39	543.1	304.2	7.3	0.8	8.7	8.7	16.9	36.2	0.1	662	47500	10
	Sep17		27	128.3	69.3	7.4	17.7	NA	98.3	NA	2.6	NA	NA	14400	NA
	Jan18		22.1	122	65.9	7.3	6.1	95.4	130.2	3.3	3.7	1.2	542	13920	14.8
**Haon-Borehole**	Jun18	Haon-Borehole	26	118.1	63.8	7.3	12.1	71.6	80.2	2.7	3.5	0.6	460	13680	9.1
	Oct18		26	117.2	63.3	7.2	6.2	25.9	41.9	5.2	6	0.4	612	13410	25
	Jan19		25	254.7	137.5	6.8	15.2	13.4	75.1	4.5	4.9	0.1	573	25500	103.3

Sodium concentrations were calculated from Cl levels, based on published Na⁄Cl Molar ratios, characteristic of each spring cluster[9,10]. THS = Tiberias Hot Springs, Temp = temperature. Total and dissolved phosphorus (TP and TDP), total and dissolved nitrogen (TN and TDN).

Supplementary Table 2: Ranges of physicochemical characteristics for the studied springs sites surrounding lake Kinneret.

Station	Spring cluster	Temp (°C)	Cl (mM)	NA (mM)	Ph	Turbidity (NTU)	TP (mg/L)	TN (µg/L)	SO4 (mg/L)	Fe (µg/L)
**Fuliya5**	Fuliya	27.5-29	21.7-26.5	17.1-19.1	7.1-7.5	0.4-1.8	9.2-22.6	4.2-5.1	50-104	0.2-5.6
**Fuliya4**	Fuliya	23-30	25.4-29.7	18.3-21.4	7.3-7.6	1.2-5.2	11.4-151.7	3.7-6.8	95-133	3.7-9.9
**Fuliya3**	Fuliya	26-31	25.9-45.7	18.6-32.9	7.1-7.8	0.7-3.1	8.8-19.7	2.5-4.9	93-170	0.9-11.3
**Fuliya2**	Fuliya	29-30	29.1-34.1	20.9-24.6	7.2-7.6	0.9-1.3	9-20.6	3-5.1	119-138	3.3-5.8
**Fuliya1**	Fuliya	27.5-29	23.2-26.8	16.7-19.3	7-7.8	0.6-0.7	9.8-21.7	3.7-5.7	67-105	0.7-7.9
**En Sheva**	Tabgha	23-25	11.1-37.3	7.3-24.6	7.2-7.9	0.8-5.9	16-54.2	2.8-6.3	34-100	0.2-18.9
**Nur**	Tabgha	26-27	41.9-70.5	27.6-39.6	7-7.4	0.5-3.6	17.4-25.8	2-3.5	114-184	1.8-12.6
**Maayan1**	Tabgha	23-29	12.4-38.6	8.2-25.5	7.1-7.7	1.2-27.8	17-36	2.2-6.2	45-102	3.4-16.9
**Gesher Hana**	Tabgha	22-26	11.2-37.9	7.4-25	7.1-8.2	1.4-11.1	13.4-43.2	2.4-6.3	36-95	1.6-9.9
**Barbutim**	Tabgha	21.9-28.3	28.7-41.8	18.9-27.6	7-7.5	0.9-4.5	12.4-85.1	0.6-1.2	46-88	1.5-16
**Tiberias Head**	THS	56-59	487.2-520.2	272.9-291.3	6.1-6.5	0.7-8.8	7-24.6	3.6-33.7	537-761	86.2-253.4
**Tiberias Pump**	THS	39-51	391-499.4	219.5-279.7	6.7-7.2	1.7-7.4	24.8-60.7	2.2-28.5	407-753	0.1-25.6
**Tiberias Pool**	THS	39-46	511.4-544.6	286.4-305	6.9-7.3	0.8-5.4	8.7-22.7	3.8-36.2	572-813	5-15.3
**Haon-Borehole**	Haon-Borehole	22.1-27	118.1-254.7	63.3-137.5	6.8-7.3	6.1-17.7	41.9-130.2	2.6-6	542-612	9.1-103.3

Sodium concentrations were calculated from Cl levels, based on published Na ⁄ Cl Molar ratios, characteristic of each spring cluster[9,10]. For the complete physicochemical dataset see Supplementary Table 1. THS = Tiberias Hot Springs. Total and dissolved phosphorus (TP and TDP), total and dissolved nitrogen (TN and TDN).

Supplementary Table 3: Ranges of physicochemical characteristics representing springs clusters surrounding lake Kinneret.

Spring cluster[1]	Temperature (°C)	Na (mM)[2]	pH	Fe (µg/L)[3]	SO_4 (mg/L)	NO_3 (µg/L)[4]
THS	39-59	219.5-305	6.1-7.3	0.1-253.4*	407-813	0.02-0.8
Haon-Borehole	22.1-27	63.3-137.5	6.8-7.4	9.1-103.3	460-612	0.1-1.2
Tabgha	21.9-29	7.3-46.5	7-8.2	0.2-19.9	34-184	0.2-7.5**
Fuliya	23-31	15.6-32.9	7.8	0.2-11.3	50-170	1-4.9

[1]Samples from indicated locations were taken on Sep17, Jan18, Jun18, Oct18 and Jan19.

[2]Sodium concentrations were calculated from Cl levels, based on published Na ⁄ Cl Molar ratios, measured at the four spring clusters[40,42]. Ranges of physicochemical characteristics per station are presented in Table S2. For the complete physicochemical dataset see Table S1. THS = Tiberias Hot Springs.

[3] Fe levels for Tiberias-Head station were extremely high compared to other station in the THS cluster.

[4] NO_3 levels for Barbutim station were extremely low compared to other station in Tabgha cluster.

Supplementary Table 4: Loading scores of environmental variables included in the initial PCA analysis.

Parameter	PC1	PC2	PC1	PC2
	Water		Biofilm	
**Temperature**	-0.344	-0.207	-0.354	-0.041
**Ph**	0.301	-0.032	0.29	-0.01
**Turbidity**	-0.016	0.528	0.118	0.356
**Na**	-0.379	0.027	-0.357	0.003
**Cl**	-0.379	0.049	-0.356	0.03
**Conductivity**	-0.379	0.056	-0.357	0.034
**TP**	0.025	0.539	0.131	0.548
**TDP**	-0.029	0.492	0.052	0.586
**TN**	-0.188	-0.229	-0.20	0.28
**TDN**	-0.15	-0.11	-0.146	0.35
**NO_3^-**	0.326	-0.119	0.335	0.03
**SO_4**	-0.364	0.079	-0.351	-0.022
**Fe**	-0.24	0.058	-0.26	0.14

Values signify variable importance in explaining principal components (PC 1 and PC 2) of the initial PCA (Supplementary Fig. 2). Total and dissolved phosphorus (TP and TDP), total and dissolved nitrogen (TN and TDN).

Supplementary Table 5: Correlations between the physicochemical parameters comprising the initial PCA.

Water				Biofilm			
**Variable1**	**Variable2**	**Correlation**	**p. value**	**Variable1**	**Variable2**	**Correlation**	**p. value**
Ph	Turbidity	-0.099936853	0.4678704	Ph	Turbidity	0.113754873	0.4904903
Ph	Temp	-0.43716008	0.000846155	Ph	Temp	-0.504703915	0.001050867
Turbidity	Temp	-0.473796623	0.000258403	Turbidity	Temp	-0.554373838	0.000250321
Ph	TDP	-0.219455422	0.1074339	Ph	TDP	-0.050529087	0.7599987
Turbidity	TDP	0.418524936	0.001473143	Turbidity	TDP	0.231672743	0.1558656
Temp	TDP	-0.266190023	0.0494866	Temp	TDP	-0.260800607	0.1088023
Ph	TP	-0.113739289	0.4083353	Ph	TP	0.087779691	0.5951582
Turbidity	TP	0.699119674	2.90844E-09	Turbidity	TP	0.590280942	7.64263E-05
Temp	TP	-0.477632008	0.000226442	Temp	TP	-0.523097137	0.000633703
TDP	TP	0.643736022	1.14818E-07	TDP	TP	0.55161242	0.000272724
Ph	TN	-0.06331535	0.6460653	Ph	TN	-0.186531647	0.2555369
Turbidity	TN	-0.167111673	0.222668	Turbidity	TN	-0.209153969	0.2012996
Temp	TN	0.246984018	0.06908128	Temp	TN	0.157647009	0.3378181
TDP	TN	0.067479792	0.6244824	TDP	TN	0.246746671	0.1299443
TP	TN	-0.040429371	0.7694723	TP	TN	0.152997168	0.3524379
Ph	TDN	-0.070960759	0.6066779	Ph	TDN	-0.178141137	0.2779203
Turbidity	TDN	-0.149709565	0.2753009	Turbidity	TDN	-0.243128322	0.1358519
Temp	TDN	0.24588071	0.0703722	Temp	TDN	0.208476693	0.202796
TDP	TDN	0.084397778	0.5401218	TDP	TDN	0.207209034	0.2056175
TP	TDN	-0.077966591	0.5715317	TP	TDN	-0.013160559	0.9366214
TN	TDN	0.898664985	0	TN	TDN	0.804222374	6.96016E-10
Ph	Na	-0.602649397	1.12487E-06	Ph	Na	-0.39437518	0.01297858
Turbidity	Na	0.080266738	0.5602013	Turbidity	Na	-0.160565438	0.3288359
Temp	Na	0.572236684	5.00989E-06	Temp	Na	0.698098935	7.79386E-07
TDP	Na	0.186658888	0.1724007	TDP	Na	-0.030826404	0.8522161
TP	Na	0.031344315	0.8202899	TP	Na	-0.236519909	0.1471506
TN	Na	-0.015013836	0.9133657	TN	Na	-0.024936624	0.8802236
TDN	Na	0.027261424	0.8433823	TDN	Na	0.027622763	0.8674304

Ph	SO4	-0.530121793	3.14705E-05	Ph	SO4	-0.437632887	0.005333913
Turbidity	SO4	0.016415918	0.9053107	Turbidity	SO4	-0.298354847	0.06505593
Temp	SO4	0.633761102	2.06064E-07	Temp	SO4	0.796865702	1.28783E-09
TDP	SO4	0.045500469	0.7415051	TDP	SO4	-0.236014785	0.1480418
TP	SO4	-0.046433597	0.7363937	TP	SO4	-0.305324965	0.0587406
TN	SO4	0.210012629	0.1238189	TN	SO4	0.15112394	0.3584344
TDN	SO4	0.272323845	0.04428209	TDN	SO4	0.214618344	0.1895087
Na	SO4	0.886836351	0	Na	SO4	0.870675585	5.88418E-13
Ph	Fe	-0.327811762	0.01455748	Ph	Fe	-0.365454737	0.02215011
Turbidity	Fe	0.00050516	0.9970795	Turbidity	Fe	-0.064003243	0.6986874
Temp	Fe	0.169462735	0.2161347	Temp	Fe	0.317562309	0.04884093
TDP	Fe	0.143823752	0.2948299	TDP	Fe	0.073074392	0.6584214
TP	Fe	0.1092769	0.4270864	TP	Fe	0.084253168	0.6100863
TN	Fe	0.041263713	0.7648497	TN	Fe	0.137749418	0.4030123
TDN	Fe	0.016417695	0.9053005	TDN	Fe	0.068658231	0.6779143
Na	Fe	0.445679489	0.000649737	Na	Fe	0.450885611	0.00396791
SO4	Fe	0.479108035	0.000215135	SO4	Fe	0.47022787	0.002522126
Ph	NO_3^-	0.695887544	3.68632E-09	Ph	NO_3^-	0.571211585	0.000145984
Turbidity	NO_3^-	-0.098744273	0.4732244	Turbidity	NO_3^-	0.125455651	0.4466608
Temp	NO_3^-	-0.47080712	0.000286105	Temp	NO_3^-	-0.585835815	8.91935E-05
TDP	NO_3^-	-0.107380007	0.4352024	TDP	NO_3^-	0.21756962	0.1833475
TP	NO_3^-	-0.038158987	0.7820909	TP	NO_3^-	0.283956871	0.07979493
TN	NO_3^-	0.107863304	0.4331264	TN	NO_3^-	0.11929718	0.4694583
TDN	NO_3^-	0.164615886	0.2297524	TDN	NO_3^-	0.246038579	0.131085
Na	NO_3^-	-0.79875397	2.73115E-13	Na	NO_3^-	-0.68381801	1.5972E-06
SO4	NO_3^-	-0.636560466	1.75242E-07	SO4	NO_3^-	-0.567812491	0.000163156
Fe	NO_3^-	-0.403139661	0.002275186	Fe	NO_3^-	-0.365929006	0.02196473
Ph	Cl	-0.614707383	5.95455E-07	Ph	Cl	-0.403118048	0.01094153
Turbidity	Cl	0.108519581	0.4303163	Turbidity	Cl	-0.104570533	0.5263834
Temp	Cl	0.536743866	2.39572E-05	Temp	Cl	0.646654341	8.68381E-06
TDP	Cl	0.203387999	0.1363988	TDP	Cl	-0.01569223	0.9244616
TP	Cl	0.062810037	0.6487044	TP	Cl	-0.181799778	0.2680077
TN	Cl	-0.051846374	0.7069731	TN	Cl	-0.080591276	0.6257538

TDN	Cl	-0.009380017	0.9458113	TDN	Cl	-0.02247191	0.8919888
Na	Cl	0.994401029	0	Na	Cl	0.988226176	0
SO4	Cl	0.859281708	0	SO4	Cl	0.825285964	1.02545E-10
Fe	Cl	0.467644465	0.00031833	Fe	Cl	0.478331347	0.002069651
Nitrate	Cl	-0.817009163	2.79776E-14	Nitrate	Cl	-0.703619359	5.84055E-07
Ph	Conductivity	-0.616339498	5.45213E-07	Ph	Conductivity	-0.405072904	0.01052536
Turbidity	Conductivity	0.099471434	0.469956	Turbidity	Conductivity	-0.117146763	0.4775614
Temp	Conductivity	0.544200219	1.74996E-05	Temp	Conductivity	0.65694847	5.56023E-06
TDP	Conductivity	0.21089	0.122221	TDP	Conductivity	0.005012404	0.9758404
TP	Conductivity	0.045406166	0.7420223	TP	Conductivity	-0.207502279	0.2049624
TN	Conductivity	-0.051741868	0.7075373	TN	Conductivity	-0.075443038	0.6480561
TDN	Conductivity	0.006241769	0.9639261	TDN	Conductivity	0.011845702	0.9429426
Na	Conductivity	0.992503566	0	Na	Conductivity	0.98370353	0
SO4	Conductivity	0.863312467	0	SO4	Conductivity	0.838007496	2.8388E-11
Fe	Conductivity	0.469825177	0.000295778	Fe	Conductivity	0.494885568	0.001361044
Nitrate	Conductivity	-0.79927831	2.56684E-13	Nitrate	Conductivity	-0.664978989	3.88048E-06
Cl	Conductivity	0.995995385	0	Cl	Conductivity	0.990888864	0

Total and dissolved phosphorus (TP and TDP), total and dissolved nitrogen (TN and TDN).

Supplementary Table 6: Loading scores of environmental variables included in the final PCA analysis.

Parameter	PC1	PC2	PC1	PC2
	Water		Biofilm	
**Temperature**	-0.418	-0.254	-0.43	0.014
**Ph**	0.39	-0.006	0.36	0.177
**Na**	-0.454	0.034	-0.422	-0.076
**Turbidity**	-0.007	0.678	0.154	-0.67
**TP**	0.035	0.668	0.17	-0.673
**SO_4**	-0.431	0.125	-0.415	-0.048
**NO_3^-**	0.412	-0.099	0.403	0.085
**Fe**	-0.319	0.036	-0.335	-0.218

Values signify variable importance in explaining principal components (PC1 and PC2) of the final PCA (Fig. 2D and 2E). The following environmental variables were included in the initial PCA analysis: Temperature, Ph, Na, turbidity, TP, SO_4, NO_3^- and Fe, and represent a subset of the environmental variables evaluated in this study. For the full list see Supplementary Fig. 2 and Supplementary Table 3.

Supplementary Table 7: Correlations between the selected physicochemical parameters included in the final PCA.

Water				Biofilm			
**Variable1**	**Variable2**	**Correlation**	**p. value**	**Variable1**	**Variable2**	**Correlation**	**p. value**
Ph	Turbidity	0.11375487	4.904903e-01	Ph	Turbidity	-0.0999368529	4.678704e-01
Ph	Temp	-0.50470391	1.050867e-03	Ph	Temp	-0.4371600801	8.461550e-04
Turbidity	Temp	-0.55437384	2.503211e-04	Turbidity	Temp	-0.4737966232	2.584028e-04
Ph	TP	0.08777969	5.951582e-01	Ph	TP	-0.1137392888	4.083353e-01
Turbidity	TP	0.59028094	7.642633e-05	Turbidity	TP	0.6991196738	2.908443e-09
Temp	TP	-0.52309714	6.337034e-04	Temp	TP	-0.4776320077	2.264422e-04
Ph	Na	-0.39437518	1.297858e-02	Ph	Na	-0.6026493969	1.124866e-06
Turbidity	Na	-0.16056544	3.288359e-01	Turbidity	Na	0.0802667382	5.602013e-01
Temp	Na	0.69809894	7.793855e-07	Temp	Na	0.5722366840	5.009888e-06
TP	Na	-0.23651991	1.471506e-01	TP	Na	0.0313443148	8.202899e-01
Ph	SO4	-0.43763289	5.333913e-03	Ph	SO4	-0.5301217932	3.147051e-05
Turbidity	SO4	-0.29835485	6.505593e-02	Turbidity	SO4	0.0164159180	9.053107e-01
Temp	SO4	0.79686570	1.287825e-09	Temp	SO4	0.6337611023	2.060640e-07
TP	SO4	-0.30532496	5.874060e-02	TP	SO4	-0.0464335967	7.363937e-01
Na	SO4	0.87067559	5.884182e-13	Na	SO4	0.8868363514	0.000000e+00
Ph	Fe	-0.36545474	2.215011e-02	Ph	Fe	-0.3278117618	1.455748e-02
Turbidity	Fe	-0.06400324	6.986874e-01	Turbidity	Fe	0.0005051598	9.970795e-01
Temp	Fe	0.31756231	4.884093e-02	Temp	Fe	0.1694627348	2.161347e-01
TP	Fe	0.08425317	6.100863e-01	TP	Fe	0.1092769004	4.270864e-01
Na	Fe	0.45088561	3.967910e-03	Na	Fe	0.4456794885	6.497374e-04
SO4	Fe	0.47022787	2.522126e-03	SO4	Fe	0.4791080348	2.151345e-04
Ph	NO_3^-	0.57121158	1.459836e-04	Ph	NO_3^-	0.6958875436	3.686317e-09
Turbidity	NO_3^-	0.12545565	4.466608e-01	Turbidity	NO_3^-	-0.0987442727	4.732244e-01
Temp	NO_3^-	-0.58583581	8.919345e-05	Temp	NO_3^-	-0.4708071203	2.861053e-04
TP	NO_3^-	0.28395687	7.979493e-02	TP	NO_3^-	-0.0381589872	7.820909e-01
Na	NO_3^-	-0.68381801	1.597197e-06	Na	NO_3^-	-0.7987539697	2.731149e-13
SO4	NO_3^-	-0.56781249	1.631559e-04	SO4	NO_3^-	-0.6365604656	1.752422e-07
Fe	NO_3^-	-0.36592901	2.196473e-02	Fe	NO_3^-	-0.4031396610	2.275186e-03

Supplementary Table 8: K-means clustering summery table for qPCR heatmap (Fig. 2C).

**Cluster means:**	Sep17	Jan18	Jun18	Oct18	Jan19
**Cluster 1**	10948.66	6787.029	2280.894	7645.371	12661.6
**Cluster 2**	73459.07	62483.33	23772.23	29122.97	104775.2
**Cluster 3**	123667	846333	371500	255123	792
**Cluster 4**	8033.6	6220	20325	17215.7	278586.1

**Clustering vector:**				
	Fuliya5-water	Fuliya1-water	Ein7-water	Fuliya4-water
	1	1	1	1
	Gesher Hana-water	Maayan1-water	Barbutim-water	Fuliya3-water
	1	1	1	1
	Nur-water	Haon-Borehole-water	Tiberias Pump-water	Tiberias Head-water
	1	2	3	1
	Tiberias Pool-water	Fuliya5-biofilm	Fuliya1-biofilm	Ein7-biofilm
	2	1	1	1
	Fuliya4-biofilm	Gesher Hana-biofilm	Maayan1-biofilm	Fuliya3-biofilm
	1	1	1	1
	Tiberias Head-biofilm	Tiberias Pool-biofilm		
	2	4		

**Within cluster sum of squares by cluster**	[1] 17697989617 13046054205 0 0<br>(between_SS / total_SS = 96.9 %)

K-means clustering with 4 clusters of sizes: 17, 3, 1, 1. Optimal number of clusters was determined in r using the package factoextra.

Supplementary Table 9: Kruskal-Wallis and post hoc via Wilcox test for *Legionella* spp. qPCR levels, between the different spring clusters and collection dates.

Kruskal-Wallis test				Wilcox tests post hoc for multiple comparisons							
**Comparison**	**H statistic**	**DF**	**p. value**	**group1**	**group2**	**n1**	**n2**	**statistic**	**p. value**	**p.adj**	**p.adj.signif (BH)**
**Spring cluster water**	34.91	3	1.27e-07	THS	Haon-Borehole	10	5	39	0.099	0.099	ns
				THS	Tabgha	10	25	226	6.62E-05	0.000132	***
				THS	Fuliya	10	24	227	5.58E-06	3.35E-05	****
				Haon-Borehole	Tabgha	5	25	106	0.013	0.016	*
				Haon-Borehole	Fuliya	5	24	117	0.000118	0.000177	***
				Tabgha	Fuliya	25	24	501	2.52E-05	7.56E-05	****
**Spring cluster biofilm**	12.68	2	0.0017	THS	Tabgha	10	15	125	0.004	0.007	**
				THS	Fuliya	10	25	212	0.000212	0.000636	***
				Tabgha	Fuliya	15	25	198	0.618	0.618	ns
**Collection date water**	4.77	4	0.31	-	-	-	-	-	-	-	-
**Collection date biofilm**	14.68	4	0.005	Sep17	Jan18	10	10	47	0.853	0.853	ns
				Sep17	Jun18	10	10	63	0.353	0.441	ns
				Sep17	Oct18	10	10	53	0.853	0.853	ns
				Sep17	Jan19	10	10	10	0.003	0.014	*
				Jan18	Jun18	10	10	74	0.075	0.151	ns
				Jan18	Oct18	10	10	64	0.315	0.441	ns
				Jan18	Jan19	10	10	15	0.013	0.033	*
				Jun18	Oct18	10	10	36	0.315	0.441	ns
				Jun18	Jan19	10	10	11	0.004	0.014	*
				Oct18	Jan19	10	10	8	0.001	0.014	*

To count for multiple comparisons, p. values were adjusted according to the Benjamini-Hochberg (BH) method. Water and biofilm samples were analyzed separately, from one of the following spring clusters: Tiberias Hot Springs (THS), Haon-Borehole, Tabgha and Fuliya. Biofilm was not available for Haon-Borehole. H statistic = test value; ns = non-significant; significance level; * $p \leq 0.05$; ** $p \leq 0.01$; *** $p \leq 0.001$, **** $p \leq 0.0001$.

Supplementary Table 10: Feature and sequence counts for all primer sets used in the study.

Primer set	Target amplicon	Samples	Read count (post DADA2)	ASV count (post DADA2)	Read count (Minimal threshold)	ASV count (Minimal threshold)	Read count	Maximum frequency per sample	Median frequency per sample	1st quantile frequency	ASV count post filtering
**Lgsp17F-Lgsp28R**	16S V3-V4 *Legionella* spp.	n=69	3,763,976	9,968	3,402,219	2,475	1,439,806	60,396	20,284	4,072	1,079
**341F-805R**	16S V3-V4 Bacteria universal	n=69	3,879,696	47,078	3,345,194	7,967					
**EUK1391F-EUK1510R**	18S V9 (Protozoa hosts)	n=74	5,992,098	19,299	5,650,504	6,917	474,320	7,057	3,037	1,125	400

Minimal threshold frequency filter of 20 reads across all samples, in min 2 samples, was employed. Quality-filtering was performed using DADA2.

Supplementary Table 11: Kruskal-Wallis test results of 16S NGS alpha diversity between the two sequencing centers.

Kruskal-Wallis test				
**Comparison**	**Alpha index**	**H statistic**	**DF**	**p. value**
**MrDNA and UIC sequencing centers**	**Shannon entropy**	0.02	1	0.9
	**Faith's PD**	0.89	1	0.34
	**Pielou's evenness**	1.41	1	0.235
	**Observed OTUs**	2.03	1	0.15

Water and biofilm samples were analyzed separately and taken from one of the following spring clusters: Tiberias Hot Springs, Haon-Borehole, Tabgha and Fuliya. Biofilm was not available for Haon-Borehole. H statistic = test value. Samples collected form 2017 and 2018-2019 were sent to MrDNA (n=12) and UIC (n=48) sequencing centers, respectively.

Supplementary Table 12: PERMANOVA, PERMDISP and pairwise post hoc results of 16S NGS beta between the two sequencing centers, collection years and water vs. biofilm.

Beta metrics	test	Group 1	Group 2	Group 3	Sample size	Permutations	pseudo-F	p-value	q-value
**Jaccard**	PERMANOVA	MrDNA	UIC	-	60	999	1.48969	0.022	-
**Bray-Curtis**	PERMANOVA	MrDNA	UIC	-	60	999	1.7751	0.015	-
**Jaccard**	PERMDISP	MrDNA	UIC	-	60	999	20.6324	0.028	-
**Bray-Curtis**	PERMDISP	MrDNA	UIC	-	60	999	10.6238	0.092	-
**Jaccard**	PERMANOVA	Water	Biofilm	-	60	999	1.60284	0.015	-
**Bray-Curtis**	PERMANOVA	Water	Biofilm	-	60	999	1.63276	0.02	-
**Jaccard**	PERMDISP	Water	Biofilm	-	60	999	6.14238	0.04	-
**Bray-Curtis**	PERMDISP	Water	Biofilm	-	60	999	4.56573	0.059	-
**Jaccard**	PERMANOVA	2017	2018	2019	60	999	1.27134	0.034	-
**Bray-Curtis**	PERMANOVA	2017	2018	2019	60	999	1.4809	0.016	-
**Jaccard**	PERMDISP	2017	2018	2019	60	999	5.05457	0.106	-
**Bray-Curtis**	PERMDISP	2017	2018	2019	60	999	2.70939	0.248	-
**Jaccard**	PERMANOVA	2017	2018	-	44	999	1.312847	0.084	0.126
**Jaccard**	PERMANOVA	2017	2019	-	28	999	1.60573	0.01	0.03
**Jaccard**	PERMANOVA	2018	2019	-	48	999	1.041877	0.312	0.312
**Bray-Curtis**	PERMANOVA	2017	2018	-	44	999	1.532478	0.021	0.0315
**Bray-Curtis**	PERMANOVA	2017	2019	-	28	999	1.941214	0.002	0.006
**Bray-Curtis**	PERMANOVA	2018	2019	-	48	999	1.171635	0.214	0.214

Supplementary Table 13: Kruskal-Wallis and post hoc via Wilcox tests of 16S NGS alpha diversity between the different spring clusters and collection dates.

Kruskal-Wallis test					Wilcox tests post hoc for multiple comparisons							
**Comparison**	**Alpha index**	**H statistic**	**DF**	**p. value**	**Group1**	**group2**	**n1**	**n2**	**statistic**	**p. value**	**p.adj**	**p.adj.signif (BH)**
**Springs cluster water**	**Shannon entropy**	23.12	3	3.81e-05	Fuliya	Haon-Borehole	4	4	16	0.029	0.043	*
					Fuliya	Tabgha	4	19	38	**1**	1	ns
					Fuliya	THS	4	10	40	0.002	0.004	**
					Haon-Borehole	Tabgha	4	19	0	2.26e-04	6.78e-04	***
					Haon-Borehole	THS	4	10	7	0.07	0.09	ns
					Tabgha	THS	19	10	180	1.39e-05	8.34e-05	****
	**Faith's PD**	23.8	3	2.75e-05	Fuliya	Haon-Borehole	4	4	16	0.029	0.034	*
					Fuliya	Tabgha	4	19	37	0.97	0.96	ns
					Fuliya	THS	4	10	40	0.002	0.004	**
					Haon-Borehole	Tabgha	4	19	0	2.26e-04	6.78e-04	***
					Haon-Borehole	THS	4	10	3	0.014	0.021	*
					Tabgha	THS	19	10	181	9.69e-06	5.81e-05	****
	**Pielou's evenness**	7.96	3	0.046	Fuliya	Haon-Borehole	4	4	14	0.114	0.171	ns
					Fuliya	Tabgha	4	19	45	0.611	0.733	ns
					Fuliya	THS	4	10	33	0.076	0.171	ns
					Haon-Borehole	Tabgha	4	19	17	0.097	0.171	ns
					Haon-Borehole	THS	4	10	17	0.733	0.733	ns
					Tabgha	THS	19	10	143	0.027	0.164	ns
	**Observed OTUs**	23.11	3	3.83e-05	Fuliya	Haon-Borehole	4	4	16.0	0.029	0.034	*
					Fuliya	Tabgha	4	19	34.5	0.81	0.808	ns
					Fuliya	THS	4	10	39.0	0.009	0.016	*
					Haon-Borehole	Tabgha	4	19	0.0	0.002	0.007	**
					Haon-Borehole	THS	4	10	1.5	0.011	0.016	*
					Tabgha	THS	19	10	178.5	1.39e-04	8.34e-04	***
**Springs cluster biofilm**	**Shannon entropy**	13.8	2	9.6e-04	Fuliya	Tabgha	4	12	34	0.26	0.262	ns
					Fuliya	THS	4	8	32	0.004	0.006	**
					Tabgha	THS	12	8	91	3.02e-04	9.06e-04	***
	**Faith PD**	15.9	2	3.53e-05	Fuliya	Tabgha	4	12	33	0.316	0.31	ns
					Fuliya	THS	4	8	32	0.004	0.006	**
					Tabgha	THS	12	8	96	1.59e-05	4.77e-05	****

	**Pielou evenness**	6.79	2	0.033	Fuliya	Tabgha	4	12	22	0.862	0.862	ns
					Fuliya	THS	4	8	26	0.024	0.036	*
					Tabgha	THS	12	8	69	0.022	0.036	*
	**Observed OTUs**	14.67	2	6.51e-05	Fuliya	Tabgha	4	12	33.5	0.27	0.275	ns
					Fuliya	THS	4	8	32.0	0.008	0.012	*
					Tabgha	THS	12	8	93.0	5.91e-04	0.002	**
**Collection date Water**	**Shannon entropy**	2.79	4	0.59	-	-	-	-	-	-	-	-
	**Faith PD**	5.2	4	0.26								
	**Pielou evenness**	3.05	4	0.55								
	**Observed OTUs**	4.9	4	0.3								
**Collection date biofilm**	**Shannon entropy**	6.03	4	0.2	-	-	-	-	-	-	-	-
	**Faith PD**	6.86	4	0.14								
	**Pielou evenness**	6.4	4	0.17								
	**Observed OTUs**	7.45	4	0.11								
**Environment (Water vs. Biofilm)**	**Shannon entropy**	2.09	1	0.15	-	-	-	-	-	-	-	-
	**Faith PD**	2.14	1	0.14								
	**Pielou evenness**	0.36	1	0.55								
	**Observed OTUs**	2.4	1	0.12								

To count for multiple comparisons, p. values were adjusted according to the Benjamini-Hochberg (BH) method. Water and biofilm samples were analyzed separately and taken from one of the following spring clusters: Tiberias Hot Springs (THS), Haon-Borehole, Tabgha and Fuliya. Biofilm was not available for Haon-Borehole. Collection dates were Sep17, Jan18, Jun18, Oct18 and Jan19. H statistic = test value; ns = non-significant; significance level; * p ≤ 0.05; ** p ≤ 0.01; *** p ≤ 0.001, **** p ≤ 0.0001.

Supplementary Table 14: PERMANOVA and PERMDISP results of 16S NGS beta diversity between the different spring clusters.

Beta metrics	environment	Tested variable	test	number of groups	Sample size	Permutations	pseudo-F	p-value
**Bray Curtis**	Water	Spring Cluster	PERMANOVA	4	37	999	3.26	0.001
**Bray Curtis**	Biofilm	Spring Cluster	PERMANOVA	3	24	999	3.299	0.001
**Jaccard**	Water	Spring Cluster	PERMANOVA	4	37	999	3.38757	0.001
**Jaccard**	Biofilm	Spring Cluster	PERMANOVA	3	24	999	2.811	0.001
**Bray Curtis**	Water	Spring Cluster	PERMDISP	4	37	999	7.4722	0.235
**Bray Curtis**	Biofilm	Spring Cluster	PERMDISP	3	24	999	2.466	0.304
**Jaccard**	Water	Spring Cluster	PERMDISP	4	37	999	28.7388	0.006
**Jaccard**	Biofilm	Spring Cluster	PERMDISP	3	24	999	4.8599	0.253

Water and biofilm samples were analyzed separately and taken from one of the following spring clusters: Tiberias Hot Springs (THS), Haon-Borehole, Tabgha and Fuliya. Biofilm was not available for Haon-Borehole

Supplementary Table 15: PERMANOVA and PERMDISP post hoc pairwise comparison results of 16S NGS beta diversity between the different spring clusters

Beta metric	Environment	test	Group 1	Group 2	Sample size	Permutations	pseudo-F	p-value	q-value
**Bray-Curtis**	Water	PERMANOVA	Fuliya	THS	14	999	2.923399	0.001	0.002
			Fuliya	Haon-Borehole	8	999	4.893444	0.033	0.033
			Fuliya	Tabgha	23	999	1.896062	0.011	0.0132
			THS	Haon-Borehole	14	999	4.032063	0.005	0.0075
			THS	Tabgha	29	999	3.563341	0.001	0.002
			Haon-Borehole	Tabgha	23	999	3.825626	0.001	0.002
**Jaccard**	Water	PERMANOVA	Fuliya	THS	14	999	2.870558	0.002	0.003
			Fuliya	Haon-Borehole	8	999	6.505273	0.023	0.023
			Fuliya	Tabgha	23	999	1.991533	0.003	0.0036
			THS	Haon-Borehole	14	999	4.347704	0.002	0.003
			THS	Tabgha	29	999	3.301404	0.001	0.003
			Haon-Borehole	Tabgha	23	999	4.359156	0.001	0.003
**Bray-Curtis**	Biofilm	PERMANOVA	Fuliya	THS	12	999	4.095935	0.003	0.0045
			Fuliya	Tabgha	16	999	1.551239	0.021	0.021
			THS	Tabgha	20	999	4.447538	0.001	0.003
**Jaccard**	Biofilm	PERMANOVA	Fuliya	THS	12	999	3.240029	0.002	0.003
			Fuliya	Tabgha	16	999	1.739115	0.01	0.01
			THS	Tabgha	20	999	3.455162	0.001	0.003
**Bray-Curtis**	Water	PERMDISP	Fuliya	THS	14	999	0.703293	0.466	0.466
			Fuliya	Haon-Borehole	8	999	1.644185	0.02	0.04
			Fuliya	Tabgha	23	999	28.00092	0.01	0.036
			THS	Haon-Borehole	14	999	4.885427	0.012	0.036
			THS	Tabgha	29	999	2.185118	0.251	0.3012
			Haon-Borehole	Tabgha	23	999	24.1705	0.032	0.048
**Jaccard**	Water	PERMDISP	Fuliya	THS	14	999	3.687516	0.227	0.2724
			Fuliya	Haon-Borehole	8	999	7.298303	0.038	0.057

			Fuliya	Tabgha	23	999	75.84286	0.018	0.036
			THS	Haon-Borehole	14	999	22.32694	0.001	0.003
			THS	Tabgha	29	999	2.858051	0.406	0.406
			Haon-Borehole	Tabgha	23	999	124.5332	0.001	0.003
**Bray-Curtis**	Biofilm	PERMDISP	Fuliya	THS	12	999	0.114363	0.647	0.647
			Fuliya	Tabgha	16	999	13.08487	0.073	0.1095
			THS	Tabgha	20	999	4.095962	0.01	0.03
**Jaccard**	Biofilm	PERMDISP	Fuliya	THS	12	999	1.1827	0.42	0.42
			Fuliya	Tabgha	16	999	16.00572	0.085	0.1515
			THS	Tabgha	20	999	3.663031	0.101	0.1515

Water and biofilm samples were analyzed separately and taken from one of the following spring clusters: Tiberias Hot Springs (THS), Haon-Borehole, Tabgha and Fuliya. Biofilm was not available for Haon-Borehole.

Supplementary Table 16: ADONIS test results of 16S NGS beta diversity with selected environmental variables.

Beta index	Environment	Variable	df	Sums Of Sqs	Mean Sqs	F.Model	R2	Pr(>F)
**Jaccard**	**Water**	Na	1	1.273404	1.273404	3.101136	0.095587	0.001
		temperature	1	0.593475	0.593475	1.445298	0.044549	0.026
		pH	1	0.503856	0.503856	1.227046	0.037821	0.161
		Fe	1	0.685619	0.685619	1.669697	0.051465	0.01
		Residuals	25	10.26562	0.410625	NA	0.770578	NA
		Total	29	13.32198	NA	NA	1	NA
**Bray Curtis**	**Water**	Na	1	1.404609	1.404609	3.515347	0.105799	0.001
		temperature	1	0.620906	0.620906	1.553954	0.046768	0.028
		pH	1	0.456515	0.456515	1.14253	0.034386	0.259
		Fe	1	0.805013	0.805013	2.014724	0.060636	0.003
		Residuals	25	9.989124	0.399565	NA	0.75241	NA
		Total	29	13.27617	NA	NA	1	NA
**Jaccard**	**Biofilm**	Na	1	1.322338	1.322338	3.288235	0.163798	0.001
		temperature	1	0.522176	0.522176	1.298485	0.064682	0.088
		pH	1	0.388431	0.388431	0.965905	0.048115	0.466
		Fe	1	0.210044	0.210044	0.522312	0.026018	0.981
		Residuals	14	5.629992	0.402142	NA	0.697387	NA
		Total	18	8.072981	NA	NA	1	NA
**Bray Curtis**	**Biofilm**	Na	1	1.700805	1.700805	4.317189	0.215244	0.001
		temperature	1	0.246273	0.246273	0.625119	0.031167	0.93
		pH	1	0.367251	0.367251	0.9322	0.046477	0.523
		Fe	1	0.071976	0.071976	0.182697	0.009109	0.98
		Residuals	14	5.515456	0.393961	NA	0.698004	NA
		Total	18	7.90176	NA	NA	1	NA

Variables were chosen based on *Legionella* spp. known factors influencing growth, on the principal component analysis (PCA, Fig. 2D and 2E, Supplementary Table 5) and on the linear mixed effect models (LMM, see results section). Water and biofilm samples were analyzed separately and taken from one of the following spring clusters: Tiberias Hot Springs (THS), Haon-Borehole, Tabgha and Fuliya. Biofilm was not available for Haon-Borehole. The analysis was performed with 999 permutations.

Supplementary Table 17: Kruskal-Wallis test results of 18S NGS alpha diversity between the two sequencing centers

Kruskal-Wallis test				
**Comparison**	**Alpha index**	**H statistic**	**DF**	**p. value**
**MrDNA and UIC sequencing centers**	**Shannon entropy**	0.0004	1	0.983
	**Faith PD**	0.5	1	0.479
	**Pielou evenness**	0.8	1	0.371
	**Observed OTUs**	0.035	1	0.851

Water and biofilm samples were analyzed separately and taken from one of the following spring clusters: Tiberias Hot Springs (THS), Haon-Borehole, Tabgha and Fuliya. Biofilm was not available for Haon-Borehole. H statistic = test value. Samples collected form 2017 and 2018-2019 were sent to MrDNA (n=12) and UIC (n=42) sequencing centers, respectively.

Supplementary Table 18: PERMANOVA and PERMDISP results of 18S NGS beta diversity between the two sequencing centers and collection years

Beta metrix	test	Group 1	Group 2	Sample size	Permutations	pseudo-F	p-value
**Jaccard**	PERMANOVA	MrDNA	UIC	54	999	1.1314	0.213
**Bray-Curtis**	PERMANOVA	MrDNA	UIC	54	999	0.906203	0.597
**Jaccard**	PERMDISP	MrDNA	UIC	54	999	5.398834	0.313
**Bray-Curtis**	PERMDISP	MrDNA	UIC	54	999	3.391412	0.306

Prior to the analysis, sequences were filtered to only retain Amoebozoa, Ciliophora and Percolozoa, as potential *Legionella* spp. hosts. Samples were collected form 2017 and 2018-2019 were sent to MrDNA (n=12) and UIC (n=42) sequencing centers, respectively.

Supplementary Table 19: Kruskal-Wallis and post hoc via Wilcox tests of 18S NGS alpha diversity between the different spring clusters and collection dates.

Kruskal-Wallis test					Wilcox tests post hoc for multiple comparisons							
**Comparison**	**Alpha index**	**H**	**DF**	**p. value**	**group1**	**group2**	**n1**	**n2**	**statistic**	**p. value**	**p.adj**	**p.adj.sig**
**Springs cluster water**	Shannon entropy	15.469	3	0.0014	Fuliya	Haon-Borehole	4	4	16	0.029	0.034	*
					Fuliya	Tabgha	4	19	48	0.456	0.456	ns
					Fuliya	THS	4	8	31	0.008	0.016	*
					Haon-Borehole	Tabgha	4	19	4	0.003	0.012	*
					Haon-Borehole	THS	4	8	0	0.004	0.012	*
					Tabgha	THS	19	8	122	0.013	0.02	*
	Faith PD	18.109	3	4.1e-4	THS	Haon-Borehole	8	4	26	0.109	0.131	ns
					THS	Tabgha	8	19	16	7.16e-4	0.002	**
					THS	Fuliya	8	4	3	0.028	0.043	*
					Haon-Borehole	Tabgha	4	19	0	2.26e-4	0.001	**
					Haon-Borehole	Fuliya	4	4	0	0.029	0.043	*
					Tabgha	Fuliya	19	4	34	0.785	0.785	ns
	Pielou evenness	8.036	3	0.045	Fuliya	Haon-Borehole	4	4	16	0.029	0.057	ns
					Fuliya	Tabgha	4	19	42	0.785	0.815	ns
					Fuliya	THS	4	8	19	0.683	0.815	ns
					Haon-Borehole	Tabgha	4	19	8	0.012	0.036	*
					Haon-Borehole	THS	4	8	0	0.004	0.024	*
					Tabgha	THS	19	8	81	0.815	0.815	ns
	Observed OTUs	17.74	3	4.97e-4	Fuliya	Haon-Borehole	4	4	16	0.029	0.044	*
					Fuliya	Tabgha	4	19	48	0.441	0.441	ns
					Fuliya	THS	4	8	30	0.016	0.032	*
					Haon-Borehole	Tabgha	4	19	0	0.002	0.01	**
					Haon-Borehole	THS	4	8	6	0.106	0.127	ns
					Tabgha	THS	19	10	178.5	1.3e-4	8.3e-4	***
**Springs cluster biofilm**	Shannon entropy	13.2	2	0.0014	Fuliya	Tabgha	3	9	1	0.018	0.027	*
					Fuliya	THS	3	6	16	0.095	0.095	ns
					Tabgha	THS	9	6	54	0.0004	0.001	**
	Faith PD	12.25	2	0.0022	Fuliya	Tabgha	3	9	6	0.209	0.209	ns

					Fuliya	THS	3	6	18	0.024	0.036	*
					Tabgha	THS	9	6	54	0.0004	0.001	**
	Pielou evenness	4.58	2	0.1	Fuliya	Tabgha	3	9	2	0.036	0.109	ns
					Fuliya	THS	3	6	7	0.714	0.714	ns
					Tabgha	THS	9	6	38	0.224	0.336	ns
	Observed OTUs	12.28	2	0.002	Fuliya	Tabgha	3	9	6	0.195	0.195	ns
					Fuliya	THS	3	6	18	0.026	0.039	*
					Tabgha	THS	9	6	54	0.002	0.005	**
**Collection date Water**	Shannon	0.84	3	0.83	-	-	-	-	-	-	-	-
	Faith PD	0.69	3	0.87								
	Pielou	0.62	3	0.62								
	Observed	1.59	3	0.65								
**Collection date biofilm**	Shannon	0.6	3	0.89	-	-	-	-	-	-	-	-
	Faith PD	1.65	3	0.65								
	Pielou	1.44	3	0.7								
	Observed	1.25	3	0.74								
**Environment (water vs. biofilm)**	Shannon	2.09	1	0.15	-	-	-	-	-	-	-	-
	Faith PD	2.14	1	0.14								
	Pielou	0.36	1	0.55								
	Observed	2.4	1	0.12								

To account for multiple comparisons, p. values were adjusted according to the Benjamini-Hochberg (BH) method. Water and biofilm samples were analyzed separately and taken from one of the following spring clusters: Tiberias Hot Springs (THS), Haon-Borehole, Tabgha and Fuliya. Biofilm was not available for Haon-Borehole. Collection dates were Sep17, Jan18, Jun18, Oct18 and Jan19. H statistic = test value; ns = non-significant; significance level; * p ≤ 0.05; ** p ≤ 0.01; *** p ≤ 0.001, **** p ≤ 0.0001.

Supplementary Table 20: PERMANOVA and PERMDISP results of 18S NGS beta diversity between the different spring clusters.

Beta metrix	environment	Tested variable	test	number of groups	Sample size	Permutations	pseudo-F	p-value
**Bray Curtis**	Water	Spring Cluster	PERMANOVA	4	35	999	3.40123	0.001
**Bray Curtis**	Biofilm	Spring Cluster	PERMANOVA	3	19	999	4.18123	0.001
**Jaccard**	Water	Spring Cluster	PERMANOVA	4	35	999	3.28015	0.001
**Jaccard**	Biofilm	Spring Cluster	PERMANOVA	3	19	999	3.99162	0.001
**Bray Curtis**	Water	Spring Cluster	PERMDISP	4	35	999	6.11979	0.066
**Bray Curtis**	Biofilm	Spring Cluster	PERMDISP	3	19	999	11.2769	0.016
**Jaccard**	Water	Spring Cluster	PERMDISP	4	35	999	10.2081	0.07
**Jaccard**	Biofilm	Spring Cluster	PERMDISP	3	19	999	5.17071	0.039

Water and biofilm samples were analyzed separately and taken from one of the following spring clusters: Tiberias Hot Springs (THS), Haon-Borehole, Tabgha and Fuliya. Biofilm was not available for Haon-Borehole.

Supplementary Table 21: PERMANOVA and PERMDISP post hoc pairwise comparison results of 18S NGS beta diversity between the different spring clusters.

Beta metrix	Environment	test	Group 1	Group 2	Sample size	Permutations	pseudo-F	p-value	q-value
**Bray-Curtis**	Water	PERMANOVA	Fuliya	THS	12	999	2.698505	0.006	0.009
			Fuliya	Haon-Borehole	8	999	4.244803	0.028	0.0336
			Fuliya	Tabgha	23	999	1.482509	0.058	0.058
			THS	Haon-Borehole	12	999	4.32882	0.003	0.006
			THS	Tabgha	27	999	4.028105	0.001	0.003
			Haon-Borehole	Tabgha	23	999	4.496002	0.001	0.003
**Jaccard**	Water	PERMANOVA	Fuliya	THS	12	999	2.901325	0.002	0.004
			Fuliya	Haon-Borehole	8	999	4.191961	0.024	0.0288
			Fuliya	Tabgha	23	999	1.373111	0.045	0.045
			THS	Haon-Borehole	12	999	4.26854	0.004	0.006
			THS	Tabgha	27	999	3.953134	0.001	0.003
			Haon-Borehole	Tabgha	23	999	4.082117	0.001	0.003
**Bray-Curtis**	Biofilm	PERMANOVA	Fuliya	THS	10	999	4.934326	0.006	0.009
			Fuliya	Tabgha	12	999	1.200319	0.213	0.213
			THS	Tabgha	16	999	6.764125	0.001	0.003
**Jaccard**	Biofilm	PERMANOVA	Fuliya	THS	10	999	4.985157	0.006	0.009
			Fuliya	Tabgha	12	999	1.433685	0.047	0.047
			THS	Tabgha	16	999	5.849448	0.001	0.003
**Bray-Curtis**	Water	PERMDISP	Fuliya	THS	12	999	0.459518	0.527	0.527
			Fuliya	Haon-Borehole	8	999	1.815226	0.03	0.086
			Fuliya	Tabgha	23	999	6.624098	0.139	0.2085

			THS	Haon-Borehole	12	999	4.142689	0.012	0.072
			THS	Tabgha	27	999	1.691002	0.359	0.4308
			Haon-Borehole	Tabgha	23	999	17.05245	0.043	0.086
**Jaccard**	Water	PERMDISP	Fuliya	THS	12	999	0.664706	0.451	0.451
			Fuliya	Haon-Borehole	8	999	2.983409	0.031	0.09
			Fuliya	Tabgha	23	999	15.43231	0.081	0.1215
			THS	Haon-Borehole	12	999	5.616507	0.008	0.048
			THS	Tabgha	27	999	3.524718	0.269	0.3228
			Haon-Borehole	Tabgha	23	999	39.0096	0.045	0.09
**Bray-Curtis**	Biofilm	PERMDISP	Fuliya	THS	10	999	9.416068	0.033	0.0495
			Fuliya	Tabgha	12	999	0.76546	0.625	0.625
			THS	Tabgha	16	999	20.26223	0.001	0.003
**Jaccard**	Biofilm	PERMDISP	Fuliya	THS	10	999	0.424607	0.408	0.408
			Fuliya	Tabgha	12	999	16.50011	0.164	0.246
			THS	Tabgha	16	999	9.356684	0.001	0.003

Water and biofilm samples were analyzed separately and taken from one of the following spring clusters: Tiberias Hot Springs (THS), Haon-Borehole, Tabgha and Fuliya. Biofilm was not available for Haon-Borehole.

Supplementary references

1. Zuur, A. F., Ieno, E. N., Walker, N., Saveliev, A. A. & Smith, G. M. *Mixed effects models and extensions in ecology with R*. (Springer New York, 2009). doi:10.1007/978-0-387-87458-6.
2. Burnham, K. P. & Anderson, D. R. *Model selection and multimodel inference: A practical information-theoretic approach*. (Springer New York, 2002). doi:10.1007/b97636.
3. Harrison, X. A. *et al.* A brief introduction to mixed effects modelling and multi-model inference in ecology. *PeerJ* **6**, e4794 (2018).
4. Dormann, C. F. *et al.* Collinearity: a review of methods to deal with it and a simulation study evaluating their performance. *Ecography* **36**, 27–46 (2013).
5. Vatansever, C. & Türetgen, I. Survival of Biofilm-Associated *Legionella pneumophila* Exposed to Various Stressors. *Water Environment Research* **87**, 227–232 (2015).
6. Heller, Höller, Süßmuth & Gundermann. Effect of salt concentration and temperature on survival of *Legionella pneumophila*. *Letters in Applied Microbiology* **26**, 64–68 (1998).
7. Bates, D., Mächler, M., Bolker, B. & Walker, S. Fitting Linear Mixed-Effects Models Using lme4. *Journal of Statistical Software* **67**, (2015).
8. Mazerolle, M. J. AICcmodavg: Model selection and multimodel inference based on (Q)AIC(c). (2019).
9. Bergelson, G., Nativ, R. & Bein, A. Salinization and dilution history of ground water discharging into the Sea of Galilee, the Dead Sea Transform, Israel. *Applied Geochemistry* **14**, 91–118 (1999).
10. Moeller, P. *et al.* Relationship of brines in the Kinnarot Basin, Jordan-Dead Sea Rift Valley. *Geofluids* **12**, 166–181 (2012).
